# Identification of sex-biased and neurodevelopment genes *via* brain transcriptome in *Ostrinia furnacalis*

**DOI:** 10.3389/fphys.2022.953538

**Published:** 2022-08-08

**Authors:** Yajun Chang, Bin Yang, Yu Zhang, Chenxi Dong, Lei Liu, Xincheng Zhao, Guirong Wang

**Affiliations:** ^1^ State Key Laboratory for Biology of Plant Diseases and Insect Pests, Institute of Plant Protection, Chinese Academy of Agricultural Sciences, Beijing, China; ^2^ Department of Entomology, College of Plant Protection, Henan Agricultural University, Zhengzhou, China; ^3^ Key Laboratory of Biohazard Monitoring, Green Prevention and Control for Artificial Grassland, Ministry of Agriculture and Rural Affairs, Institute of Grassland Research of Chinese Academy of Agricultural Sciences, Hohhot, China

**Keywords:** PPI network, sex-biased genes, transcriptome analysis, *Ostrinia furnacalis*, brain neurodevelopmental genes

## Abstract

Insect brains play important roles in the regulation of sex-biased behaviors such as mating and oviposition. The neural structure and function of brain differences between males and females have been identified, in which the antenna lobes (AL) showed the most discrepancy, however, the whole repertoire of the genes expressed in the brains and the molecular mechanism of neural signaling and structural development are still unclear. In this study, high-throughput transcriptome analysis of male and female brains was carried on in the Asia corn borer, *Ostrinia furnacalis*, and a total of 39.23 Gb data and 34,092 unigenes were obtained. Among them, 276 genes displayed sex-biased expression by DEG analysis, of which 125 genes were highly expressed in the males and 151 genes were highly expressed in the females. Besides, by homology analysis against genes that have been confirmed to be related to brain neurodevelopment, a total of 24 candidate genes were identified in *O. furnacalis*. In addition, to further screen the core genes that may be important for sex-biased nerve signaling and neurodevelopment, protein-protein interaction networks were constructed for the sex-biased genes and neurodevelopment genes. We identified 10 (*Mhc*, *Mlc1*, *Mlc2*, *Prm*, *Mf*, *wupA*, *TpnC25D*, *fln*, *l(2)efl*, and *Act5C*), 11 (*PPO2*, *GNBP3*, *Spn77Ba*, *Ppn*, *yellow-d2*, *PGRP-LB*, *PGRP-SD*, *PGRP-SC2*, *Hml*, *Cg25C*, and *vkg*) and 8 (*dac*, *wg*, *hh*, *ci*, *run*, *Lim1*, *Rbp9*, and *Bx*) core hub genes that may be related to brain neural development from male-biased, female-biased, and neurodevelopment gene groups. Our results provide a reference for further analysis of the dimorphism of male and female brain structures in agricultural pests.

## Graphical Abstract

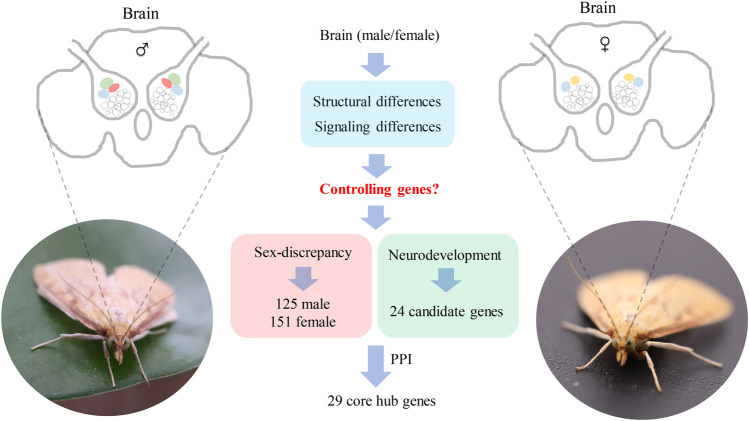

## Introduction

Insects display different behaviors between males and females, and these differences are probably caused by the dimorphism of brain structure and function (Cachero et al., 2010). For example, in Lepidopteran insects, females release sex pheromones and males could detect the pheromones through a sensitive olfactory system (Berg et al., 1995; Hansson et al., 1995; Wang et al., 2018). In the olfactory central nervous system of the brains, the antennal lobe (AL) of males contains a macroglomerular complex (MGC) structure with the main function of sex pheromone recognition (Anton and Homberg, 1999; Zhao and Berg, 2010; Dong et al., 2020). However, the brain structure is quite different between males and females. The female brains have a larger female glomerulus (LFG) instead of MGC, which may function to perceive information related to female-specific behaviors (Rospars and Hildebrand, 2000; Berg et al., 2002; Skiri et al., 2005). The dimorphism of brain structures and functions may be specifically regulated by some genes expressed in the brains, but only a few of them were identified and functionally characterized in insects, especially in agricultural pests.

Sex-biased genes in the brains might be involved in the different neural signaling and structural development. The sex-determining genes *doublesex (dsx)* and *fruitless (fru)* regulate the *Drosophila takeout* gene and affect male courtship behavior (Dauwalder et al., 2002). In addition, some genes displayed similar expression patterns between males and females and they exhibited complex functions in neural development. For example, *dachshund* (*dac*) encoded a new nuclear protein that was necessary for the development of normal eye and mushroom bodies (Mardon et al., 1994; Kurusu et al., 2000). The *scratch (scrt)* was expressed in neuronal precursor cells and encoded a predicted zinc finger transcription factor and it was confirmed to be involved in neuronal development (Roark et al., 1995). However, most of these studies were sporadic and focused on one or two specific genes, while a whole organism view of genes expressed in the brains is still needed (Kasai et al., 1998; Janssens et al*.*, 2010).

The development of sequencing technology has allowed for the collection of whole repertoires of genes expressed in the brains and is beneficial to our understanding of the exhaustive molecular mechanism of neural signaling and structural development of the brains. Transcriptome analyses have been reported in many insects including fruit flies (Zhan et al., 2007; Hughes et al., 2012), bees (Vleurinck et al., 2016; Li et al., 2019; Steffen and Rehan, 2020), ants (Calkins et al., 2018; Romain et al., 2018; Wang et al., 2020), wasps (Berens et al., 2017), butterflies (Zhu et al., 2008; Lugena et al., 2019), silkworm (Wang et al., 2015), and two species of Noctuidae moths (Walker Ⅲ et al., 2019; Cinel and Taylor, 2019). Candidate genes that regulate job differentiation in social insects such as bees and ants and genes related to age and clock in fruit flies have been identified. In the case of *Drosophila melanogaster*, circadian transcriptome analysis of the brain demonstrates that extensive circadian rhythm control of noncoding RNAs (ncRNAs) was involved in circadian rhythm control (Hughes et al., 2012).

Transcriptome data generally contains thousands of genes, and these data can be narrowed down by a determination of the protein-protein interaction (PPI) network which is used to screen and pick up core genes. The PPI network intuitively displays the main characteristics of the interaction and functional characteristics of proteins in birth objects and reflects the unique and essential proteins, which have been widely used in gene function annotation and prediction (Li et al., 2013; Zhang and Zhang, 2020; Xin and Zhang, 2021). STRING (https://STRING-db.org/) is a database that searches for known and predicted interactions between proteins, built through PPI networks to better understand complex regulatory networks in organisms (von Mering et al., 2005; Szklarczyk et al., 2019). In insects, PPI networks are used only in *D. melanogaster* or *Bombyx mori* for screening key proteins related to insecticide resistance, olfactory systems, and detoxification enzymes (Zhang and Zhang, 2019 and 2020; Xin and Zhang, 2020 and 2021).

In the *Ostrinia* genus (Lepidoptera: Crambidae), 21 species have been characterized including serious agricultural pests of maize, the Asian corn borer *Ostrinia furnacalis*, and the European corn borer *Ostrinia nubilalis* (Mutuura and Munroe, 1970; Huang et al., 1998). Olfactory system genes involved in pheromone perception have been identified and functionally analyzed in this genus, indicating that the peripheral nervous system was different between males and females (Ishikawa et al., 1999; Miura et al., 2010; Yang et al., 2015; Liu et al., 2018). As the main olfactory center of the insect brain, AL contains a large number of glomeruli, similar to the olfactory bulb in the brain of vertebrates (Zhao and Berg, 2010). In addition, studies on the brain structure showed that males of *O. nubilalis* have similar large glomerular complexes in the AL, but these large complexes were not found in the females (Karpati et al., 2008). The regulatory gene differences may be related to sex-biased genes or neurodevelopmental genes, but the related candidate genes are completely unknown in this genus.

In this study, high-throughput transcriptome analysis was used to identify the whole repertoire of the genes expressed in the brains of males and females in *O. furnacalis.* Among them, genes with sex-biased expressions were identified by DEG analysis. Neural development genes that were equally expressed between males and females were identified using homology analysis. In addition, PPI network analysis was carried on to further obtain the core hub genes for neural signaling and structural development in the brains. Our results provide a basis for functional studies of the central nervous system of agricultural pests and will help to develop new target genes for pest control in agriculture.

## Materials and methods

### Insect rearing and tissues collection

*O. furnacalis* were maintained in the Chinese Academy of Agricultural Sciences, Beijing, China, under laboratory conditions with an artificial diet at 27 ± 1°C, 16:8 (L:D), and 70% relative humidity. Adults were fed with a 10% sugar solution. Brains from 3-day-old males and females were dissected out in fresh Ringer’s solution (in mM; 150 NaCl, 3 CaCl_2_, 3 KCl, 25 sucrose, and 10 N-Tris [hydroxy-methyl]-methyl-2-amino-ethanesulfonic acid, pH 6.9) on ice, frozen in liquid nitrogen, and stored at −80°C for the following experiments.

### RNA extraction and transcriptome sequencing

Total RNA was isolated from 30 brains of males (or females) using TRIzol reagent (Invitrogen, Carlsbad, CA, United States) according to the manufacturer’s instructions. The RNA was dissolved in RNase-free water, and the quality was assessed by gel electrophoresis. The concentration and purity of RNA were determined on a NanoDrop ND-2000 spectrophotometer (NanoDrop products, Wilmington, DE, United States). A total amount of 1 μg RNA per sample was used as input material for RNA sample preparations. Sequencing libraries were generated using NEBNext®Ultra™ RNA Library Prep Kit for Illumina® (NEB, United States) following the manufacturer’s recommendations and sequenced on an Illumina Hiseq 2000 platform. Three repeats of each sample were used for the sequencing.

### Transcriptome assembly and gene functional annotation

Raw reads were filtered and their qualities were calculated by Q30. Read number, base number, and GC-content were calculated from the filtered clean reads. Trinity was used for the *de novo* assembly (Grabherr et al*.*, 2011). The obtained unigenes were annotated using different databases including NR (Deng et al., 2006), Swiss-Prot (Apweiler et al., 2004), GO (Ashburner et al., 2000), COG (Tatusov et al., 2000), KOG (Koonin et al., 2004), eggNOG (Huerta-Cepas et al., 2016), and KEGG (Kanehisa et al., 2004). The HMMER (Eddy, 1998) software was used to compare with the Pfam (Finn et al., 2014) database to obtain the annotation information of unigenes. BLAST parameter e-values were not greater than 1^−5^, and the HMMER parameter e-value was not greater than 1^−10^.

### Differentially expressed gene analysis

Reads were mapped to the unigenes using Bowtie (Langmead et al., 2009), and the expression level of each unigene was estimated with RSEM (Li and Dewey, 2011). The different expression patterns of unigenes between males and females were calculated with FPKM values by DESeq2 (Anders and Huber, 2010; Trapnell et al., 2010). The generally accepted and effective Benjamini–Hochberg method was used to correct the significant *p*-values obtained by the original hypothesis test. Then, the differentially expressed genes among the sample groups annotated to the GO database were enriched and analyzed by topGO (Alexa and Rahnenfuhrer, 2010) software. In addition, the differentially expressed genes were classified by COG and eggNOG, as well as KEGG annotation and pathway enrichment analysis.

### Identification of developmental genes

Through a literature review, we downloaded the protein sequences of developmental genes from previous studies from NCBI and used them as a query to screen the candidate neural development genes against the unigenes expressed in male and female brains in *O. furnacalis*. By best hit, only the best and longest sequence results were used. The obtained protein sequences were subsequently verified by BLAST orientation in the NCBI database to remove low confidence genes in order to finally obtained the candidate neural development genes in *O. furnacalis.*

### Construction and analysis of PPI network

PPI networks were constructed using STRING 11.5 (https://cn.STRING-db.org) (von Mering et al., 2005). We chose *D. melanogaster* as the organism. The PPI network was optimized and analyzed by Cytoscape 3.7.1 (Shannon et al., 2003). The core modules in the PPI network (Node Score cutoff: 0.2, K-score: 2) were screened by the Molecular Complex Detection (MCODE) plug-in (Bader and Hogue, 2003). The CytoHubba algorithm plug-in was used to find the key targets and subnetworks of PPI networks and determine the hub genes. Finally, the degree and intermediary centrality of each node was calculated by the Network Analyzer plug-in (Brandes, 2008).

## Results

### Sequencing and annotation

By sequencing three cDNA libraries of each sex, more than 131 million reads were obtained, of which 67,102,198 reads and 64,226,235 reads were obtained from female and male brains, respectively (Table 1). The sequencing raw data was uploaded to the NCBI Sequence Read Archive database (Accession number: PRJNA818099). The Q30 values were 94.51%–94.93%, and the overall GC percentages and mapping ratio were 45.01%–46.72% and 82.88%–85.15%, respectively. The assembly results obtained by Trinity are summarized in Table 2. A total of 34,092 unigenes were obtained, with a total length of 42,252,036 bp. The average length and the N50 length of unigenes were 1239.35 bp and 2,352 bp, respectively. According to different annotation methods, 16,174 unigenes were annotated, in which 7,085 unigenes ranged from 300 to 1,000 bp in length, and 9,089 unigenes were over 1,000 bp in length (Table 3).

**TABLE 1:** Summary of brain transcriptomes in *Ostrinia furnacalis.*

Sample	Read number	Base number	GC content (%)	%≥Q30	Mapped reads	Mapped ratio (%)
Female-1	19,943,263	5,964,443,252	45.01	94.64	16,934,654	84.91
Female-2	22,999,277	6,882,726,768	45.22	94.51	19,533,622	84.93
Female-3	24,159,658	7,228,792,424	45.43	94.93	20,572,772	85.15
Male-1	22,095,932	6,577,971,876	45.92	94.80	18,312,073	82.88
Male-2	21,380,928	6,389,253,286	45.46	94.58	18,160,905	84.94
Male-3	20,749,375	6,190,427,112	46.72	94.62	17,350,250	83.62

**TABLE 2:** Assembly summary of brain transcriptomes in *Ostrinia furnacalis.*

Length range	Transcript	Unigene
300–500	21,856 (20.88%)	14,413 (42.28%)
500–1000	20,539 (19.62%)	8,171 (23.97%)
1000–2000	22,716 (21.70%)	5,311 (15.58%)
2000+	39,564 (37.80%)	6,197 (18.18%)
Total Number	104,675	34,092
Total Length	223,008,365	42,252,036
N50 Length	3,644	2,352
Mean Length	2130.48	1239.35

**TABLE 3:** Summary of annotation for the brain transcriptomes in *Ostrinia furnacalis.*

Anno_Database	Annotated_Number	300≤length≤1000	Length≥1000	DEG number
COG_Annotation	4,542	1,482	3,060	79
GO_Annotation	7,508	2,803	4,705	100
KEGG_Annotation	6,750	2,199	4,551	82
KOG_Annotation	9,609	3,096	6,513	123
Pfam_Annotation	11,236	3,780	7,456	169
Swissprot_Annotation	8,565	2,595	5,970	147
eggNOG_Annotation	14,293	5,540	8,753	199
nr_Annotation	15,752	6,689	9,063	214
All_Annotated	16,174	7,085	9,089	220

### Analysis of differentially expressed gene

**FIGURE 1:**
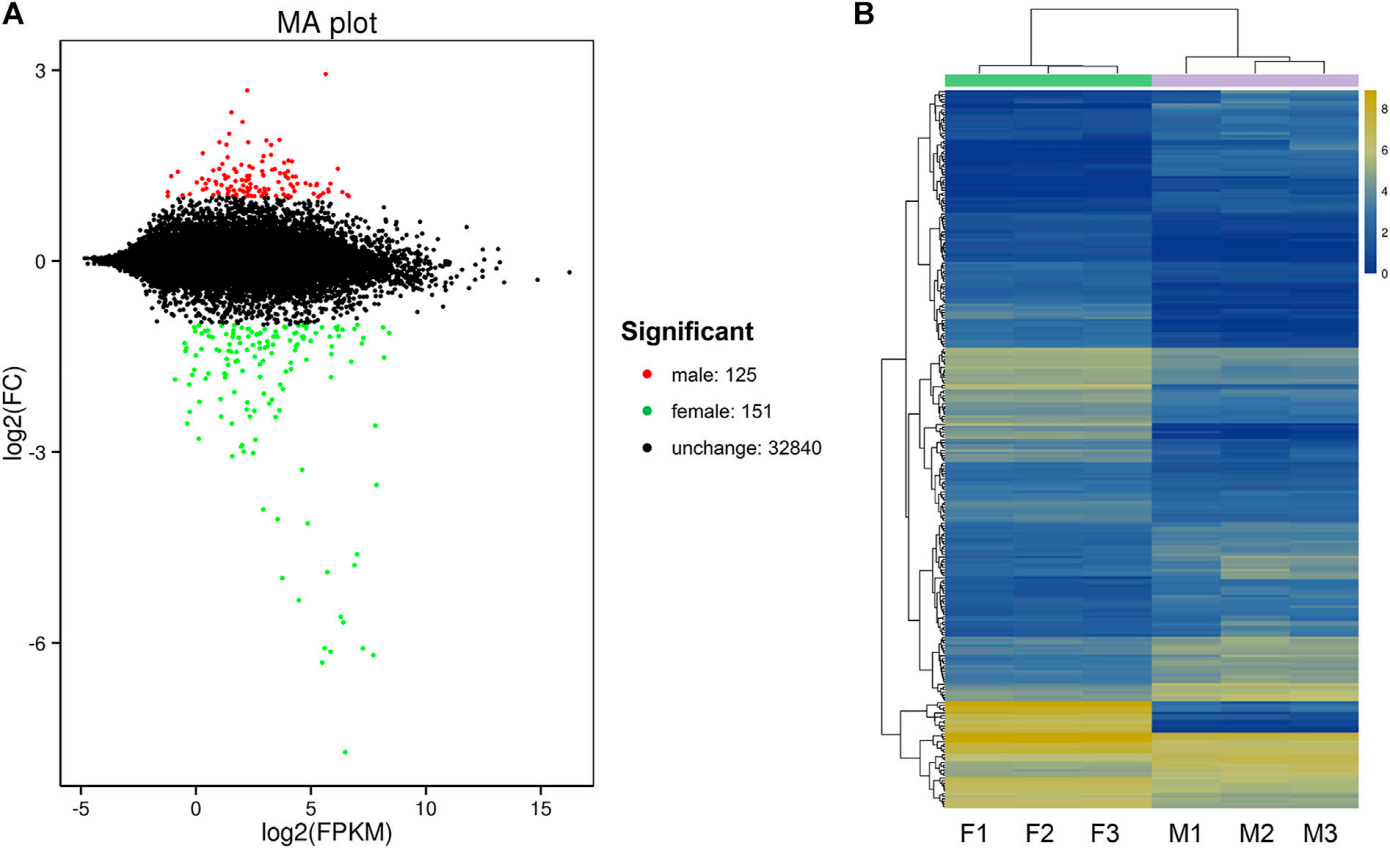
Differentially expressed gene (DEG) analysis for the brain transcriptomes in *Ostrinia furnacalis*. **(A)** MA plot for differentially expressed genes. Each dot in the MA map represents one gene. The abscissa is A value: log2 (FPKM) is the logarithm value of the mean expression quantity of the two samples; ordinate is M value: log2 (FC), is the logarithm value of the multiple of gene expression difference between the two samples, which was used to measure the difference of gene expression. In the picture, green and red dots represent genes with significant differences in expression, green represents downregulation of gene expression, red represents upregulation of gene expression, and black dots represent genes with no significant difference in expression. **(B)** Cluster diagram of expression patterns of DEGs. Different columns represent different samples, and different rows represent different genes. The color represents the logarithm of the FPKM base 2 of the gene expression in the sample. F: female; M: male.

**FIGURE 2:**
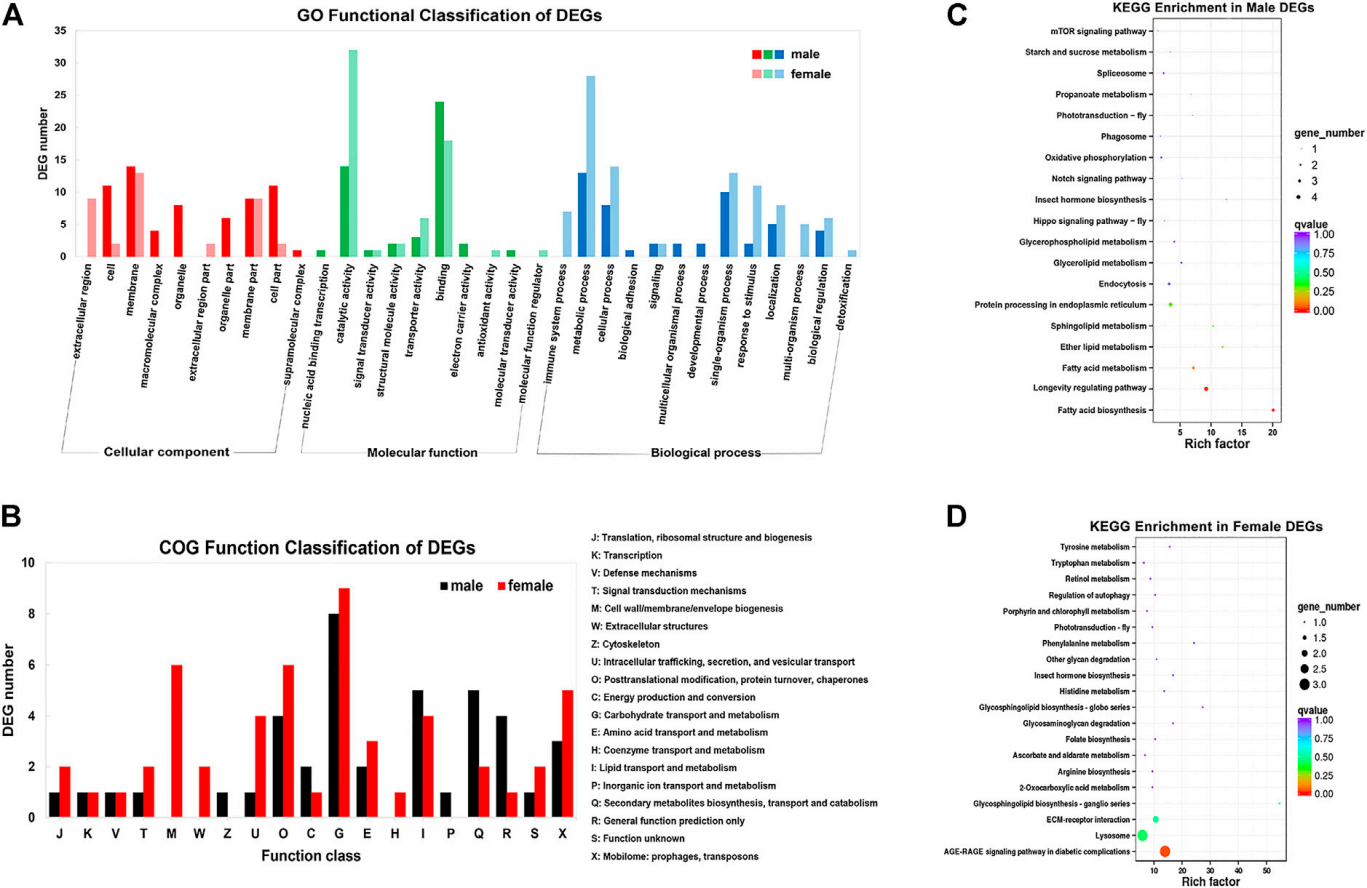
Gene annotation for the unigenes from brain transcriptomes in *Ostrinia furnacalis*. **(A)** GO function classification of DEGs. The abscissa is GO classification and the ordinate is DEG number. This figure shows the gene enrichment of each secondary function of GO under the background of DEG in male and female brains. Dark colors represent DEG in the female brain and light colors represent DEG in the male brain. **(B)** COG function classification of DEGs. The horizontal coordinate is the DEG function class, and the vertical coordinate is the DEG Frequency. Detailed COG notes corresponding to the abscissa are on the right. **(C–D)** KEGG enrichment in male and female DEGs. Each circle represents a KEGG pathway, the ordinate coordinate represents the pathway name, and the abscissa is the enrichment factor, which represents the ratio of the proportion of genes in the differential gene that are annotated to a pathway based on the proportion of genes in all genes that are annotated to that pathway. The larger the enrichment factor, the more significant the level of enrichment of the gene indicating differential expression in that pathway. The color of the circle represents the q value, and the smaller the q value, the more reliable the enrichment significance of the differentially expressed gene in that pathway. The size of the circle indicates the number of genes enriched in the pathway, and the larger the circle, the more genes.

By DEG analysis, 276 genes displayed sex-biased expression, with 125 and 151 highly expressed genes found in the male and female brains, respectively (Figure 1A, Supplementary Table S1). Among them, 6 genes were specifically expressed in females and 15 genes were specifically expressed in males (Table 4), while 18 genes were 50 times different between males and females (Table 5). Through hierarchical clustering analysis, the clustering results of the DEGs among sample groups were obtained (Figure 1B), which again demonstrated the obvious difference between females and males. In addition, 220 of the 276 sex-biased genes were annotated and their functions were analyzed against different databases (Table 3). By GO annotation, 100 genes were clustered into different functional groups including cellular components, molecular function, and biological process, in which cellular components contained more male-biased genes and biological processes contained more female-biased genes (Figure 2A). By COG annotation, 79 genes were clustered into 19 functional groups. Female and male DEGs corresponded to 17 and 16 functional groups, which accounted for the largest proportion in carbohydrate transport and metabolism categories, respectively. Meanwhile, the second-largest category of female DEGs included 6 genes, which specifically corresponded to the cell wall, membrane, or envelope biogenesis (Figure 2B). According to KEGG enrichment analysis, the longevity regulating pathway in males and the AGE-RAGE signaling pathway in diabetic complications in females corresponded to the largest number of genes, and the enrichment results were the most reliable. (Figures 2C,D).

**TABLE 4:** Summary of genes that displayed sex-specific expressions in the brains of *Ostrinia furnacalis.*

Number	Gene id	Female-FPKM (average)	Male-FPKM (average)	Fdr	log2 (FC)	Annotation
1	c34079	157.49	0	1.08E-138	−5.5882	Cecropin family
2	c25808	9.40	0	4.84E-15	−2.2211	Transcription activator MBF2
3	c27820	8.42	0	1.76E-28	−2.9946	peptidoglycan recognition protein
4	c27992	5.51	0	9.83E-06	−1.3075	uncharacterized protein
5	c47702	1.66	0	3.59E-17	−2.3693	Endonuclease-reverse transcriptase
6	c42189	1.49	0	3.57E-06	−1.3667	−
7	c34354	0	0.86	0.00024709	1.0841	myosin heavy chain
8	c41665	0	0.86	0.0006771	1.0202	−
9	c32331	0	0.95	6.33E-06	1.3328	−
10	c38932	0	1.16	1.92E-06	1.4035	Posttranslational modification, protein turnover, chaperones
11	c35757	0	2.05	2.97E-05	1.2355	−
12	c43967	0	2.41	1.21E-05	1.2951	Protease inhibitor/seed storage/LTP family
13	c37529	0	3.38	0.00046178	1.0390	Energy production and conversion; Biological Process: electron transport chain; Cytochrome C oxidase subunit II
14	c40218	0	3.71	3.60E-05	1.2113	Cytochrome C oxidase subunit 1
15	c25751	0	4.06	9.50E-11	1.8680	Lectin C-type domain
16	c45629	0	4.85	1.23E-05	1.2931	Cys-rich Gliadin N-terminal; Protease inhibitor/seed storage/LTP family
17	c44994	0	5.01	2.42E-10	1.8300	Cys-rich Gliadin N-terminal; Protease inhibitor/seed storage/LTP family
18	c44355	0	5.19	1.64E-08	1.6315	PREDICTED: uncharacterized protein
19	c41757	0	5.83	1.10E-16	2.3368	−
20	c41828	0	8.16	1.35E-14	2.1868	Carbohydrate transport and metabolism; Ribulose bisphosphate carboxylase, a small chain
21	c34491	0	9.39	2.98E-22	2.6809	−

**TABLE 5:** Summary of genes that displayed sex-biased expressions (more than 50 times) in the brains of *Ostrinia furnacalis.*

Number	Gene id	Female-FPKM (average)	Male-FPKM (average)	f/m	m/f	Fdr	log2 (FC)	Annotation
1	c33902	457.81	3.44	132.96	0.01	6.56E-40	−3.5166	—
2	c42325	412.00	6.37	64.71	0.02	0	−6.1934	Gloverin-like protein
3	c36790	305.16	0.89	344.17	0.00	2.98E-205	−6.0853	antimicrobial peptide cecropin
4	c48756	178.46	0.64	277.40	0.00	0	−7.7166	vitellogenin
5	c43880	167.88	2.23	75.28	0.01	0	−5.6779	proline-rich protein
6	c25520	115.43	0.17	665.94	0.00	8.79E-192	−6.1372	peptidoglycan recognition protein
7	c44151	96.21	0.49	197.69	0.01	1.62E-232	−6.0821	peptidoglycan recognition protein
8	c42816	89.50	0.51	176.64	0.01	2.39E-292	−6.3075	tenascin-like
9	c33646	44.18	0.21	213.79	0.00	3.40E-136	−5.3300	Trypsin Inhibitor
10	c42870	26.73	0.23	117.91	0.01	8.10E-122	−4.9811	—
11	c38659	23.37	0.10	241.76	0.00	1.03E-61	−4.0577	—
12	c49826	21.64	0.41	52.79	0.02	9.98E-19	−2.4514	—
13	c42325	15.13	0.07	226.90	0.00	2.43E-55	−3.9040	PREDICTED: gloverin-like
14	c42964	5.87	0.03	220.00	0.00	4.23E-20	−2.5502	—
15	c40593	4.18	0.02	179.29	0.01	2.52E-14	−2.1677	—
16	c42506	1.77	0.03	66.25	0.02	8.84E-10	−1.7864	Immunoglobulin domain
17	c27864	0.10	5.35	0.02	55.38	3.40E-12	2.0014	Flightin OS
18	c50115	0.02	1.32	0.02	65.83	0.0006859	1.0338	—

### Identification of candidate neurodevelopment genes in *O. furnacalis*

We obtained the protein sequences of 343 developmental genes expressed in the brains of 163 different species such as *Apis mellifera* (Li et al., 2019), *D. melanogaster* (Dahl et al., 1997; Posnien et al., 2011; Sinigaglia et al., 2013), *Tribolium castaneum* (Choe and Brown, 2007), *Bombyx mori* (Ferguson et al., 2014), and *Bemisia tabaci* (He et al., 2020) from NCBI (Supplementary Table S2). By local BLAST analysis of the sequencing data against the query, 40,285 candidates were obtained from the homology analysis of developmental genes. Subsequently, 2,365 candidates were obtained through the best hit method. In addition, candidates that only had a few matches compared to the query genes were deleted, and the remaining 108 candidates were re-verified by BLAST in the NCBI database. Finally, 24 candidate genes were obtained that could be the neural development genes in *O. furnacalis* (Table 6, Supplementary Table S3).

**TABLE 6:** List of 24 brain neurodevelopment candidate genes identified in *Ostrinia furnacalis*. The citation of Table 6 is missing and must be cited. Please note that Figures and Tables must be cited sequentially.

Number	Ofur	ORF (aa)	Identity (%)	Gene Name	Function	References
1	c47037	354	64	*hu/elav*	nervous system	Satoh et al., 2001; Lowe et al., 2003
2	c48600	362	64			
3	c47752	694	67	*dac*	eye; mushroom bodies	Mardon et al., 1994; Kurusu et al., 2000
4	c46011	446	53	*lim1*	brain; eye	Fujii et al., 1994; Roignant et al., 2010
5	c47353	393	46			
6	c34127	268	42			
7	c29331	229	95	*Wnt*	signals of nerve axis	Janssens et al., 2010
8	c34286	325	64	*foxQ2*	central brain	Kitzmann et al., 2017
9	c49243	742	55	*Dorsal*	dorsoventral axis	Zeitlinger et al., 2007; Fonseca et al., 2008
10	c43582	421	87	*runt*	nervous system	Butler et al., 1992
11	c42641	337	46	*Phm*	mesoderm	Zeitlinger et al., 2007
12	c49013	1385	49	*ci*	regulating the Hedgehog	Hepker et al., 1997; Amin et al., 1999
13	c49331	548	73	*sim*	central nervous system	Kasai et al., 1998
14	c46786	294	82	*sox1/2/3*	nervous system	Lowe et al., 2003
15	c41255	208	82			
16	c49059	363	71	*cAMP-dependent protein kinase*	long-term memory	Müller, 2000
17	c44784	460	87	*fez1*	head	Posnien et al., 2011
18	c44001	383	53	*hh*	head	Amin et al., 1999; Posnien et al., 2011
19	c44769	260	48	*AQP4*	central nervous system	Scharfman and Binder, 2013
20	c46478	270	46			
21	c48478	618	73	*trp*	visual system	Gutorov et al., 2022
22	c32815	445	57	*tyrosine aminotransferase*	immune	Li et al., 2019
23	c37696	435	54			
24	c47271	685	56	*phenoloxidase subunit A3*	immune	Li et al., 2019

### PPI network analysis for sex-biased genes

**FIGURE 3:**
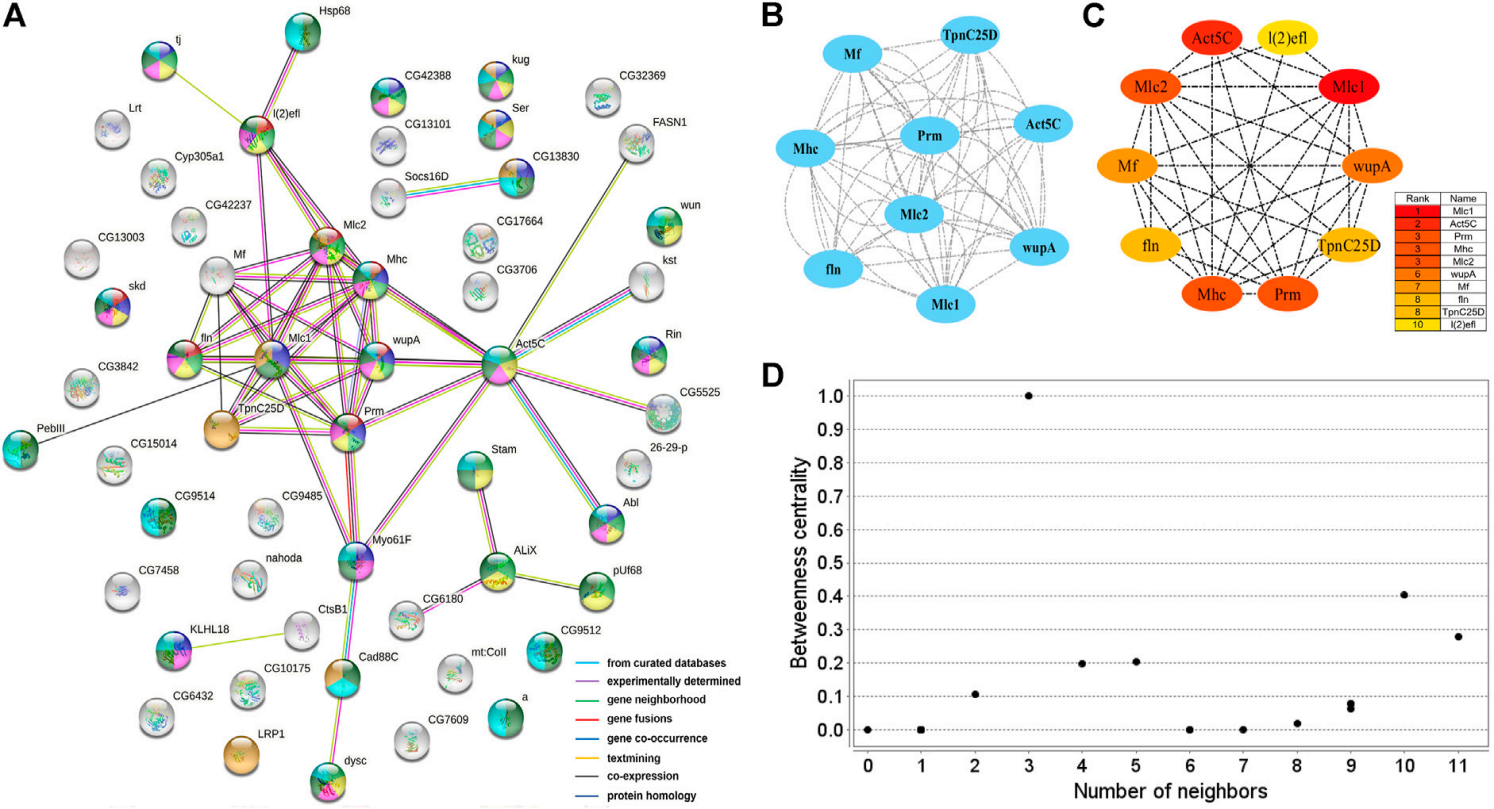
PPI network analysis of 125 male-biased genes in the brains of *Ostrinia furnacalis*. **(A)** PPI network constructed from 125 DEGs in the male brain. Network nodes represent interacting proteins and contain the three-dimensional protein structures that are known or predicted. Red: muscle structure development. Light green: cell development. Dark blue: tissue development. Yellow: cell differentiation. Pink: anatomical structure morphogenesis. Baby blue: multicellular organism development. Dark green: anatomical structure development. Orange: calcium ion binding. The colored border lines indicate the type of evidence that supports these associations. **(B)** PPI core network module obtained by the MCODE algorithm. Nodes represent interacting proteins. Edges represent interactions. **(C)** PPI core proteins were detected by the CytoHubba algorithm. The color is related to the degree of the node, decreasing from red to yellow. The redder the color, the more critical the protein. **(D)** Betweenness centrality analysis of protein nodes. Black nodes represent highly intermediate proteins. The ordinate represents the value of the betweenness centrality. The horizontal axis represents the number of adjacent proteins.

**FIGURE 4:**
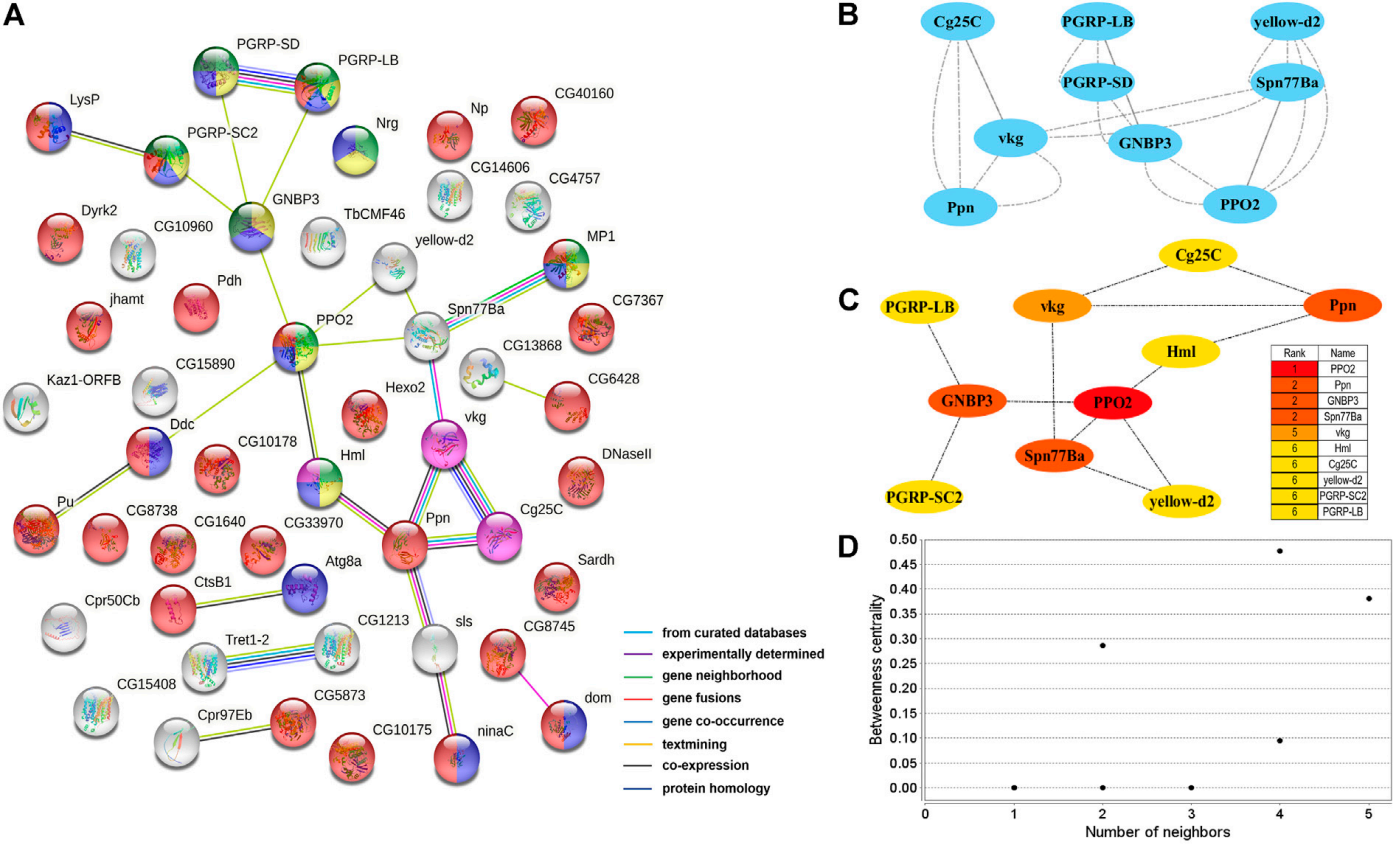
PPI network analysis of 151 female-biased genes in the brains of *Ostrinia furnacalis*. **(A)** PPI network constructed from 151 DEGs in the female brain. Network nodes represent interacting proteins and contain the three-dimensional protein structures that are known or predicted. Light green: innate immune response. Yellow: immune response. Blue: response to external stimulus. Red: catalytic activity. Pink: ECM-receptor interaction. Dark green: Toll and Imd signaling pathway. The colored border lines indicate the type of evidence that supports these associations. **(B)** PPI core network module obtained by the MCODE algorithm. Nodes represent interacting proteins. Edges represent interactions. **(C)** PPI core proteins were detected by the CytoHubba algorithm. The color is related to the degree of the node, decreasing from red to yellow. The redder the color, the more critical the protein. **(D)** Betweenness centrality analysis of protein nodes. Black nodes represent highly intermediate protiens. The ordinate represents the value of the betweenness centrality. The horizontal axis represents the number of adjacent proteins.

Two PPI networks were constructed from 125 male DEGs and 151 female DEGs using the STRING database. Among them, the male network matched 56 nodes and 52 edges (enrichment *p*-value: <1.0^−16^, average node degree: 1.86) (Figure 3A). The female network matched 50 nodes and 25 edges (enrichment *p*-value: <2.15^−10^, average node degree: 1.00) (Figure 4A). Through the MCODE algorithm of Cytoscape, the core interaction network modules that played an important role in the stability of the entire protein interaction network were selected. The core network of males contained 9 hub genes (*Mhc*, *Mlc1*, *Mlc2*, *Prm*, *Mf*, *wupA*, *TpnC25D*, *fln*, and *Act5C*) and 64 interactions (score = 8.000) (Figure 3B). The core network of females also contained 9 hub genes (*PPO2*, *GNBP3*, *Spn77Ba*, *Ppn*, *yellow-d2*, *PGRP-LB*, *PGRP-SD*, *Cg25C*, and *vkg*) and 22 interaction relationships (score = 2.750) (Figure 4B). The CytoHubba plug-in was used again to extract the hub genes (male: *Mhc*, *Mlc1*, *Mlc2*, *Prm*, *Mf*, *wupA*, *TpnC25D*, *fln*, *l(2)efl*, and *Act5C*) (Figure 3C) and (female: *PPO2*, *GNBP3*, *Spn77Ba*, *Ppn*, *yellow-d2*, *PGRP-LB*, *Cg25C*, *PGRP-SC2*, and *Hml*) (Figure 4C). Combining the two algorithms, 125 male DEGs obtained a total of 10 core hub genes, which were mostly described in STRING as related to development and reproduction. 151 female DEGs obtained a total of 11 core hub genes, which were mostly related to immune function (Table 7). When using the NetworkAnalyzer plug-in to calculate the degree and betweenness centrality of each node, the genes with higher degree and betweenness centrality values were basically consistent with the core hub genes we found. In the male network, the degree of *Mlc1* was the highest, and the betweenness centrality of *Act5C* was the highest (Figure 3D, Supplementary Table S4). The degree and the betweenness centrality of *PPO2* were the highest in the female network (Figure 4D, Supplementary Table S5).

**TABLE 7:** 29 core hub genes in the 3 PPI networks of *Ostrinia furnacalis*.

Number	Ofur	Node name	Numbers of description	Description
125 male-biased genes				
1	c26838	Mlc1	5	cell development; tissue development; anatomical structure development; calcium ion binding; supramolecular complex
2	c38747	Act5C	5	cell development; cell differentiation; anatomical structure morphogenesis; multicellular organism development; anatomical structure development
3	c40111	Prm	8	muscle structure development; cell development; tissue development; cell differentiation; anatomical structure morphogenesis; multicellular organism development; anatomical structure development; supramolecular complex
4	c45921	Mhc	8	muscle structure development; cell development; tissue development; cell differentiation; anatomical structure morphogenesis; multicellular organism development; anatomical structure development; supramolecular complex
5	c34456	Mlc2	6	muscle structure development; cell development; cell differentiation; anatomical structure morphogenesis; anatomical structure development; calcium ion binding; supramolecular complex
6	c40474	wupA	8	muscle structure development; cell development; tissue development; cell differentiation; anatomical structure morphogenesis; multicellular organism development; anatomical structure development; supramolecular complex
7	c39951	Mf	1	supramolecular complex
8	c27864	Fln	6	muscle structure development; cell development; cell differentiation; anatomical structure morphogenesis; anatomical structure development; supramolecular complex
9	c39641	TpnC25D	2	calcium ion binding; supramolecular complex
10	c49494	l (2)efl	6	muscle structure development; cell development; cell differentiation; anatomical structure morphogenesis; anatomical structure development; supramolecular complex
156 female-biased genes				
1	c47271	PPO2	6	innate immune response; immune response; response to an external stimulus; catalytic activity; extracellular space; extracellular region
2	c25523	GNBP3	5	immune response; immune response; response to an external stimulus; Toll and Imd signaling pathway; extracellular region
3	c46696	Spn77Ba	3	extracellular space; extracellular space; extracellular region
4	c48631	Ppn	2	catalytic activity; extracellular region
5	c41866	yellow-d2	1	extracellular region
6	c45285	PGRP-LB	6	innate immune response; immune response; response to an external stimulus; catalytic activity; Toll and Imd signaling pathway; extracellular region
7	c25520	PGRP-SD	6	innate immune response; immune response; response to an external stimulus; Toll and Imd signaling pathway; extracellular space; extracellular region
8	c42129	PGRP-SC2	6	innate immune response; immune response; response to external stimulus; catalytic activity; Toll and Imd signaling pathway; extracellular region
9	c49487	Hml	5	innate immune response; immune response; response to external stimulus; ECM-receptor interaction; extracellular region
10	c47432	Cg25C	3	ECM-receptor interaction; extracellular space; extracellular region
11	c46305	Vkg	3	ECM-receptor interaction; extracellular space; extracellular region
24 neurodevelopment candidate genes				
1	c47752	Dac	7	genital disc sexually dimorphic development; central nervous system development; sensory organ development; axonogenesis; neuron differentiation; neuron development; nervous system development
2	c29331	Wg	6	genital disc sexually dimorphic development; ectoderm development; central nervous system development; sensory organ development; neuron differentiation; nervous system development
3	c44001	Hh	5	central nervous system development; immune response; sensory organ development; neuron differentiation; nervous system development
4	c49013	Ci	5	immune response; sensory organ development; neuron differentiation; neuron development; nervous system development
5	c43582	Run	6	central nervous system development; sensory organ development; axonogenesis; neuron differentiation; neuron development; nervous system development
6	c46011	Lim1	3	sensory organ development; neuron differentiation; nervous system development
7	c48600	Rbp9	2	central nervous system development; nervous system development
8	c34127	Bx	1	sensory organ development

### PPI network analysis for neurodevelopment candidate genes

**FIGURE 5:**
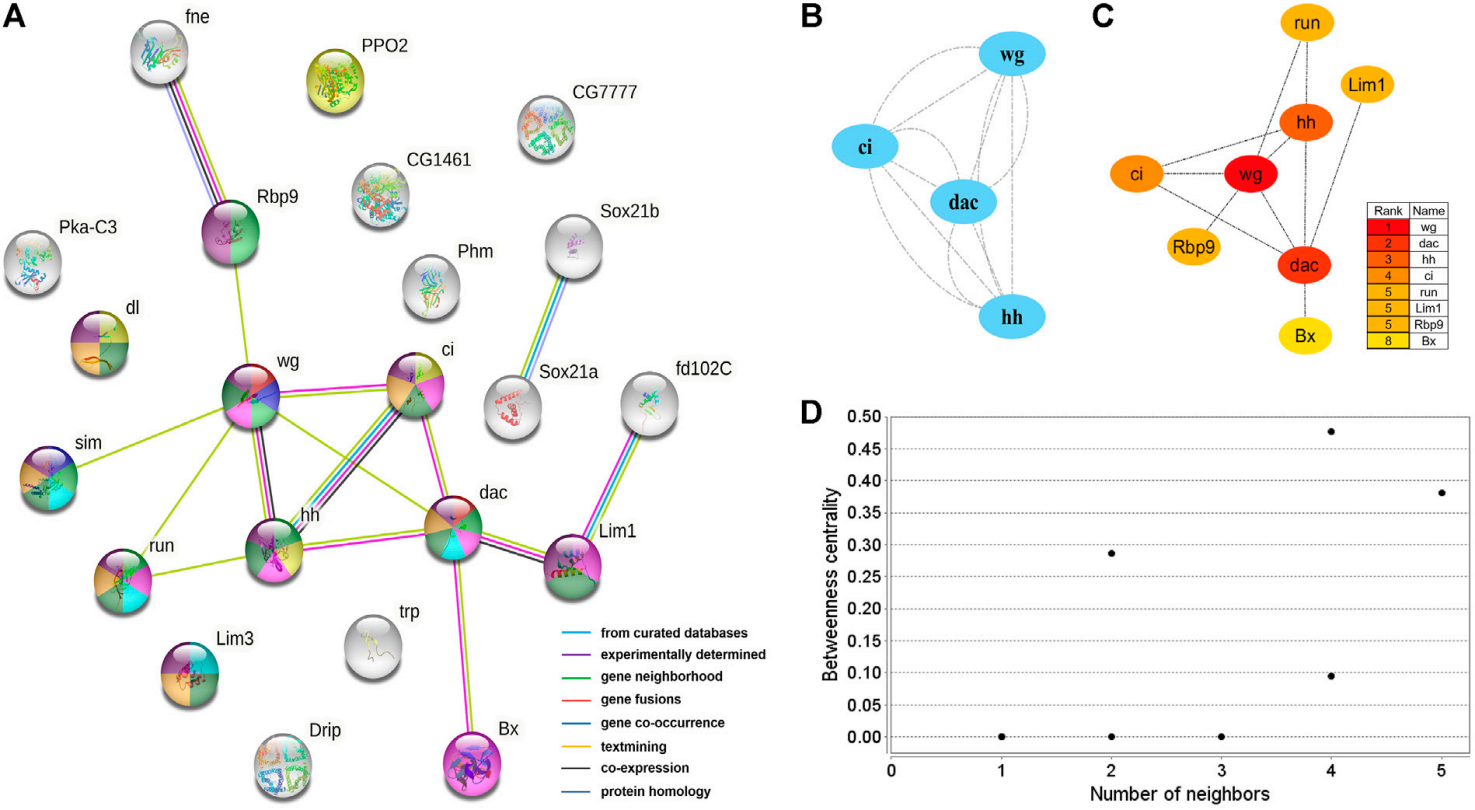
PPI network of 24 brain neurodevelopment genes. **(A)** PPI analysis of 24 candidate genes for brain neurodevelopment. Network nodes represent interacting proteins and contain the three-dimensional protein structures that are known or predicted. Red: genital disc sexually dimorphic development. Dark blue: ectoderm development. Light green: central nervous system development. Yellow: immune response. Pink: sensory organ development. Light blue: axonogenesis. Dark green: neuron differentiation. Orange: neuron development. Prune: nervous system development. The colored border lines indicate the type of evidence that supports these associations. **(B)** PPI core network module obtained by the MCODE algorithm. Nodes represent interacting proteins. Edges represent interactions. **(C)** PPI core proteins were detected by the CytoHubba algorithm. The color is related to the degree of the node, decreasing from red to yellow. The redder the color, the more critical the protein. **(D)** Betweeness centrality analysis of protein nodes. Black nodes represent highly intermediate proteins. The ordinate represents the value of the betweeness centrality. The horizontal axis represents the number of adjacent proteins.

A protein interaction network with 24 nodes and 15 edges (enrichment *p*-value: <1.27^−07^, average node degree: 1.36) was obtained by matching 24 candidate genes for brain neural development with the STRING database (Figure 5A). Cytoscape’s MCODE algorithm was used to select one core network module with close relationships and important roles in the stability of the whole protein interaction network, involving 4 hub genes (*ci*, *wg*, *hh*, and *dac*) and 6 interaction relationships (score = 4.000) (Figure 5B). Furthermore, the CytoHubba plug-in was used to extract 8 hub genes (*dac*, *wg*, *hh*, *ci*, *run*, *Lim1*, *Rbp9*, and *Bx*) from the PPI network by degree (Figure 5C), and compared with the results of MCODE, the 4 highest hub genes were consistent. The two algorithms obtained a total of 8 core hub genes, which were mostly related to neurodevelopmental functions in STRING (Table 7). Using the NetworkAnalyzer plug-in, the highest degree and betweenness centrality was obtained for *wg*, *dac*, *Lim1*, *Rbp9*, and *hh*, which were all included in the 8 hub genes identified in this work (Figure 5D, Supplementary Table S5).

## Discussion

In this study, we identified 276 sex-biased genes and 24 neurodevelopmental candidate genes, of which 29 core genes were screened by PPI network analysis. Most of the 24 neurodevelopmental candidate genes homologous to the functionally known genes were equally expressed between males and females. Interestingly, sex-biased genes displayed significant differences in gene functions between sexes. In the males, sex-biased genes were identified to be associated with development, but female-biased genes were identified to be associated with immunity. One hypothesis to explain this phenomenon is that the normal neurodevelopmental candidate genes might be involved in the basic progress of neurodevelopment so that they were equally expressed in the male and female brains, while the newly identified male-biased genes might be specifically involved in the regulation of development of male-specific MGC structure; this needs to be further confirmed by gene knock-out or knock-down studies.

In the male brains, 125 genes displayed male-specific or biased expressions, of which 11 were identified to be the core genes in the PPI network analysis, and most were functionally associated with development and reproduction (Table 7). The *c26838* gene corresponded to *myosin alkali light chain 1* (*Mlc1*), which had the highest degree in the PPI network analysis, and was shown to be involved in mesodermal development (Picchio et al., 2018). The *c38747* gene corresponds to *Actin-5C* (*Act5C*), which was one of the core hub genes highly expressed in the developing cells, with various functions related to development and reproduction, and it plays important roles in cytokinesis and spermatogenesis (Wagner et al., 2002; Noguchi and Miller, 2003; Wilson et al., 2008; Edwards et al., 2009; Guan et al., 2019). The *c40474* gene corresponds to *wings up A* (*wupA*) and, encodes Troponin I, with involvement in muscle and nervous system development and maintenance (Fishilevich et al., 2019). The *c45921* gene corresponds to *myosin heavy chain* (*Mhc*) and is known to be involved in muscle cell differentiation, cell component movement, and flight (Picchio et al., 2018). These results indicated that the core genes with male-specific or biased expressions might play important roles in the brain neural development and might be related to the development of MGC structure in the male brains in *O. furnacalis*.

In the female brains, 151 genes displayed female-specific or biased expressions, of which 10 were identified to be the core genes in the PPI network analysis, and most were functionally associated with immunity (Table 7). Among them, the *c47271* gene corresponds to *Prophenoloxidase 2* (*PPO2*), which plays an important role in both melanin formation and immunity (Banerjee et al., 2019; Schmid et al., 2019). The *c46696* gene corresponds to *serine protease inhibitor 77Ba* (*Spn77Ba*), which was identified to be involved in the regulation of immune responses by inducing systemic expression of the antifungal peptide drosomycin through the Toll pathway and disruption of tracheal melanosis (Tang et al., 2008). *GNBP3* encodes a hemolymphatic protein, while *PGRP-SD*, *PGRP-SC2,* and *PGRP-LB* encode peptidoglycan-recognition proteins, which are a family of pattern recognition molecules identified to be involved in the regulation of the Toll and Imd signaling pathway. These pathways are related to immune response and development, and correspond corresponding to the *c25523*, *c25520*, *c42129,* and *c45285* genes (Tanji et al., 2007; Royet et al., 2011; Iatsenko et al., 2016; Zhu et al., 2017; Yang et al., 2018; Orlans et al., 2021). Immune genes may be partly related to microglia, which are the resident immune cells in the brain (Hammond et al., 2019). Interestingly, the female-biased genes were mostly related to immunity, but the exact reason for this accumulation of immunity-related genes remains unknown and requires further study. In addition, several peptides (*c25808*, *c34079*, *c36790*, *c42325*) and peptidase (*c33646*) were identified as highly expressed in the female brains, which might be involved in female-specific signaling or the development of the LFG structure in *O. furnacalis*.

Additionally, 24 candidate genes for brain neurodevelopment were identified by homology analysis, and eight of them were identified to be core genes by PPI network analysis. These genes might be associated with neuronal differentiation, nervous system development, cell differentiation, and anatomical morphogenesis in the brain. Among them, only one gene (*Phenoloxidase subunit A3*) displayed female-biased expression and the others were equally expressed between male and female brains. Phenoloxidase is a polycopper oxidase that plays an important role in melanin synthesis, cuticle pigmentation, wound healing, and defense against microbial and parasitic invasion (Elsik et al., 2014). The key core gene *dachshund* (*dac*, *c47752*) was identified being be involved in the development of eyes and mushroom bodies (Mardon et al., 1994; Kurusu et al., 2000). *Hedgehogs* (*hh*, *c44001*) play an important role in the development of head segment polarity (Amin et al., 1999; Ntini and Wimmer, 2011; Posnien et al., 2011), as well as regulation of eye size and pattern (Míguez et al., 2020). In addition, *hh* and *cubitus interruptus* (*ci*, *c49013*) were identified that being involved in regulating the Hedgehog signaling pathway, which is critical in embryonic development and adult tissue homeostasis (Joulia et al., 2005).

## Data Availability

The datasets presented in this study can be found in online repositories. The names of the repository/repositories and accession number(s) can be found below: https://www.ncbi.nlm.nih.gov/, PRJNA818099.
