# Supplementary material for: Identification of sex-biased and neurodevelopment genes via brain transcriptome in Ostrinia furnacalis
Source: Front Physiol. 2022 Aug 8;13:953538. doi: 10.3389/fphys.2022.953538 (PMC9393524; doi:10.3389/fphys.2022.953538)
Supplement: Supplementary file 1 [file DataSheet1.docx]

**Supplementary**

S1: The expression of 276 differences expressed genes (FPKM)

| Number | Gene ID | female-FPKM  (average) | male-FPKM  (average) | f/m | m/f | FDR | log2FC | regulated |
| --- | --- | --- | --- | --- | --- | --- | --- | --- |
| 1 | c46025 | 461.78 | 214.73 | 2.15 | 0.47 | 1.24E-35 | -1.1291 | down |
| 2 | c33902 | 457.81 | 3.44 | 132.96 | 0.01 | 6.56E-40 | -3.5166 | down |
| 3 | c36272 | 428.05 | 151.57 | 2.82 | 0.35 | 1.47E-71 | -1.5144 | down |
| 4 | c42325 | 412.00 | 6.37 | 64.71 | 0.02 | 0 | -6.1934 | down |
| 5 | c43326 | 386.96 | 59.22 | 6.53 | 0.15 | 3.22E-110 | -2.5838 | down |
| 6 | c44523 | 377.49 | 183.91 | 2.05 | 0.49 | 1.80E-32 | -1.0422 | down |
| 7 | c36790 | 305.16 | 0.89 | 344.17 | 0.00 | 2.98E-205 | -6.0853 | down |
| 8 | c38152 | 250.03 | 6.80 | 36.75 | 0.03 | 2.14E-197 | -4.6112 | down |
| 9 | c44148 | 232.25 | 5.78 | 40.20 | 0.02 | 4.88E-245 | -4.7773 | down |
| 10 | c44651 | 216.51 | 94.05 | 2.30 | 0.43 | 2.47E-23 | -1.2056 | down |
| 11 | c45882 | 208.89 | 88.02 | 2.37 | 0.42 | 4.11E-65 | -1.2943 | down |
| 12 | c48756 | 178.46 | 0.64 | 277.40 | 0.00 | 0 | -7.7166 | down |
| 13 | c49043 | 172.33 | 86.60 | 1.99 | 0.50 | 5.07E-42 | -1.0019 | down |
| 14 | c43880 | 167.88 | 2.23 | 75.28 | 0.01 | 0 | -5.6779 | down |
| 15 | c35646 | 161.95 | 53.71 | 3.02 | 0.33 | 1.67E-54 | -1.5779 | down |
| 16 | c49278 | 157.76 | 78.74 | 2.00 | 0.50 | 8.66E-34 | -1.0636 | down |
| 17 | c34079 | 157.49 | 0 | ~ | 0 | 1.08E-138 | -5.5882 | down |
| 18 | c25520 | 115.43 | 0.17 | 665.94 | 0.00 | 8.79E-192 | -6.1372 | down |
| 19 | c47632 | 114.20 | 51.49 | 2.22 | 0.45 | 2.15E-20 | -1.0769 | down |
| 20 | c39294 | 102.66 | 2.14 | 47.90 | 0.02 | 2.12E-191 | -4.8906 | down |
| 21 | c38310 | 101.60 | 46.03 | 2.21 | 0.45 | 1.63E-48 | -1.1774 | down |
| 22 | c44151 | 96.21 | 0.49 | 197.69 | 0.01 | 1.62E-232 | -6.0821 | down |
| 23 | c43969 | 95.84 | 47.52 | 2.02 | 0.50 | 2.02E-16 | -1.0256 | down |
| 24 | c45566 | 94.77 | 45.28 | 2.09 | 0.48 | 2.78E-21 | -1.0723 | down |
| 25 | c25732 | 92.33 | 43.45 | 2.13 | 0.47 | 6.82E-16 | -1.0427 | down |
| 26 | c45698 | 91.00 | 26.15 | 3.48 | 0.29 | 1.70E-77 | -1.8215 | down |
| 27 | c37429 | 89.94 | 29.62 | 3.04 | 0.33 | 3.44E-14 | -1.3381 | down |
| 28 | c42816 | 89.50 | 0.51 | 176.64 | 0.01 | 2.39E-292 | -6.3075 | down |
| 29 | c43755 | 87.56 | 30.75 | 2.85 | 0.35 | 3.72E-39 | -1.4624 | down |
| 30 | c45281 | 78.50 | 34.53 | 2.27 | 0.44 | 1.37E-26 | -1.1880 | down |
| 31 | c46559 | 62.96 | 136.90 | 0.46 | 2.17 | 3.43E-25 | 1.0168 | up |
| 32 | c47560 | 60.28 | 131.79 | 0.46 | 2.19 | 3.11E-37 | 1.0410 | up |
| 33 | c45749 | 59.91 | 29.54 | 2.03 | 0.49 | 2.76E-17 | -1.0389 | down |
| 34 | c43687 | 55.30 | 2.73 | 20.28 | 0.05 | 6.20E-256 | -4.1219 | down |
| 35 | c47139 | 55.00 | 109.60 | 0.50 | 1.99 | 9.39E-24 | 1.0831 | up |
| 36 | c48487 | 49.52 | 24.63 | 2.01 | 0.50 | 3.03E-20 | -1.1282 | down |
| 37 | c49171 | 45.01 | 3.86 | 11.67 | 0.09 | 9.08E-112 | -3.2781 | down |
| 38 | c46490 | 44.85 | 18.75 | 2.39 | 0.42 | 1.39E-31 | -1.2562 | down |
| 39 | c33646 | 44.18 | 0.21 | 213.79 | 0.00 | 3.40E-136 | -5.3300 | down |
| 40 | c37807 | 43.66 | 12.06 | 3.62 | 0.28 | 2.91E-14 | -1.5469 | down |
| 41 | c46211 | 36.90 | 18.49 | 2.00 | 0.50 | 2.27E-18 | -1.0149 | down |
| 42 | c48991 | 36.84 | 18.55 | 1.99 | 0.50 | 1.37E-26 | -1.0549 | down |
| 43 | c47139 | 36.51 | 107.34 | 0.34 | 2.94 | 1.85E-49 | 1.4500 | up |
| 44 | c42114 | 35.63 | 15.34 | 2.32 | 0.43 | 5.41E-26 | -1.2216 | down |
| 45 | c40470 | 34.47 | 16.13 | 2.14 | 0.47 | 1.72E-08 | -1.0038 | down |
| 46 | c44923 | 34.04 | 14.99 | 2.27 | 0.44 | 4.17E-24 | -1.1884 | down |
| 47 | c34084 | 33.14 | 76.07 | 0.44 | 2.30 | 9.15E-15 | 1.0907 | up |
| 48 | c45870 | 32.84 | 18.67 | 1.76 | 0.57 | 1.70E-20 | -1.1876 | down |
| 49 | c38786 | 32.44 | 82.48 | 0.39 | 2.54 | 5.84E-08 | 1.2171 | up |
| 50 | c45194 | 30.59 | 12.18 | 2.51 | 0.40 | 7.57E-23 | -1.3082 | down |
| 51 | c44111 | 29.29 | 11.29 | 2.59 | 0.39 | 3.38E-20 | -1.3345 | down |
| 52 | c44171 | 29.21 | 55.18 | 0.53 | 1.89 | 2.12E-22 | 1.0304 | up |
| 53 | c34477 | 29.15 | 63.44 | 0.46 | 2.18 | 1.05E-13 | 1.0648 | up |
| 54 | c45582 | 29.08 | 14.75 | 1.97 | 0.51 | 3.53E-22 | -1.0139 | down |
| 55 | c46696 | 28.99 | 10.73 | 2.70 | 0.37 | 5.96E-30 | -1.4017 | down |
| 56 | c46806 | 28.56 | 12.52 | 2.28 | 0.44 | 2.06E-13 | -1.1231 | down |
| 57 | c34102 | 27.32 | 11.78 | 2.32 | 0.43 | 5.23E-14 | -1.1368 | down |
| 58 | c49519 | 27.11 | 8.10 | 3.35 | 0.30 | 1.05E-40 | -1.6975 | down |
| 59 | c42870 | 26.73 | 0.23 | 117.91 | 0.01 | 8.10E-122 | -4.9811 | down |
| 60 | c38760 | 26.28 | 7.74 | 3.40 | 0.29 | 2.14E-06 | -1.2452 | down |
| 61 | c47271 | 25.82 | 9.11 | 2.83 | 0.35 | 1.86E-39 | -1.5969 | down |
| 62 | c47573 | 25.48 | 81.86 | 0.31 | 3.21 | 0.000424938 | 1.1250 | up |
| 63 | c37179 | 23.95 | 6.13 | 3.91 | 0.26 | 8.56E-22 | -1.7362 | down |
| 64 | c38659 | 23.37 | 0.10 | 241.76 | 0.00 | 1.03E-61 | -4.0577 | down |
| 65 | c41099 | 23.17 | 4.38 | 5.29 | 0.19 | 3.40E-22 | -2.0087 | down |
| 66 | c39571 | 22.87 | 56.59 | 0.40 | 2.47 | 1.16E-21 | 1.0066 | up |
| 67 | c42129 | 22.10 | 2.68 | 8.25 | 0.12 | 5.59E-27 | -2.3427 | down |
| 68 | c49826 | 21.64 | 0.41 | 52.79 | 0.02 | 9.98E-19 | -2.4514 | down |
| 69 | c39050 | 21.46 | 54.93 | 0.39 | 2.56 | 4.06E-10 | 1.2153 | up |
| 70 | c41726 | 21.32 | 48.22 | 0.44 | 2.26 | 0.000202003 | 1.0572 | up |
| 71 | c43204 | 20.88 | 54.56 | 0.38 | 2.61 | 1.32E-15 | 1.1870 | up |
| 72 | c33301 | 20.48 | 4.91 | 4.17 | 0.24 | 4.17E-37 | -1.9389 | down |
| 73 | c38175 | 20.29 | 9.54 | 2.13 | 0.47 | 3.14E-10 | -1.0393 | down |
| 74 | c37002 | 19.21 | 9.55 | 2.01 | 0.50 | 1.98E-11 | -1.0315 | down |
| 75 | c46233 | 18.19 | 44.46 | 0.41 | 2.44 | 2.03E-30 | 1.1949 | up |
| 76 | c43863 | 17.03 | 2.72 | 6.27 | 0.16 | 2.09E-25 | -2.1767 | down |
| 77 | c41975 | 16.87 | 2.70 | 6.25 | 0.16 | 1.58E-06 | -1.4460 | down |
| 78 | c46806 | 16.12 | 7.19 | 2.24 | 0.45 | 2.21E-12 | -1.1896 | down |
| 79 | c42544 | 15.88 | 6.47 | 2.45 | 0.41 | 6.47E-11 | -1.1838 | down |
| 80 | c40326 | 15.82 | 2.40 | 6.60 | 0.15 | 6.76E-26 | -2.2364 | down |
| 81 | c46246 | 15.73 | 6.35 | 2.48 | 0.40 | 6.63E-11 | -1.1042 | down |
| 82 | c44500 | 15.48 | 39.04 | 0.40 | 2.52 | 6.80E-31 | 1.2226 | up |
| 83 | c42325 | 15.13 | 0.07 | 226.90 | 0.00 | 2.43E-55 | -3.9040 | down |
| 84 | c41402 | 15.09 | 0.38 | 39.37 | 0.03 | 3.06E-13 | -2.0832 | down |
| 85 | c44169 | 14.74 | 5.65 | 2.61 | 0.38 | 1.38E-17 | -1.3118 | down |
| 86 | c36419 | 14.53 | 6.18 | 2.35 | 0.43 | 3.91E-14 | -1.1865 | down |
| 87 | c49699 | 13.13 | 2.86 | 4.59 | 0.22 | 3.68E-06 | -1.3623 | down |
| 88 | c48298 | 13.07 | 25.87 | 0.51 | 1.98 | 1.41E-11 | 1.1001 | up |
| 89 | c44196 | 12.53 | 4.61 | 2.72 | 0.37 | 1.23E-12 | -1.2604 | down |
| 90 | c47432 | 12.46 | 5.04 | 2.47 | 0.40 | 1.62E-36 | -1.3317 | down |
| 91 | c27757 | 12.24 | 1.61 | 7.60 | 0.13 | 4.59E-11 | -1.8192 | down |
| 92 | c25514 | 12.08 | 1.68 | 7.20 | 0.14 | 2.38E-08 | -1.6209 | down |
| 93 | c40776 | 11.71 | 3.40 | 3.44 | 0.29 | 1.02E-15 | -1.5702 | down |
| 94 | c42804 | 11.49 | 0.54 | 21.15 | 0.05 | 1.19E-29 | -2.8072 | down |
| 95 | c47162 | 11.41 | 88.38 | 0.13 | 7.75 | 4.72E-208 | 2.9393 | up |
| 96 | c37967 | 11.31 | 0.31 | 36.48 | 0.03 | 3.22E-17 | -2.3534 | down |
| 97 | c36896 | 11.11 | 25.75 | 0.43 | 2.32 | 6.70E-10 | 1.3263 | up |
| 98 | c42112 | 10.95 | 4.97 | 2.20 | 0.45 | 7.25E-09 | -1.0678 | down |
| 99 | c45424 | 10.88 | 31.65 | 0.34 | 2.91 | 9.45E-10 | 1.2141 | up |
| 100 | c43012 | 10.85 | 23.89 | 0.45 | 2.20 | 1.79E-09 | 1.0068 | up |
| 101 | c49494 | 10.66 | 29.72 | 0.36 | 2.79 | 1.38E-15 | 1.3248 | up |
| 102 | c33905 | 10.65 | 26.53 | 0.40 | 2.49 | 1.23E-13 | 1.1650 | up |
| 103 | c28927 | 10.64 | 0.58 | 18.45 | 0.05 | 3.90E-37 | -3.0167 | down |
| 104 | c36637 | 10.63 | 3.08 | 3.45 | 0.29 | 6.00E-08 | -1.3700 | down |
| 105 | c34688 | 10.19 | 1.65 | 6.17 | 0.16 | 4.07E-07 | -1.5003 | down |
| 106 | c45613 | 10.06 | 3.49 | 2.88 | 0.35 | 1.55E-10 | -1.2811 | down |
| 107 | c43362 | 9.86 | 22.66 | 0.44 | 2.30 | 1.83E-06 | 1.0277 | up |
| 108 | c42680 | 9.68 | 4.21 | 2.30 | 0.44 | 1.77E-11 | -1.1258 | down |
| 109 | c25808 | 9.40 | 0 | ~ | 0 | 4.84E-15 | -2.2211 | down |
| 110 | c48884 | 9.34 | 4.44 | 2.10 | 0.48 | 4.73E-22 | -1.1331 | down |
| 111 | c37958 | 9.25 | 20.71 | 0.45 | 2.24 | 1.92E-07 | 1.0130 | up |
| 112 | c42797 | 8.84 | 1.34 | 6.61 | 0.15 | 6.11E-43 | -2.4416 | down |
| 113 | c46511 | 8.66 | 4.78 | 1.81 | 0.55 | 1.04E-06 | -1.1388 | down |
| 114 | c49429 | 8.58 | 19.50 | 0.44 | 2.27 | 3.18E-13 | 1.0481 | up |
| 115 | c47733 | 8.55 | 27.56 | 0.31 | 3.22 | 9.62E-35 | 1.5668 | up |
| 116 | c39538 | 8.54 | 0.68 | 12.56 | 0.08 | 9.95E-19 | -2.3333 | down |
| 117 | c49487 | 8.48 | 3.88 | 2.19 | 0.46 | 6.47E-22 | -1.1534 | down |
| 118 | c27820 | 8.42 | 0 | ~ | 0 | 1.76E-28 | -2.9946 | down |
| 119 | c49340 | 8.27 | 23.81 | 0.35 | 2.88 | 1.77E-14 | 1.3008 | up |
| 120 | c46305 | 7.86 | 3.28 | 2.40 | 0.42 | 6.75E-26 | -1.3191 | down |
| 121 | c44268 | 7.77 | 22.14 | 0.35 | 2.85 | 7.00E-18 | 1.3313 | up |
| 122 | c43762 | 7.53 | 24.44 | 0.31 | 3.25 | 2.07E-10 | 1.4109 | up |
| 123 | c38945 | 7.50 | 19.36 | 0.39 | 2.58 | 8.69E-17 | 1.1335 | up |
| 124 | c40721 | 7.33 | 25.08 | 0.29 | 3.42 | 1.25E-22 | 1.5784 | up |
| 125 | c45285 | 7.29 | 0.53 | 13.84 | 0.07 | 1.60E-38 | -2.9171 | down |
| 126 | c25523 | 7.22 | 2.47 | 2.92 | 0.34 | 3.24E-06 | -1.1867 | down |
| 127 | c40439 | 7.15 | 21.80 | 0.33 | 3.05 | 6.89E-13 | 1.3777 | up |
| 128 | c41929 | 7.11 | 0.94 | 7.53 | 0.13 | 1.20E-56 | -2.8905 | down |
| 129 | c37669 | 7.09 | 2.21 | 3.20 | 0.31 | 3.34E-09 | -1.3956 | down |
| 130 | c33728 | 6.83 | 27.89 | 0.24 | 4.08 | 0.00011862 | 1.2032 | up |
| 131 | c37595 | 6.71 | 0.85 | 7.86 | 0.13 | 5.29E-05 | -1.2865 | down |
| 132 | c41697 | 6.65 | 2.85 | 2.34 | 0.43 | 3.08E-06 | -1.0629 | down |
| 133 | c48020 | 6.65 | 3.20 | 2.08 | 0.48 | 2.12E-11 | -1.0608 | down |
| 134 | c37687 | 6.42 | 1.73 | 3.71 | 0.27 | 7.28E-22 | -1.7231 | down |
| 135 | c42012 | 6.33 | 22.19 | 0.29 | 3.51 | 9.57E-15 | 1.5452 | up |
| 136 | c28867 | 6.32 | 16.04 | 0.39 | 2.54 | 0.000659335 | 1.0242 | up |
| 137 | c45691 | 6.25 | 9.01 | 0.69 | 1.44 | 4.07E-11 | 1.3443 | up |
| 138 | c42964 | 5.87 | 0.03 | 220.00 | 0.00 | 4.23E-20 | -2.5502 | down |
| 139 | c41950 | 5.79 | 0.82 | 7.04 | 0.14 | 9.75E-08 | -1.5757 | down |
| 140 | c39234 | 5.78 | 0.19 | 30.98 | 0.03 | 1.25E-33 | -3.0655 | down |
| 141 | c43147 | 5.70 | 0.65 | 8.72 | 0.11 | 8.08E-15 | -2.0581 | down |
| 142 | c47815 | 5.68 | 5.14 | 1.10 | 0.91 | 2.09E-08 | -1.1156 | down |
| 143 | c33616 | 5.63 | 1.36 | 4.14 | 0.24 | 5.32E-10 | -1.5638 | down |
| 144 | c40766 | 5.60 | 19.67 | 0.28 | 3.51 | 6.61E-08 | 1.3802 | up |
| 145 | c36622 | 5.55 | 1.71 | 3.24 | 0.31 | 3.78E-05 | -1.2015 | down |
| 146 | c43683 | 5.53 | 2.43 | 2.28 | 0.44 | 3.66E-06 | -1.0472 | down |
| 147 | c27992 | 5.51 | 0 | ~ | 0 | 9.83E-06 | -1.3075 | down |
| 148 | c38290 | 5.46 | 18.61 | 0.29 | 3.41 | 1.21E-05 | 1.2422 | up |
| 149 | c38318 | 5.12 | 0.16 | 31.35 | 0.03 | 3.77E-08 | -1.6306 | down |
| 150 | c47681 | 4.93 | 1.89 | 2.61 | 0.38 | 1.49E-10 | -1.4285 | down |
| 151 | c34456 | 4.78 | 11.90 | 0.40 | 2.49 | 1.54E-05 | 1.0517 | up |
| 152 | c49550 | 4.76 | 1.37 | 3.47 | 0.29 | 0.000317875 | -1.1392 | down |
| 153 | c42724 | 4.72 | 1.06 | 4.45 | 0.22 | 7.68E-07 | -1.4251 | down |
| 154 | c49437 | 4.70 | 11.15 | 0.42 | 2.37 | 9.44E-17 | 1.0895 | up |
| 155 | c40382 | 4.64 | 0.74 | 6.30 | 0.16 | 4.82E-06 | -1.4077 | down |
| 156 | c40008 | 4.57 | 13.13 | 0.35 | 2.87 | 3.02E-05 | 1.1279 | up |
| 157 | c40374 | 4.52 | 1.52 | 2.97 | 0.34 | 2.26E-05 | -1.1896 | down |
| 158 | c41752 | 4.42 | 0.50 | 8.84 | 0.11 | 3.60E-10 | -1.7845 | down |
| 159 | c39978 | 4.41 | 0.40 | 11.03 | 0.09 | 2.28E-10 | -1.8281 | down |
| 160 | c39641 | 4.35 | 10.93 | 0.40 | 2.51 | 9.01E-06 | 1.0634 | up |
| 161 | c34527 | 4.27 | 17.29 | 0.25 | 4.05 | 1.34E-07 | 1.4474 | up |
| 162 | c37877 | 4.25 | 2.09 | 2.03 | 0.49 | 4.05E-05 | -1.0071 | down |
| 163 | c40593 | 4.18 | 0.02 | 179.29 | 0.01 | 2.52E-14 | -2.1677 | down |
| 164 | c41572 | 4.17 | 10.22 | 0.41 | 2.45 | 3.28E-05 | 1.0206 | up |
| 165 | c43518 | 4.03 | 0.25 | 15.89 | 0.06 | 9.46E-21 | -2.4420 | down |
| 166 | c40474 | 4.02 | 11.60 | 0.35 | 2.88 | 7.53E-06 | 1.1397 | up |
| 167 | c25694 | 4.00 | 1.05 | 3.80 | 0.26 | 2.74E-10 | -1.5379 | down |
| 168 | c34654 | 3.92 | 15.99 | 0.25 | 4.08 | 1.04E-07 | 1.4326 | up |
| 169 | c42253 | 3.88 | 15.33 | 0.25 | 3.95 | 2.02E-11 | 1.6676 | up |
| 170 | c45782 | 3.86 | 5.50 | 0.70 | 1.42 | 0.000540867 | 1.0827 | up |
| 171 | c47891 | 3.86 | 9.55 | 0.40 | 2.48 | 2.26E-08 | 1.0961 | up |
| 172 | c48631 | 3.84 | 0.88 | 4.35 | 0.23 | 2.62E-15 | -1.7661 | down |
| 173 | c46451 | 3.82 | 15.44 | 0.25 | 4.05 | 2.43E-43 | 1.8250 | up |
| 174 | c43827 | 3.76 | 1.22 | 3.08 | 0.32 | 0.000109189 | -1.1480 | down |
| 175 | c25498 | 3.67 | 11.41 | 0.32 | 3.11 | 6.90E-11 | 1.3498 | up |
| 176 | c40016 | 3.53 | 0.22 | 15.79 | 0.06 | 5.75E-06 | -1.3908 | down |
| 177 | c49215 | 3.50 | 2.70 | 1.30 | 0.77 | 7.49E-09 | -1.0862 | down |
| 178 | c36148 | 3.25 | 8.39 | 0.39 | 2.59 | 0.000540867 | 1.0186 | up |
| 179 | c48610 | 3.21 | 9.02 | 0.36 | 2.81 | 1.20E-07 | 1.1607 | up |
| 180 | c40604 | 3.19 | 11.52 | 0.28 | 3.61 | 1.73E-21 | 1.5658 | up |
| 181 | c41232 | 3.07 | 1.30 | 2.37 | 0.42 | 0.002548752 | -1.0183 | down |
| 182 | c39167 | 3.04 | 11.69 | 0.26 | 3.84 | 1.55E-08 | 1.4118 | up |
| 183 | c45670 | 3.00 | 7.06 | 0.42 | 2.35 | 1.87E-07 | 1.0286 | up |
| 184 | c33694 | 2.97 | 21.77 | 0.14 | 7.32 | 3.23E-12 | 1.9038 | up |
| 185 | c43634 | 2.96 | 5.98 | 0.49 | 2.02 | 0.001774665 | 1.0145 | up |
| 186 | c46715 | 2.96 | 1.09 | 2.72 | 0.37 | 2.77E-09 | -1.4067 | down |
| 187 | c42645 | 2.79 | 12.52 | 0.22 | 4.49 | 2.49E-12 | 1.6474 | up |
| 188 | c44601 | 2.78 | 6.95 | 0.40 | 2.50 | 4.30E-05 | 1.0269 | up |
| 189 | c33896 | 2.75 | 8.29 | 0.33 | 3.01 | 9.45E-10 | 1.4616 | up |
| 190 | c40377 | 2.75 | 0.17 | 16.52 | 0.06 | 1.89E-09 | -1.7649 | down |
| 191 | c49774 | 2.69 | 0.18 | 14.93 | 0.07 | 0.000632902 | -1.0889 | down |
| 192 | c43456 | 2.68 | 0.61 | 4.36 | 0.23 | 0.000222687 | -1.1945 | down |
| 193 | c40011 | 2.51 | 0.44 | 5.67 | 0.18 | 0.000362348 | -1.1758 | down |
| 194 | c41526 | 2.50 | 7.28 | 0.34 | 2.92 | 6.33E-05 | 1.1111 | up |
| 195 | c41356 | 2.47 | 7.54 | 0.33 | 3.05 | 7.01E-07 | 1.3411 | up |
| 196 | c33881 | 2.37 | 6.05 | 0.39 | 2.55 | 1.08E-06 | 1.0889 | up |
| 197 | c47374 | 2.25 | 7.13 | 0.32 | 3.17 | 4.22E-05 | 1.1511 | up |
| 198 | c47517 | 2.25 | 6.06 | 0.37 | 2.69 | 3.68E-09 | 1.1877 | up |
| 199 | c47529 | 2.22 | 0.47 | 4.72 | 0.21 | 1.12E-16 | -1.8471 | down |
| 200 | c39951 | 2.17 | 7.18 | 0.30 | 3.32 | 4.82E-07 | 1.3331 | up |
| 201 | c37161 | 2.16 | 8.65 | 0.25 | 4.00 | 0.003505072 | 1.0201 | up |
| 202 | c40869 | 2.11 | 11.64 | 0.18 | 5.51 | 0.00096994 | 1.1107 | up |
| 203 | c41866 | 2.06 | 0.58 | 3.53 | 0.28 | 0.000938281 | -1.0924 | down |
| 204 | c26838 | 2.04 | 7.75 | 0.26 | 3.80 | 1.04E-05 | 1.2819 | up |
| 205 | c33501 | 2.01 | 0.21 | 9.42 | 0.11 | 3.81E-15 | -2.2127 | down |
| 206 | c40788 | 1.96 | 0.22 | 8.91 | 0.11 | 2.80E-25 | -2.7902 | down |
| 207 | c37839 | 1.92 | 4.80 | 0.40 | 2.50 | 0.00042896 | 1.0011 | up |
| 208 | c38663 | 1.91 | 0.09 | 21.19 | 0.05 | 9.53E-07 | -1.4782 | down |
| 209 | c42966 | 1.85 | 6.15 | 0.30 | 3.32 | 9.32E-07 | 1.2743 | up |
| 210 | c42408 | 1.83 | 8.21 | 0.22 | 4.48 | 4.73E-08 | 1.4901 | up |
| 211 | c37452 | 1.82 | 7.84 | 0.23 | 4.32 | 1.46E-24 | 1.8680 | up |
| 212 | c45921 | 1.79 | 6.09 | 0.29 | 3.40 | 1.30E-23 | 1.5124 | up |
| 213 | c38763 | 1.78 | 8.56 | 0.21 | 4.81 | 0.000150053 | 1.2103 | up |
| 214 | c42506 | 1.77 | 0.03 | 66.25 | 0.02 | 8.84E-10 | -1.7864 | down |
| 215 | c45694 | 1.74 | 0.64 | 2.74 | 0.37 | 3.93E-05 | -1.1367 | down |
| 216 | c44193 | 1.68 | 0.50 | 3.39 | 0.30 | 0.003817907 | -1.0135 | down |
| 217 | c39172 | 1.68 | 7.35 | 0.23 | 4.39 | 5.38E-05 | 1.2641 | up |
| 218 | c47702 | 1.66 | 0 | ~ | 0 | 3.59E-17 | -2.3693 | down |
| 219 | c39241 | 1.60 | 0.03 | 48.10 | 0.02 | 2.09E-11 | -1.9367 | down |
| 220 | c42291 | 1.56 | 0.34 | 4.59 | 0.22 | 4.40E-12 | -1.7103 | down |
| 221 | c44639 | 1.54 | 0.82 | 1.87 | 0.53 | 3.37E-06 | -1.3796 | down |
| 222 | c47026 | 1.51 | 5.26 | 0.29 | 3.48 | 0.002005779 | 1.0427 | up |
| 223 | c42189 | 1.49 | 0 | ~ | 0 | 3.57E-06 | -1.3667 | down |
| 224 | c36754 | 1.49 | 0.05 | 31.93 | 0.03 | 1.05E-20 | -2.5512 | down |
| 225 | c45717 | 1.48 | 0.48 | 3.08 | 0.32 | 0.000464208 | -1.1010 | down |
| 226 | c43888 | 1.42 | 0.48 | 2.93 | 0.34 | 0.001021361 | -1.0384 | down |
| 227 | c45298 | 1.40 | 4.92 | 0.29 | 3.50 | 9.32E-07 | 1.1106 | up |
| 228 | c44224 | 1.40 | 5.35 | 0.26 | 3.81 | 3.41E-07 | 1.4011 | up |
| 229 | c45049 | 1.35 | 4.06 | 0.33 | 3.00 | 0.000133009 | 1.1030 | up |
| 230 | c39117 | 1.34 | 6.20 | 0.22 | 4.64 | 0.00086734 | 1.1176 | up |
| 231 | c41695 | 1.33 | 0.13 | 10.50 | 0.10 | 4.89E-06 | -1.4130 | down |
| 232 | c29111 | 1.32 | 0.11 | 12.34 | 0.08 | 3.78E-05 | -1.2893 | down |
| 233 | c48409 | 1.25 | 4.00 | 0.31 | 3.20 | 2.03E-09 | 1.3219 | up |
| 234 | c34052 | 1.21 | 15.50 | 0.08 | 12.77 | 4.59E-11 | 1.8962 | up |
| 235 | c27872 | 1.17 | 5.06 | 0.23 | 4.34 | 0.000800233 | 1.1097 | up |
| 236 | c27837 | 1.13 | 5.46 | 0.21 | 4.83 | 7.18E-05 | 1.2573 | up |
| 237 | c25785 | 1.12 | 4.95 | 0.23 | 4.41 | 0.003600744 | 1.0194 | up |
| 238 | c44049 | 1.11 | 7.12 | 0.16 | 6.39 | 0.00098421 | 1.1013 | up |
| 239 | c40111 | 1.04 | 3.65 | 0.29 | 3.50 | 8.16E-09 | 1.3530 | up |
| 240 | c38859 | 1.03 | 0.03 | 31.00 | 0.03 | 1.60E-10 | -1.8601 | down |
| 241 | c28744 | 0.97 | 4.20 | 0.23 | 4.32 | 0.00060942 | 1.1350 | up |
| 242 | c41651 | 0.88 | 4.88 | 0.18 | 5.53 | 0.000392367 | 1.1710 | up |
| 243 | c40956 | 0.87 | 5.83 | 0.15 | 6.68 | 0.001924423 | 1.0476 | up |
| 244 | c36798 | 0.73 | 5.64 | 0.13 | 7.77 | 2.49E-06 | 1.4481 | up |
| 245 | c42603 | 0.64 | 2.73 | 0.24 | 4.24 | 1.57E-07 | 1.4251 | up |
| 246 | c28992 | 0.58 | 1.89 | 0.31 | 3.27 | 0.000150391 | 1.1199 | up |
| 247 | c45843 | 0.57 | 2.37 | 0.24 | 4.19 | 1.89E-05 | 1.2735 | up |
| 248 | c27742 | 0.43 | 9.76 | 0.04 | 22.52 | 0.000194659 | 1.1463 | up |
| 249 | c36990 | 0.41 | 2.27 | 0.18 | 5.58 | 0.000189168 | 1.2144 | up |
| 250 | c40956 | 0.40 | 4.04 | 0.10 | 10.19 | 4.97E-07 | 1.5270 | up |
| 251 | c40120 | 0.35 | 3.63 | 0.10 | 10.48 | 0.001141385 | 1.0594 | up |
| 252 | c48053 | 0.34 | 2.39 | 0.14 | 7.03 | 0.000612462 | 1.1409 | up |
| 253 | c44076 | 0.34 | 3.70 | 0.09 | 10.88 | 2.49E-06 | 1.4488 | up |
| 254 | c39526 | 0.29 | 3.32 | 0.09 | 11.33 | 7.70E-05 | 1.2483 | up |
| 255 | c41941 | 0.29 | 2.17 | 0.14 | 7.39 | 2.66E-09 | 1.6951 | up |
| 256 | c49820 | 0.27 | 3.61 | 0.07 | 13.38 | 0.000705838 | 1.0830 | up |
| 257 | c40402 | 0.26 | 1.41 | 0.18 | 5.49 | 0.002215571 | 1.0517 | up |
| 258 | c38886 | 0.23 | 4.09 | 0.06 | 17.77 | 0.000125158 | 1.1935 | up |
| 259 | c38747 | 0.15 | 5.05 | 0.03 | 33.67 | 0.000632198 | 1.0550 | up |
| 260 | c27864 | 0.10 | 5.35 | 0.02 | 55.38 | 3.40E-12 | 2.0014 | up |
| 261 | c50115 | 0.02 | 1.32 | 0.02 | 65.83 | 0.000685948 | 1.0338 | up |
| 262 | c34354 | 0 | 0.86 | 0 | ~ | 0.000247094 | 1.0841 | up |
| 263 | c41665 | 0 | 0.86 | 0 | ~ | 0.000677097 | 1.0202 | up |
| 264 | c32331 | 0 | 0.95 | 0 | ~ | 6.33E-06 | 1.3328 | up |
| 265 | c38932 | 0 | 1.16 | 0 | ~ | 1.92E-06 | 1.4035 | up |
| 266 | c35757 | 0 | 2.05 | 0 | ~ | 2.97E-05 | 1.2355 | up |
| 267 | c43967 | 0 | 2.41 | 0 | ~ | 1.21E-05 | 1.2951 | up |
| 268 | c37529 | 0 | 3.38 | 0 | ~ | 0.000461782 | 1.0390 | up |
| 269 | c40218 | 0 | 3.71 | 0 | ~ | 3.60E-05 | 1.2113 | up |
| 270 | c25751 | 0 | 4.06 | 0 | ~ | 9.50E-11 | 1.8680 | up |
| 271 | c45629 | 0 | 4.85 | 0 | ~ | 1.23E-05 | 1.2931 | up |
| 272 | c44994 | 0 | 5.01 | 0 | ~ | 2.42E-10 | 1.8300 | up |
| 273 | c44355 | 0 | 5.19 | 0 | ~ | 1.64E-08 | 1.6315 | up |
| 274 | c41757 | 0 | 5.83 | 0 | ~ | 1.10E-16 | 2.3368 | up |
| 275 | c41828 | 0 | 8.16 | 0 | ~ | 1.35E-14 | 2.1868 | up |
| 276 | c34491 | 0 | 9.39 | 0 | ~ | 2.98E-22 | 2.6809 | up |

S2: 343 developmental gene tables in the brain

| Number | Gene ID | Gene | Species |
| --- | --- | --- | --- |
| 1 | GB18323 | abaecin | *Apis mellifera* |
| 2 | GB53576 | apisimin | *Apis mellifera* |
| 3 | GB41428 | defensin 1 | *Apis mellifera* |
| 4 | GB43007 | glucose dehydrogenase | *Apis mellifera* |
| 5 | GB44192 | leucine-rich repeat-containing protein 26 | *Apis mellifera* |
| 6 | GB43738 | phenoloxidase subunit A3 | *Apis mellifera* |
| 7 | GB45969 | tyrosine aminotransferase | *Apis mellifera* |
| 8 | GB46297 | cuticular protein 14 | *Apis mellifera* |
| 9 | GB40148 | cytochrome b561 | *Apis mellifera* |
| 10 | GB49876 | cytochrome P450 6a2 | *Apis mellifera* |
| 11 | GB43713 | cytochrome P450 9e2 | *Apis mellifera* |
| 12 | XP_026300653.1 | esterase A2 | *Apis mellifera* |
| 13 | NP_001128419.1 |  | *Apis mellifera* |
| 14 | GB49885 | cytochrome P450 6a17 | *Apis mellifera* |
| 15 | GB52179 | UDP glucuronosyltransferase2C1 | *Apis mellifera* |
| 16 | XP_026296684.1 | Trp | *Apis mellifera* |
| 17 | XP_001120503.2 |  | *Apis mellifera* |
| 18 | GB51068 | arrestin homolog | *Apis mellifera* |
| 19 | XP_026301344.1 | synaptic vesicular amine | *Apis mellifera* |
| 20 | XP_016772185.1 |  | *Apis mellifera* |
| 21 | XP_392061.3 |  | *Apis mellifera* |
| 22 | XP_026301345.1 |  | *Apis mellifera* |
| 23 | XP_016772186.1 |  | *Apis mellifera* |
| 24 | XP_006563648.1 |  | *Apis mellifera* |
| 25 | GB41643 | Blop | *Apis mellifera* |
| 26 | XP_624531.3 | AQPA | *Apis mellifera* |
| 27 | XP_006569281.1 |  | *Apis mellifera* |
| 28 | NP_001127699.1 | apyrase | *Apis mellifera* |
| 29 | XP_006564869.1 |  | *Apis mellifera* |
| 30 | XP_006564868.1 |  | *Apis mellifera* |
| 31 | XP_006562899.1 | cAMP-dependent protein kinase | *Apis mellifera* |
| 32 | XP_393711.3 |  | *Apis mellifera* |
| 33 | GB46225 | odorant-binding protein | *Apis mellifera* |
| 34 | GB46227 |  | *Apis mellifera* |
| 35 | GB46230 |  | *Apis mellifera* |
| 36 | GB53372 |  | *Apis mellifera* |
| 37 | NP_523601.2 | paxillin | *Drosophila melanogaster* |
| 38 | NP_724185.1 |  | *Drosophila melanogaster* |
| 39 | NP_724183.1 |  | *Drosophila melanogaster* |
| 40 | NP_001246089.1 |  | *Drosophila melanogaster* |
| 41 | NP_724184.2 |  | *Drosophila melanogaster* |
| 42 | NP_001033913.1 |  | *Drosophila melanogaster* |
| 43 | NP_001246089.1 |  | *Drosophila melanogaster* |
| 44 | NP_001260573.1 |  | *Drosophila melanogaster* |
| 45 | NP_001246090.1 |  | *Drosophila melanogaster* |
| 46 | NP_001034513.1 | otd | *Tribolium castaneum* |
| 47 | ACS36120.1 |  | *Acyrthosiphon pisum* |
| 48 | AAD39863.1 | six3 | *Drosophila melanogaster* |
| 49 | NP_001106938.1 | optix | *Tribolium castaneum* |
| 50 | NP_524596.1 | tll | *Drosophila melanogaster* |
| 51 | NP_001034502.1 |  | *Tribolium castaneum* |
| 52 | MK642601.1 |  | *Bemisia tabaci* |
| 53 | CAA81797.1 | lim1 | *Mus musculus* |
| 54 | XP_016038589.1 |  | *Drosophila simulans* |
| 55 | NP_001303305.1 | gsc | *Drosophila simulans* |
| 56 | NP_001137762.2 |  | *Drosophila simulans* |
| 57 | NP_476949.2 |  | *Drosophila simulans* |
| 58 | ABD36164.1 | scro | *Bombyx mori* |
| 59 | NP_001104400.1 |  | *Drosophila melanogaster* |
| 60 | NP_001015473.1 |  | *Drosophila melanogaster* |
| 61 | NP_001104399.1 |  | *Drosophila melanogaster* |
| 62 | NP_001104401.2 |  | *Drosophila melanogaster* |
| 63 | ABA00704.1 | rx | *Tribolium castaneum* |
| 64 | NP_726006.3 |  | *Drosophila melanogaster* |
| 65 | ACD93739.1 | fez1 | *Bombyx mori* |
| 66 | NP_001034065.1 | hh | *Drosophila melanogaster* |
| 67 | NP_001107837.1 |  | *Tribolium castaneum* |
| 68 | ABD24034.1 |  | *Heliconius erato* |
| 69 | AAB28646.1 |  | *Drosophila sp.* |
| 70 | AAA16458.1 |  | *Drosophila melanogaster* |
| 71 | AAA28604.1 |  | *Drosophila melanogaster* |
| 72 | AGJ95047.1 |  | *Bombyx mori* |
| 73 | AFK09796.1 | wg | *Trilophidia annulata* |
| 74 | AAD37798.1 |  | *Schistocerca americana* |
| 75 | NP_523502.1 |  | *Drosophila melanogaster* |
| 76 | AXK92645.1 |  | *Drosophila subpulchrella* |
| 77 | ACB37463.1 | ci | *Ostrinia nubilalis* |
| 78 | ACB37464.1 |  | *Ostrinia nubilalis* |
| 79 | NP_524617.3 |  | *Drosophila melanogaster* |
| 80 | NP_001245402.1 |  | *Drosophila melanogaster* |
| 81 | NP_001245401.1 |  | *Drosophila melanogaster* |
| 82 | AKC58590.1 | irx | *Anomala corpulenta* |
| 83 | XP_001841797.1 |  | *Culex quinquefasciatus* |
| 84 | XP_001841795.1 |  | *Culex quinquefasciatus* |
| 85 | AAB82614.1 | fgf8 | *Danio rerio* |
| 86 | ATU89131.1 | slp | *Dermestes maculatus* |
| 87 | NP_001119728.2 |  | *Bombyx mori* |
| 88 | NP_001071091.1 |  | *Tribolium castaneum* |
| 89 | ABD63010.2 |  | *Tribolium castaneum* |
| 90 | NP_476730.1 |  | *Drosophila melanogaster* |
| 91 | NP_001107793.1 | ems | *Tribolium castaneum* |
| 92 | NP_731868.1 |  | *Drosophila melanogaster* |
| 93 | BAM15951.1 |  | *Bombyx mori* |
| 94 | CAP58696.1 |  | *Tribolium castaneum* |
| 95 | CAA35965.1 |  | *Drosophila melanogaster* |
| 96 | AAD54001.1 | ey | *Drosophila melanogaster* |
| 97 | XP_015044193.1 |  | *Drosophila pseudoobscura* |
| 98 | NP_001014693.1 |  | *Drosophila melanogaster* |
| 99 | NP_001014694.1 |  | *Drosophila melanogaster* |
| 100 | NP_524628.2 |  | *Drosophila melanogaster* |
| 101 | NP_726607.1 |  | *Drosophila melanogaster* |
| 102 | NP_001103907.1 |  | *Tribolium castaneum* |
| 103 | NP_647677.2 | dbx | *Drosophila melanogaster* |
| 104 | XP_001866113.1 |  | *Culex quinquefasciatus* |
| 105 | XP_001866114.1 |  | *Culex quinquefasciatus* |
| 106 | XP_001866115.1 |  | *Culex quinquefasciatus* |
| 107 | XP_001866116.1 |  | *Culex quinquefasciatus* |
| 108 | CBL87030.1 | ptx | *Tribolium castaneum* |
| 109 | NP_733410.2 |  | *Drosophila melanogaster* |
| 110 | NP_996314.1 |  | *Drosophila melanogaster* |
| 111 | NP_001138130.2 |  | *Drosophila melanogaster* |
| 112 | NP_524047.2 | mirr | *Drosophila melanogaster* |
| 113 | NP_729818.1 |  | *Drosophila melanogaster* |
| 114 | NP_001261778.1 |  | *Drosophila melanogaster* |
| 115 | SAP35453.1 | foxQ2 | *Glomeris marginata* |
| 116 | SAP35448.1 |  | *Euperipatoides kanangrensis* |
| 117 | AKU77013.1 |  | *Priapulus caudatus* |
| 118 | AHI16249.1 | fezf | *Platynereis dumerilii* |
| 119 | ADK13096.1 |  | *Branchiostoma lanceolatum* |
| 120 | NP_001158457.1 |  | *Saccoglossus kowalevskii* |
| 121 | NP_001072463.1 |  | *Xenopus tropicalis* |
| 122 | ABX71819.1 | foxA | *Paracentrotus lividus* |
| 123 | AMY99559.1 |  | *Owenia fusiformis* |
| 124 | ARJ36947.1 |  | *Membranipora membranacea* |
| 125 | AHY88466.1 |  | *Terebratalia transversa* |
| 126 | AAC64519.1 | sim | *Drosophila melanogaster* |
| 127 | NP_001262528.1 |  | *Drosophila melanogaster* |
| 128 | NP_524340.2 |  | *Drosophila melanogaster* |
| 129 | NP_731771.3 |  | *Drosophila melanogaster* |
| 130 | NP_001262527.1 |  | *Drosophila melanogaster* |
| 131 | ACF28357.1 | nk | *Drosophila melanogaster* |
| 132 | AUJ87826.1 |  | *Nycteribia kolenatii* |
| 133 | NP_001108550.1 | msx | *Apis mellifera* |
| 134 | BAG11599.1 |  | *Apis mellifera* |
| 135 | AGC29259.1 | dll | *Anopheles gambiae* |
| 136 | AGC29243.1 |  | *Anopheles gambiae* |
| 137 | AMR72029.1 | six3/6 | *Lineus ruber* |
| 138 | AMY99555.1 |  | *Owenia fusiformis* |
| 139 | BBE10622.1 |  | *Peronella japonica* |
| 140 | AGD98926.1 |  | *Nematostella vectensis* |
| 141 | AEZ03831.1 |  | *Terebratalia transversa* |
| 142 | AAO47087.1 | HOX | *Venturia canescens* |
| 143 | AIB07900.1 |  | *Pararge aegeria* |
| 144 | AAQ54690.1 |  | *Mus musculus* |
| 145 | CAA30486.1 |  | *Mus musculus* |
| 146 | AAB28662.2 |  | *Mus sp.* |
| 147 | AAB27153.1 |  | *Mus sp.* |
| 148 | AAB21366.2 |  | *Mus sp.* |
| 149 | AAC60657.2 |  | *Mus sp.* |
| 150 | AAL24809.1 | Otx1 | *Mus musculus* |
| 151 | ABB43131.1 | Pax-6 | *Daphnia pulex* |
| 152 | AAB32671.1 |  | *Rattus sp.* |
| 153 | AAA75363.1 |  | *Paracentrotus lividus* |
| 154 | XP_001848410.1 | OTX | *Culex quinquefasciatus* |
| 155 | AUG89962.1 |  | *Wirenia argentea* |
| 156 | AHY88455.1 |  | *Novocrania anomala* |
| 157 | ADZ24785.1 |  | *Terebratalia transversa* |
| 158 | ABK76302.1 |  | *Hydroides elegans* |
| 159 | ARO85857.1 |  | *Schizocardium californicum* |
| 160 | AAC82470.1 |  | *Petromyzon marinus* |
| 161 | ALJ33545.1 |  | *Clytia hemisphaerica* |
| 162 | AAG59802.1 |  | *Ciona intestinalis* |
| 163 | AAC00193.1 |  | *Branchiostoma floridae* |
| 164 | DAA64848.1 | HOX | *Bombyx mori* |
| 165 | DAA64849.1 |  | *Bombyx mori* |
| 166 | DAA64851.1 |  | *Bombyx mori* |
| 167 | AAO47087.1 |  | *Venturia canescens* |
| 168 | NP_001099831.1 | Pax2 | *Rattus norvegicus* |
| 169 | AWT24644.1 |  | *Protopterus annectens* |
| 170 | CAB09696.1 |  | *Oryzias latipes* |
| 171 | CAA39302.1 |  | *Mus musculus* |
| 172 | NP_001079237.1 | Pax5 | *Xenopus laevis* |
| 173 | AFL02664.1 |  | *Equus caballus* |
| 174 | NP_001102731.1 |  | *Rattus norvegicus* |
| 175 | NP_989755.1 |  | *Gallus gallus* |
| 176 | BAG66241.1 |  | *Carassius langsdorfii* |
| 177 | NP_032808.1 |  | *Mus musculus* |
| 178 | NP_035170.1 | Pax8 | *Mus musculus* |
| 179 | NP_112403.2 |  | *Rattus norvegicus* |
| 180 | NP_001081941.1 |  | *Xenopus laevis* |
| 181 | CAA40725.1 |  | *Mus musculus* |
| 182 | AAU93563.1 | nrp | *Mus musculus* |
| 183 | NP_001013390.1 |  | *Mus musculus* |
| 184 | AAD56625.1 |  | *Rattus norvegicus* |
| 185 | ABB76123.1 |  | *Enterobacter sp.* |
| 186 | CAB3266487.1 | sox1/2/3 | *Phallusia mammillata* |
| 187 | AAP79279.1 |  | *Saccoglossus kowalevskii* |
| 188 | ASW25830.1 | hu/elav | *Branchiostoma lanceolatum* |
| 189 | BAB62225.1 |  | *Branchiostoma belcheri* |
| 190 | AAP79277.1 |  | *Saccoglossus kowalevskii* |
| 191 | ASY93124.1 | dlx | *Pinctada fucata* |
| 192 | ADW95343.1 |  | *Paracentrotus lividus* |
| 193 | NP_034183.1 |  | *Mus musculus* |
| 194 | AAH79609.1 |  | *Mus musculus* |
| 195 | AAF20017.1 | vax | *Gallus gallus* |
| 196 | AAP79280.1 |  | *Saccoglossus kowalevskii* |
| 197 | AFP81698.1 |  | *Convolutriloba longifissura* |
| 198 | NP_037225.1 | nkx2-1 | *Rattus norvegicus* |
| 199 | NP_033411.3 |  | *Mus musculus* |
| 200 | AAG17405.1 |  | *Xenopus laevis* |
| 201 | AAP79291.1 |  | *Saccoglossus kowalevskii* |
| 202 | NP_001071091.1 | bf-1 | *Tribolium castaneum* |
| 203 | QCC26071.1 |  | *Bactrocera dorsalis* |
| 204 | NP_523556.1 | paired box | *Drosophila melanogaster* |
| 205 | NP_723721.1 |  | *Drosophila melanogaster* |
| 206 | ABD63009.2 |  | *Tribolium castaneum* |
| 207 | NP_037133.1 | homeobox 6 (pax6) | *Rattus norvegicus* |
| 208 | NP_001231127.1 |  | *Mus musculus* |
| 209 | NP_001231129.1 |  | *Mus musculus* |
| 210 | NP_038655.1 |  | *Mus musculus* |
| 211 | NP_001231130.1 |  | *Mus musculus* |
| 212 | NP_001231131.1 |  | *Mus musculus* |
| 213 | NP_001297073.1 |  | *Mus musculus* |
| 214 | NP_001297074.1 |  | *Mus musculus* |
| 215 | NP_001297075.1 |  | *Mus musculus* |
| 216 | ADG26729.1 | emx | *Platynereis dumerilii* |
| 217 | AVK72346.1 |  | *Meara stichopi* |
| 218 | AZN28541.1 |  | *Convolutriloba longifissura* |
| 219 | NP_001158361.1 |  | *Saccoglossus kowalevskii* |
| 220 | AAW24008.1 |  | *Oikopleura dioica* |
| 221 | ARO85841.1 |  | *Schizocardium californicum* |
| 222 | AAP79299.1 | barH | *Saccoglossus kowalevskii* |
| 223 | ARO85838.1 |  | *Schizocardium californicum* |
| 224 | NP_001163995.1 | otp | *Tribolium castaneum* |
| 225 | NP_001356966.1 |  | *Drosophila melanogaster* |
| 226 | NP_001286654.1 |  | *Drosophila melanogaster* |
| 227 | NP_001286653.1 |  | *Drosophila melanogaster* |
| 228 | NP_001286652.1 |  | *Drosophila melanogaster* |
| 229 | NP_001286651.1 |  | *Drosophila melanogaster* |
| 230 | NP_523799.3 |  | *Drosophila melanogaster* |
| 231 | NP_001286650.1 |  | *Drosophila melanogaster* |
| 232 | NP_001097388.2 |  | *Drosophila melanogaster* |
| 233 | NP_001036563.1 |  | *Drosophila melanogaster* |
| 234 | NP_997416.2 | developing brain | *Mus musculus* |
| 235 | NP_571233.1 |  | *Danio rerio* |
| 236 | NP_001005232.1 |  | *Mus musculus* |
| 237 | NP_001009644.1 |  | *Rattus norvegicus* |
| 238 | BAQ21916.1 | lim1/5 | *Branchiostoma japonicum* |
| 239 | ARO85855.1 |  | *Schizocardium californicum* |
| 240 | ACH57180.1 |  | *Trichoplax adhaerens* |
| 241 | ABJ74434.1 | en | *Drosophila miranda* |
| 242 | XP_002081053.1 |  | *Drosophila simulans* |
| 243 | BAN82731.1 |  | *Drosophila melanogaster* |
| 244 | XP_001861922.1 | homeobox (gbx) | *Culex quinquefasciatus* |
| 245 | XP_002427507.1 |  | *Pediculus humanus corporis* |
| 246 | AUX14872.1 | Hox3 | *Nilaparvata lugens* |
| 247 | APD78496.1 | Hox2 | *Brachionus plicatilis* |
| 248 | QCF47206.1 |  | *Acanthochitona rubrolineata* |
| 249 | QCF47217.1 |  | *Lottia goshimai* |
| 250 | QIV66779.1 |  | *Betula platyphylla* |
| 251 | BBI41245.1 |  | *Anneissia japonica* |
| 252 | AAS77231.1 |  | *Lethenteron camtschaticum* |
| 253 | SJL82045.1 | Hox1 | *Hordeum vulgare subsp. vulgare* |
| 254 | AAX62801.1 |  | *Oikopleura dioica* |
| 255 | APC93964.1 | hox7 | *Acanthochitona crinita* |
| 256 | APD15669.1 |  | *Gymnomenia pellucida* |
| 257 | APD15687.1 |  | *Idiosepius notoides* |
| 258 | APD15694.1 |  | *Lottia cf. kogamogai* |
| 259 | AKV16307.1 |  | *Acanthochitona crinita* |
| 260 | BBI41248.1 |  | *Anneissia japonica* |
| 261 | AFZ94992.1 |  | *Petromyzon marinus* |
| 262 | ADW95345.1 |  | *Paracentrotus lividus* |
| 263 | ARO85850.1 |  | *Schizocardium californicum* |
| 264 | AAY86179.1 | hox11/13 | *Heliocidaris erythrogramma* |
| 265 | AAY86180.1 |  | *Heliocidaris tuberculata* |
| 266 | AAM97600.1 |  | *Holopneustes purpurascens* |
| 267 | NP_523601.2 | Pax | *Drosophila melanogaster* |
| 268 | NP_724185.1 |  | *Drosophila melanogaster* |
| 269 | NP_724183.1 |  | *Drosophila melanogaster* |
| 270 | NP_724186.2 |  | *Drosophila melanogaster* |
| 271 | NP_724184.2 |  | *Drosophila melanogaster* |
| 272 | NP_001033913.1 |  | *Drosophila melanogaster* |
| 273 | NP_001246089.1 |  | *Drosophila melanogaster* |
| 274 | NP_001260573.1 |  | *Drosophila melanogaster* |
| 275 | NP_001246090.1 |  | *Drosophila melanogaster* |
| 276 | NP_729947.2 |  | *Drosophila melanogaster* |
| 277 | AOQ12959.1 |  | *synthetic construct* |
| 278 | AAL24809.1 | Otx1 | *Mus musculus* |
| 279 | ANO53448.1 | sc | *Nilaparvata lugens* |
| 280 | ABB22062.1 |  | *Drosophila erecta* |
| 281 | ABB22063.1 |  | *Drosophila orena* |
| 282 | ABB22064.1 |  | *Drosophila teissieri* |
| 283 | XP_002099669.1 |  | *Drosophila yakuba* |
| 284 | NP_731223.1 | ato | *Drosophila melanogaster* |
| 285 | NP_001128391.1 | hb | *Nasonia vitripennis* |
| 286 | NP_001128392.1 |  | *Nasonia vitripennis* |
| 287 | NP_731267.1 |  | *Drosophila melanogaster* |
| 288 | NP_731268.1 |  | *Drosophila melanogaster* |
| 289 | XP_015038235.1 |  | *Drosophila pseudoobscura* |
| 290 | XP_015038236.1 |  | *Drosophila pseudoobscura* |
| 291 | AXK92633.1 |  | *Drosophila subpulchrella* |
| 292 | NP_476730.1 | slp1 | *Drosophila melanogaster* |
| 293 | ACL83765.1 | hkb | synthetic construct |
| 294 | AAB31017.1 |  | *Drosophila sp.* |
| 295 | AOQ14499.1 |  | synthetic construct |
| 296 | AOQ13204.1 | gsb | synthetic construct |
| 297 | NP_523863.1 |  | *Drosophila melanogaster* |
| 298 | BAP11264.1 |  | *Graphium sarpedon* |
| 299 | NP_001034495.1 | msh | *Tribolium castaneum* |
| 300 | AOO19834.1 |  | *Geomydoecus aurei* |
| 301 | AAC47329.1 |  | *Drosophila melanogaster* |
| 302 | CAA59680.1 |  | *Drosophila melanogaster* |
| 303 | AAB62975.1 |  | *Drosophila melanogaster* |
| 304 | AAW21975.1 |  | *Tribolium castaneum* |
| 305 | AAC97116.1 | ind | *Drosophila melanogaster* |
| 306 | NP_996087.2 |  | *Drosophila melanogaster* |
| 307 | NP_001034494.1 |  | *Tribolium castaneum* |
| 308 | AAW21974.1 |  | *Tribolium castaneum* |
| 309 | NP_476786.2 | vnd | *Drosophila melanogaster* |
| 310 | NP_001036253.1 |  | *Drosophila melanogaster* |
| 311 | AOQ13469.1 |  | synthetic construct |
| 312 | CAC84070.1 | dac | *Tribolium castaneum* |
| 313 | NP_723969.1 |  | *Drosophila melanogaster* |
| 314 | NP_723971.1 |  | *Drosophila melanogaster* |
| 315 | NP_723968.1 |  | *Drosophila melanogaster* |
| 316 | NP_723970.1 |  | *Drosophila melanogaster* |
| 317 | NP_001014486.1 |  | *Drosophila melanogaster* |
| 318 | NP_001014487.1 |  | *Drosophila melanogaster* |
| 319 | AAD31712.1 | toy | *Drosophila melanogaster* |
| 320 | NP_524638.3 |  | *Drosophila melanogaster* |
| 321 | NP_001259080.1 |  | *Drosophila melanogaster* |
| 322 | NP_001368990.1 |  | *Drosophila melanogaster* |
| 323 | AAD27861.1 | cas | *Drosophila melanogaster* |
| 324 | AOQ06889.1 |  | *synthetic construct* |
| 325 | CAB42967.1 |  | *Drosophila melanogaster* |
| 326 | ADD78288.1 | tas 11 | *Pantoea ananatis* |
| 327 | MWRG01050856.1 |  | *Trichonephila clavipes* |
| 328 | EDW67051.1 | lab | *Drosophila virilis* |
| 329 | NP_476613.1 |  | *Drosophila melanogaster* |
| 330 | AOQ02708.1 |  | *synthetic construct* |
| 331 | AOQ14321.1 | klu | *synthetic construct* |
| 332 | NP_477062.1 |  | *Drosophila melanogaster* |
| 333 | NP_001261690.1 |  | *Drosophila melanogaster* |
| 334 | NP_001261691.1 |  | *Drosophila melanogaster* |
| 335 | NP_001287024.1 |  | *Drosophila melanogaster* |
| 336 | CCK18315.1 | Ibe | *Hydraena biltoni* |
| 337 | CCC42331.1 |  | *Eucatops sp.* |
| 338 | QHW04920.1 |  | *Monochamus sp.* |
| 339 | NP_523558.2 | pdm | *Drosophila melanogaster* |
| 340 | NP_723763.1 |  | *Drosophila melanogaster* |
| 341 | NP_001285877.1 |  | *Drosophila melanogaster* |
| 342 | NP_001285878.1 |  | *Drosophila melanogaster* |
| 343 | NP_001285876.1 |  | *Drosophila melanogaster* |
| 344 | CAA82953.1 | runt | *Manduca sexta* |
| 345 | NP_523424.2 |  | *Drosophila melanogaster* |
| 346 | NP_001245786.1 |  | *Drosophila melanogaster* |
| 347 | NP_001285501.1 |  | *Drosophila melanogaster* |
| 348 | ALL54414.1 | vp | *Huechys beata* |
| 349 | AAY55169.1 | unpg | *Drosophila melanogaster* |
| 350 | NP_477146.1 |  | *Drosophila melanogaster* |
| 351 | AAF74349.1 | gcm | *Drosophila melanogaster* |
| 352 | AAC47808.1 |  | *Drosophila melanogaster* |
| 353 | AAC46912.1 |  | *Drosophila melanogaster* |
| 354 | BAL70317.1 | repo | *Camponotus japonicus* |
| 355 | NP_477026.1 |  | *Drosophila melanogaster* |
| 356 | NP_788420.1 | hbn | *Drosophila melanogaster* |
| 357 | AAY51553.1 |  | *Drosophila melanogaster* |
| 358 | ACL84220.1 |  | *synthetic construct* |
| 359 | ARO76891.1 | nk2.1 | *Tuberculatus kuricola* |
| 360 | NP_061244.1 | phc2 | *Mus musculus* |
| 361 | NP_001182059.1 |  | *Mus musculus* |
| 362 | NP_001182012.1 |  | *Mus musculus* |
| 363 | BAA03757.1 | vtn | *Bombyx mori* |
| 364 | AAC36319.1 | vax1 | *Mus musculus* |
| 365 | AAF25690.1 |  | *Rattus norvegicus* |
| 366 | ALF04204.1 | dkk3 | *Dociostaurus kraussi* |
| 367 | AEC32114.1 | fez | *Tribolium castaneum* |
| 368 | ARO76894.1 | Nk3.2 | *Tuberculatus kuricola* |
| 369 | BAV17687.1 | sfrp1/5 | *Patiria pectinifera* |
| 370 | NP_001161656.1 |  | *Saccoglossus kowalevskii* |
| 371 | CAB3266010.1 |  | *Phallusia mammillata* |
| 372 | ACY92646.1 |  | *Saccoglossus kowalevskii* |
| 373 | BAA11115.2 | zic2 | *Mus musculus* |
| 374 | AAH82436.1 |  | *Xenopus laevis* |
| 375 | NP_001101862.2 |  | *Rattus norvegicus* |
| 376 | NP_511100.1 | Nvsp5/Buttonhead | *Drosophila melanogaster* |
| 377 | NP_001107792.1 |  | *Tribolium castaneum* |
| 378 | CAP58434.1 | Optix | *Tribolium castaneum* |
| 379 | NP_524695.2 |  | *Drosophila melanogaster* |
| 380 | NP_724640.1 |  | *Drosophila melanogaster* |
| 381 | NP_001260793.1 |  | *Drosophila melanogaster* |
| 382 | NP_034597.2 | HoxD1 | *Mus musculus* |
| 383 | NP_077312.2 | IRX3 | *Homo sapiens* |
| 384 | NP_001100883.1 |  | *Rattus norvegicus* |
| 385 | ADO34158.1 | Wnt | *Mnemiopsis leidyi* |
| 386 | AHN95659.1 |  | *Helicoverpa armigera* |
| 387 | ABX90060.1 |  | *Amphimedon queenslandica* |
| 388 | ADM87604.1 | FGF | *Strongylocentrotus purpuratus* |
| 389 | XP_001846423.1 | CDX | *Culex quinquefasciatus* |
| 390 | NP_001191242.1 | ankAT-1 | *Strongylocentrotus purpuratus* |
| 391 | BAJ33521.1 |  | *Hemicentrotus pulcherrimus* |
| 392 | AAF61631.1 | Rx | *Rattus norvegicus* |
| 393 | AAB62324.1 |  | *Mus musculus* |
| 394 | QIC35031.1 | foxQ2 | *Parasteatoda tepidariorum* |
| 395 | AKU77013.1 |  | *Priapulus caudatus* |
| 396 | ADI72431.1 | rel/NF-kB | *Haliotis discus discus* |
| 397 | AHM27300.1 | IkB | *Haliotis diversicolor* |
| 398 | AAZ40334.1 |  | *Carcinoscorpius rotundicauda* |
| 399 | AGS12618.1 |  | *Tigriopus japonicus* |
| 400 | ABC02883.1 |  | *Cotesia plutellae bracovirus* |
| 401 | BAI67121.1 | cactus | *Bombyx mori* |
| 402 | AWD92943.1 |  | *Plutella xylostella* |
| 403 | AAA85908.1 |  | *Drosophila melanogaster* |
| 404 | AAG22858.1 | Tc-Dorsal | *Tribolium castaneum* |
| 405 | NP_476859.2 | Notch | *Drosophila melanogaster* |
| 406 | NP_001245510.1 |  | *Drosophila melanogaster* |
| 407 | NP_999185.1 | EGF | *Sus scrofa* |
| 408 | NP_001003094.1 |  | *Canis lupus familiaris* |
| 409 | AAC47427.1 | FGF | *Drosophila melanogaster* |
| 410 | CAA51340.1 |  | *Drosophila melanogaster* |
| 411 | BAM18484.1 |  | *Papilio xuthus* |
| 412 | ACL97675.1 | DPP | *Bombyx mori* |
| 413 | AKG96797.1 |  | *Bombyx mori* |
| 414 | XP_016023151.1 |  | *Drosophila simulans* |
| 415 | NP_001034496.1 | Twist | *Tribolium castaneum* |
| 416 | NP_001034496.1 |  | *Drosophila melanogaster* |
| 417 | NP_001033967.1 |  | *Drosophila melanogaster* |
| 418 | NP_001286752.1 |  | *Drosophila melanogaster* |
| 419 | SVF01454.1 |  | *Limnogonus franciscanus* |
| 420 | NP_476732.1 | snA(A) | *Drosophila melanogaster* |
| 421 | ACL83778.1 |  | *synthetic construct* |
| 422 | AAD22636.1 | mir-1 | *Klebsiella pneumoniae* |
| 423 | CAA36692.1 | Rho | *Drosophila melanogaster* |
| 424 | AMZ00352.1 |  | *Nilaparvata lugens* |
| 425 | AOQ04681.1 |  | synthetic construct |
| 426 | AAT07304.1 | brk | *Anopheles gambiae* |
| 427 | AOQ14915.1 |  | *synthetic construct* |
| 428 | BAA76710.1 |  | *Drosophila melanogaster* |
| 429 | CAC87842.1 | TUI | *Tetranychus urticae* |
| 430 | NP_476732.1 | SNA | *Drosophila melanogaster* |
| 431 | AOQ14572.1 | HTL | *synthetic construct* |
| 432 | NP_732286.1 |  | *Drosophila melanogaster* |
| 433 | NP_524394.2 |  | *Drosophila melanogaster* |
| 434 | NP_732287.1 |  | *Drosophila melanogaster* |
| 435 | ABW39251.1 | Hbr | *Hopliocnema brachycera* |
| 436 | NP_179942.1 | mes3 | *Arabidopsis thaliana* |
| 437 | AOQ12525.1 | CG12177 | synthetic construct |
| 438 | NP_572911.1 |  | *Drosophila melanogaster* |
| 439 | NP_001285222.1 |  | *Drosophila melanogaster* |
| 440 | NP_001285223.1 |  | *Drosophila melanogaster* |
| 441 | NP_651995.1 | ady43A | *Drosophila melanogaster* |
| 442 | XP_016033680.1 | TIN | *Drosophila simulans* |
| 443 | NP_524433.1 |  | *Drosophila melanogaster* |
| 444 | AFP99911.1 | PHM | *Acropora millepora* |
| 445 | AAO92288.2 |  | *Heterodera glycines* |
| 446 | AAQ05971.1 |  | *Drosophila melanogaster* |
| 447 | CAC10531.1 | spl | *Drosophila melanogaster* |
| 448 | AOO86838.1 |  | *Laodelphax striatellus* |
| 449 | AAC03768.1 |  | *Mus musculus* |
| 450 | AAC47293.1 | vn | *Drosophila melanogaster* |
| 451 | QCC25878.1 |  | *Bactrocera dorsalis* |
| 452 | CAA36692.1 | Rho | *Drosophila melanogaster* |
| 453 | AMZ00352.1 |  | *Nilaparvata lugens* |
| 454 | AOQ04681.1 |  | *synthetic construct* |
| 455 | NP_610701.2 | thS | *Drosophila melanogaster* |
| 456 | NP_001246268.1 |  | *Drosophila melanogaster* |
| 457 | QFU78293.1 |  | *Bombus terrestris* |
| 458 | AAA89117.1 | SOG | *Drosophila melanogaster* |
| 459 | XP_002100689.1 |  | *Drosophila yakuba* |
| 460 | NP_476793.1 | ZEN | *Drosophila melanogaster* |
| 461 | NP_001166190.1 |  | *Bombyx mori* |
| 462 | AOQ11589.1 |  | *synthetic construct* |
| 463 | NP_001036813.1 |  | *Tribolium castaneum* |
| 464 | AGB52034.1 | TLD | *Elaeis guineensis* |
| 465 | NP_001104821.1 | run | *Bombyx mori* |
| 466 | NP_523424.2 |  | *Drosophila melanogaster* |
| 467 | NP_001245786.1 |  | *Drosophila melanogaster* |
| 468 | NP_001285501.1 |  | *Drosophila melanogaster* |
| 469 | XP_016032714.1 | kni | *Drosophila simulans* |
| 470 | NP_001121967.1 |  | *Tribolium castaneum* |
| 471 | ADH51739.1 |  | *Episyrphus balteatus* |
| 472 | NP_476793.1 | Zen | *Drosophila melanogaster* |
| 473 | NP_001166190.1 |  | *Bombyx mori* |
| 474 | NP_001036813.1 |  | *Tribolium castaneum* |
| 475 | AIB07875.1 |  | *Callimorpha dominula* |
| 476 | AIB07880.1 |  | *Cameraria ohridella* |
| 477 | XP_016035872.1 | pnt | *Drosophila simulans* |
| 478 | XP_016035873.1 |  | *Drosophila simulans* |
| 479 | XP_016035874.1 |  | *Drosophila simulans* |
| 480 | XP_016035875.1 |  | *Drosophila simulans* |
| 481 | NP_722766.1 | aop | *Drosophila melanogaster* |
| 482 | NP_523455.2 |  | *Drosophila melanogaster* |
| 483 | NP_001259908.1 |  | *Drosophila melanogaster* |
| 484 | NP_001259909.1 |  | *Drosophila melanogaster* |
| 485 | NP_001259910.1 |  | *Drosophila melanogaster* |
| 486 | CAJ26393.1 | argo | *Enterobacteria phage* |
| 487 | NP_511107.1 | 9a | *Drosophila melanogaster* |
| 488 | QJQ42649.1 | CadN | *Epicopeia polydora* |
| 489 | AAG01337.2 | cv-2 | *Drosophila melanogaster* |
| 490 | NP_001034496.1 | twi | *Tribolium castaneum* |
| 491 | NP_001188508.1 |  | *Bombyx mori* |
| 492 | ABB29556.1 |  | *Drosophila orena* |
| 493 | NP_476732.1 | sna | *Drosophila melanogaster* |
| 494 | NP_572354.1 | NF-Yc | *Drosophila melanogaster* |
| 495 | NP_650272.1 | WntD | *Drosophila melanogaster* |
| 496 | AAF68621.1 | X | *Drosophila yakuba* |
| 497 | NP_524513.1 | m8 | *Drosophila melanogaster* |
| 498 | AVZ23120.1 |  | *Ostrinia penitalis* |
| 499 | NP_524735.1 | SoxN | *Drosophila melanogaster* |
| 500 | NP_524735.1 |  | *Drosophila melanogaster* |
| 501 | XP_002104765.1 | tld | *Drosophila simulans* |
| 502 | AOQ15371.1 | scw | *synthetic construct* |
| 503 | NP_001286088.1 |  | *Drosophila melanogaster* |
| 504 | AAA92297.1 | shn | *Drosophila melanogaster* |
| 505 | AAA98814.1 |  | *Drosophila melanogaster* |
| 506 | NP_476732.1 | Snail | *Drosophila melanogaster* |
| 507 | CAB62556.1 |  | *Drosophila melanogaster* |
| 508 | NP_788420.1 | Tc-Homeobrain | *Drosophila melanogaster* |
| 509 | ADG03434.1 |  | *Nematostella vectensis* |
| 510 | BAW33239.1 |  | *Hemicentrotus pulcherrimus* |
| 511 | AEZ03833.1 |  | *Terebratalia transversa* |
| 512 | CAC06429.1 |  | *Drosophila melanogaster* |
| 513 | SAP35454.1 |  | *Glomeris marginata* |
| 514 | AEB22068.1 | Tc-zen1 | *Solanum tuberosum* |
| 515 | NP_001036813.1 |  | *Tribolium castaneum* |
| 516 | NP_476793.1 |  | *Drosophila melanogaster* |
| 517 | AHN95656.1 | NVβ-catenin | *Helicoverpa armigera* |
| 518 | AHY88450.1 | NvSix3/6 | *Novocrania anomala* |
| 519 | BAV17685.1 | NvFrizzled5/8 | *Patiria pectinifera* |
| 520 | AMB26748.1 |  | *Leptochiton asellus* |
| 521 | ALS30889.1 |  | *Platynereis dumerilii* |
| 522 | NP_524542.1 | Drosophila forkhead (fkh) | *Drosophila melanogaster* |
| 523 | NP_001163762.1 |  | *Drosophila melanogaster* |
| 524 | NP_001263038.1 |  | *Drosophila melanogaster* |
| 525 | NP_001287574.1 |  | *Drosophila melanogaster* |
| 526 | NP_001303461.1 |  | *Drosophila melanogaster* |
| 527 | AOQ14790.1 | slp2 | *synthetic construct* |
| 528 | NP_476834.1 |  | *Drosophila melanogaster* |
| 529 | AOQ13329.1 | fd3F | *synthetic construct* |
| 530 | NP_001356931.1 |  | *Drosophila melanogaster* |
| 531 | NP_726889.3 |  | *Drosophila melanogaster* |
| 532 | NP_608369.1 | fd19B | *Drosophila melanogaster* |
| 533 | AOQ10195.1 | fd64A | *synthetic construct* |
| 534 | NP_651951.1 | fd102C | *Drosophila melanogaster* |
| 535 | AOQ14957.1 | fd68A (FoxK) | *synthetic construct* |
| 536 | NP_511071.3 | Ches-1-like (FoxN) | *Drosophila melanogaster* |
| 537 | NP_996362.2 |  | *Drosophila melanogaster* |
| 538 | NP_001259312.1 |  | *Drosophila melanogaster* |
| 539 | NP_001259311.1 |  | *Drosophila melanogaster* |
| 540 | NP_524302.1 | jumu | *Drosophila melanogaster* |
| 541 | AAK97051.1 | bin | *Drosophila melanogaster* |
| 542 | NP_524202.1 | croc | *Drosophila melanogaster* |
| 543 | BAL14565.1 |  | *Bombyx mori* |
| 544 | AOQ10037.1 | fd96Ca | synthetic construct |
| 545 | NP_001287516.1 |  | *Drosophila melanogaster* |
| 546 | NP_524495.1 |  | *Drosophila melanogaster* |
| 547 | CBL87028.1 | Tc-Six4 | *Tribolium castaneum* |
| 548 | AAD39864.1 |  | *Drosophila melanogaster* |
| 549 | CAX64460.1 | Tc-chx/VSx | *Tribolium castaneum* |
| 550 | AAP79291.1 | Tc-Nkx2.1/SCRO | *Saccoglossus kowalevskii* |
| 551 | CAA11493.1 |  | *Gallus gallus* |
| 552 | AWC67559.1 | GAL4 | *Drosophila melanogaster* |
| 553 | ACL83596.1 | Cbp20 | *Hordeum vulgare* |
| 554 | AAD29697.1 |  | *Arabidopsis thaliana* |
| 555 | ALO62022.1 |  | *Brassica juncea var.* |
| 556 | AAQ01226.1 | dlg1 | *Drosophila melanogaster* |
| 557 | XP_017086734.1 | NCBP | *Drosophila eugracilis* |
| 558 | NP_732081.1 | Globin1 | *Drosophila melanogaster* |
| 559 | NP_524369.1 |  | *Drosophila melanogaster* |
| 560 | NP_732082.1 |  | *Drosophila melanogaster* |
| 561 | NP_732083.1 |  | *Drosophila melanogaster* |
| 562 | NP_001163625.1 |  | *Drosophila melanogaster* |
| 563 | NP_001287343.1 |  | *Drosophila melanogaster* |
| 564 | CAX64460.1 | Tc-chx/VSx | *Tribolium castaneum* |
| 565 | NP_689415.1 | Nr2E1 | *Mus musculus* |
| 566 | NP_001106668.1 |  | *Rattus norvegicus* |
| 567 | QEE82518.1 | TLx | *Urechis unicinctus* |
| 568 | ACH89436.1 |  | *Capitella teleta* |
| 569 | BBD75277.1 |  | *Parasteatoda tepidariorum* |
| 570 | ACI16356.1 |  | *Branchiostoma floridae* |
| 571 | ACH68426.1 |  | *Saccoglossus kowalevskii* |
| 572 | AAD10338.1 | Arx | *Mus musculus* |
| 573 | AQU64617.1 |  | *Terebratalia transversa* |
| 574 | NP_001093644.1 |  | *Rattus norvegicus* |
| 575 | BAA85852.1 |  | *Mus musculus* |
| 576 | AAC53129.1 | Rax | *Mus musculus* |
| 577 | NP_001230653.1 |  | *Gallus gallus* |
| 578 | NP_648283.1 | doc1 | *Drosophila melanogaster* |
| 579 | NP_001261612.1 |  | *Drosophila melanogaster* |
| 580 | APV45532.1 | doc2 | *Megaselia abdita* |
| 581 | AAM11543.1 | doc3 | *Drosophila melanogaster* |
| 582 | AAC52697.1 | Tbx2 | *Mus musculus* |
| 583 | NP_853669.1 | Tbx3 | *Rattus norvegicus* |
| 584 | NP_035665.2 |  | *Mus musculus* |
| 585 | NP_932169.1 |  | *Mus musculus* |
| 586 | BAA87864.1 | Tbx6 | *Drosophila melanogaster* |
| 587 | AAC98309.1 | Pdf | *Drosophila melanogaster* |
| 588 | ABB16909.1 | doc | *Tribolium castaneum* |
| 589 | NP_536741.1 | NPF | *Drosophila melanogaster* |
| 590 | NP_001262643.1 |  | *Drosophila melanogaster* |
| 591 | NP_001262642.1 |  | *Drosophila melanogaster* |
| 592 | AOQ14636.1 |  | *synthetic construct* |
| 593 | NP_001037329.1 | sgf1 | *Bombyx mori* |

S3. Nucleic acid sequences of 24 candidate genes for neural development in the brain of *O.furnacalis*

>hu/elav-like 1

ATGTCCAAAGGCGACAGTGAAAACCAGAACGGCTCGGGCGAGGAGTCCAAGACGAACCTGATCATCAACTACCTGCCGCAGAGCATGACGCAGGAGGAGATCCGCAGCCTGTTCTCCAGCATCGGTGAGGTGGAGTCGTGCAAGCTGATCCGCAACAAGGGCGCGGCCTTCCCGGACGCGCTCAACCACGCGCTGCACGGCGGCGGCCAGAGCCTGGGCTACGCGTTCGTCAACTACCACCGCGCCGAGGACGCGGAAAAGGCCATTACAACGCTCAACGGGCTGCGGCTGCAGAACAAGACCATCAAGGTGTCGTACGCGCGGCCCAGCAGCGAGGCCATCAAGGGCGCCAACCTCTACGTGTCCGGCCTGCCCAAGACCATGACGCAGGCCGAGCTGGAGCGCCTCTTCAGCCCGTACGGCCGCATCATCACGTCGCGAATCCTGTGCGAGAACTCCGGCGGGCGGCCCTTCACCGGCGGCGAGCAGGGCCTGTCCAAGGGCGTCGGCTTCATCCGCTTCGACCAGCGCGTGGAAGCGGAGCGCGCCATTCAGGAGCTGAACGGGACAGTGCCGAAGGGAGCGTCGGAGCCGATCACGGTGAAGTTCGCGAACAACCCGAGCAACAACGGCAAGGCGCTGGCGCCGCTGGCCGCCTACCTGCCGGCCGCGCTGCGCTTCCCGGCGCCGCTGGGCCGCTTCAGTTCAGGCAAGTCGCTGCTAGCTATTAACAAGGGCCTCCAGCGCTACAGCCCGCTGGCCGGCGAGCTGTTGGGCGGCGTGCTGCCCGGCGCCGTCGGCTCCGAGTGGTGCATCTTCGTGTACAACCTGGCGCCGGAGACCGAGGAGAACGTGCTCTGGCAGCTCTTCGGGCCCTTCGGCGCCGTGCAGAGCGTCAAAGTGATCCGCGACCTGCAGACCAACAAGTGCAAGGGCTACGGGTTCATCACGATGACCAACTACGACGAGGCGGTGGTCGCCATCCAGTCGCTCAACGGGTACACGCTCGGGAACCGCGTGCTGCAGGTCAGCTTCAAGACCAACAAGATCAAGACGATCTAA

>Hu/elav-like 2

ATGATGGCAAATTCACTTGACACAGTAAACGCGAACCAACCCACGCAAAATGGTAGCAAGGTTCAGCCATGTAATAACGAGTCCAAGACTAATCTCATAGTTAATTATTTGCCGCAAACGATGACTCAGGAAGAGATACGGTCACTGTTCTCGAGCGTCGGCGAAGTAGAAAGTTGCAAGCTAATTAGAGACAAAGTGACAGTCTTTCCTGACCACATCCTGAACGGCCAAAGTCTTGGTTACGCCTTCGTTAATTACCACAAGGCGGAGGACGCGGAGAAGGCTGTGAACACTCTGAATGGATTGAGATTGCAGAACAAAATCATCAAAGTCTCGTATGCTCGACCTAGTTCCGACGCAATCAAAGGCGCAAACCTCTACGTATCAGGTCTACCGAAACATATGACTCAGCAAGAGTTGGAGAAGCTCTTTAGCCCCTACGGAACCATCATCAGCTCACGCATTCTACATGAGAACATGAACGTTGGGCACTTACTCCAGGTGGGAAATGAAGACCAAGGAGGCATTCAGGGCCCTTCAAGAGGCGTCGCGTTCATCCGATACGACCAGCGGTGTGAAGCAGAAGCAGCTATACGTGAGTTGAATGGATCTATACCGCCTGGTGGCACCGGCCCAATTACAGTCAAGTGTGCAAACAATCCAAGCAACCAGAACAAGGCATTAGCTCCATTGGCTGCATACCTGGCTCCAGCTTCGGCACGTCGCTTCGTCGGACCCGCAGGGAAGGCACTGCTTGCTATCAACAAAGGGCTCCAAAGATTTTCTCCCCTGGCTGACCCACTGATCCAAGGTAACGCTCTAGGTGGTTCGGGTTGGTGCATCTTTGTGTACAATATTGGAGCTGACACTGAGGAGAGTATCCTGTGGCAGCTTTTTGGCCCATTCGGTGCCGTCCAGAGTGTTAAAATAATAAGAGATCCTACCACCAACAAATGTAAAGGATATGGGTTCGTCACTATGACTAATTACGATGAAGCGGTAGTCGCGATCCAGTCCTTGAACGGTTACTCACTGAACGGTCAAGTACTGCAGGTCAGCTTCAAAACGAACAAGAGTAAATCTTAA

>dachshund like

ATGGAGTCCGCCGTGGACTCAGCGTCGACCGCGAGCGAGGTGAGCGGCTCCTCCGGGGGCTCGCCGAGGGTGAAGGCGGCGTCGCCGGCGCGGGGCCTGAGCCCGCCGCAGCTGCTGGCGCCTCGCCTGCCGCTGCCGCCGCCGGGCCTGGGCCTGCTGGGCTCGCTGCAGATGATGCACCACTCGCCCCTCGAGCTGATGGCGGCGGCCCACCACCACGGGCCGCCGCGGTACGGCAGCCCACCGCCGATCTCCACCTCCGACCCGTCGGCCAACGAGTGCAAGCTGGTAGACTATCGTGGGCAGAAAGTTGCCGCGTTTATCATTCAGGGTGACACGATGCTATGCCTGCCGCAGGCTTTCGAACTGTTCTTGAAGCACCTCGTCGGAGGGCTGCACACGGTGTACACGAAGCTAAAGAGACTGGACATAGTGCCGCTGGTGTGCAATGTGGAACAGGTGCGCATCCTCCGCGGGCTGGGCGCCATCCAGCCGGGCGTCAACCGCTGCAAGCTGCTCTCCTGCAAGGACTTCGACGTGCTCTACAGGGACTGCACTACAGCAAGACCGGGCCGACCTCCGAAGCGTGCCTCCGGCGTGGGTCTCTCGCTGGCCGCCACGCAGTTCCCTGGACATCCCTTCAAGAAACACCGGCTGGAGAACGGCGAGTACTCGCCGTATGAGAACGGGCACATGAGTGAAATGGCTCGCATGGAGAAGTCGCCGCTGTTAGCCAACGGGTACAATGCCCCCCCGACCCACCTGGGGCCCATGGGCTTCATGCACCAGCACGCGCTGATGTCCCCCGGCATGCCCCACCCGGGGGTCCCCCGGCCCGACGGCTCCATCATCAAGGGGCAGCCCATGCATAATATGGAAGCTCTGGCGAGATCTGGTATTTGGGAGAATTGTAGAGCAGCGTACGAGGACATCGTAAAGCATCTCGAAAGATTACGCGACGAAAGAGGCGACATGGAACGAGTTATGGCTATGGATAAAGCACGCGAGGGATCACATAACGGTTCATCTCCCGGCCACAGTCCTGTCCTGAATCTCTCAAAATCCGGGTCAGGCGGCGACCGCGACCGGTCGGACCGGGGCGAGCGAGACCGCGGCGACCGAGGCGACGGGTCGGCCAGCGGGAGGAGCTCGGCCGCCTCCCGCCGCACCCCGCAACCCCCGCGGATGCCCTCCGCGGCGACGGCGGCGGCGTCGCCCCGGTCGCACAGCGACGAGAGCGACGCGGCCTTGTCCGACCAAGACGACCATAACGTCAAAGATGAGGACGAGGGAGCAGACTTGAGCGACGGGGAACGTGACTTGCAAGCATCGTCATCGCCGGCGCCGGTGAGCTACGCGGTCCCCACCGGGACCCCGCCTTCAGTGCCTGTAGACCCCACGGCTGACACCCTCGTCTCATCAACGGAAACCCTGCTGAGGAACATCCAGGGCCTCCTCAAGGTAGCGGCTGATAATGCAAGGCAGCAGGAGAGACAAATTAGTTACGAGAAAGCCGAACTGAAAATGGACGTTCTCAGAGAAAGGGAGGTGAAAGACAACTTGGAGCGGCAGTTGCTTGATGAGCAAAAAATGAGAGTGATGTATCAGAAACGATTAAAGAAAGAAAGAAAACAGCGGCAACAAATCCAGGATCAGCTGGAAATGGAGCTGAAGAGAAAACAGAAGATTGAAGAAGCGCTAAAGCAGTCTGGAGCGCCGGCTGAGATCCTCAGAATTGTCACTGAAAATCTTCAACCACCGGCGCCAGAGACTCCTCGTTCGGAGCGCGAGAACGGTTCCGAGAGCAAGCCGCCTAGTACGGAGCCGCCGGCGGCTTCGCCCCCCTTCCAGAGGGAGCCGCCGCGTACGCCGGACAAGCCGCAGTGGAACTACCCGCCGCCGCCCGTGGACATCATGAGCGGAGGAGCCGCCTTCTGGCAAAACTATTCTGAATCCCTGGCGCAGGAATTGGAGATGGAGCGCAAGTCGCGGCAGCAGGCGATGGAGCGCGACGTCAAGAGCCCGCTGTCGGACCGCGCCGGGTACTACAAGAACTCCGTGCTCTTCAGCTCGGCCACTTAG

>lim1-like 1

TTAGTCGTCGCGCGTGTAGGTGCGTTGTGCGGCGCGCGAGCCCGCGGTGTGTCTAGTGCAGTGCCGTGTAGAGGTGCCGAAGTGGCGGGTTTCCTTGCCTCAGGACGGGGGGCCCATGCCCCCTTCCACCAACACCACATTGGTAAAAGACGTGGCGAGGCAGGCGGCGCGATGGCGTGCGCCGGCTGCGAGAAGCCGATCCTGGACAAGTTCCTCCTCCACGTGCTGGAGCGCGCGTGGCACGCGGCGTGCGTCCGCTGCGCCGACTGCCGCGCGCCGCTGGCCGACAAGTGCTATTCTAGGGACAACAAATTGTTCTGTAGGAACGACTTTTTTAGGCGATACGGCACAAAATGTAGCGGCTGCGGGCACGGCATATCTCCCTCCGACCTGGTCCGCAAAGCGCGGGAGAAGGTGTTCCACCTCAACTGCTTCACCTGCCTAGTCTGCAGGAAGCAGCTGTCGACCGGCGAAGAACTGTACGTGCTAGACGACAACAAGTTTATCTGCAAGGAGGATTACTTGGCGGGGAAGGCGCCGACGCATTCGGACTCCCTCCTCGGCTCAGGGTCGGACGAAGAAGAAGAAGACGAGTCCCGCGCAGCGAACAACTCCAGTAGTCCTGCGCACGCGCCGCACCCAGCCTTACACTCGGACATCACGCATAATGGCGACGCCAAGCCGCATGAGGACTCGGAGGACCAGGGCTCGCTGGACGGAGACCCAGAGACCAGAGACTCGCAGGCCGAGAACAAGTCCCCTGATGATGGCAATGGAGGCTCCAAGCGGCGGGGACCGAGGACTACCATCAAGGCGAAGCAGCTGGAGATCCTGAAGTCGGCGTTCAGTCAGACGCCGAAGCCGACGAGGCATATACGCGAGCAGCTGGCCAAGGAAACCGGTCTGCCGATGAGGGTGATACAGGTCTGGTTCCAAAACAAGCGTTCGAAAGAGCGCCGCCTGAAGCAGCTCACCTCAATGGGGAGGGGTCCGTTCTTCGGGTCCTCCCGCAAGATGCGGGGGTTCCCCATGAACCTCTCCCCCGGGGGGTTAGAAGAGGGTCCTCCGGGGTTTCCGTACTTCGCGACGGCAGATGGCAAGTTCGAGTTTGGGTATGGCCCGCCGTTCCACCATGATGCGCCGTTCTTCCATCCACCGCCGGCTATGCCGTTCAACCAGCCAGGTCGTTTCGCAGGCGGAATGGAGTCGCTGCCAGGCGGCGAGTTCCCCGAGCAGTTCCCTCCGCCGGAGCACCTGGTCCTCCCGCGGCCATCCTCCCCCGAGTTCACCTTCGGAGACGCCCCGCCCCCCCTCCACCCCGAGGGTCTGGTCTGGTAG

>lim1-like 2

ATGGTACATCAAGCAAACAACTTTTTCTTCCCTCATTTTAGAATTTTCTTTGTTTTTCTTAATCCCAGGTTCAAAAGACGACGACTGTACCACGCTAGTATCCCGAAGTGTGGCGGGTGTCATGAGATGATAGTGGACCGGTACGTGCTGAAGGTGTCCGACCGGACGTGGCACGCGGGCTGCCTGCGATGCGTCGAGTGCCGCGCCATGCTGTCCGGGAAATGCTTCGCGAGGAATAACCAGCTGTACTGCACGGACGATTTCTTCAAGCGGTTCGGCACCAAGTGCGCGGGCTGCGGGCAGGGCATCCCTCCGACGCAGGTGGTGCGCCGCGCCCAATCCCACGTGTACCACCTACGCTGCTTCGCCTGTGCAGCCTGTGCTAGAACCCTCAATACTGGCGACGAGTTCTACCTGATGGAGGACGGGAAACTGGTCTGCAAGCCGGACTATGAAGCGGCGAGAGCGAAAGGAGGCGAGGGTTCCCTGGACGGGGACGCGGCTAGCAAGCGGCCCCGGACCACCATCACGGCGAAGCAACTGGAGACCCTGAAGAGTGCCTACAGCAGTAGTCCGAAGCCTGCGAGGCATGTCAGGGAACAGCTGGCCCATGATACAGGGCTCGACATGCGGGTGGTTCAAGTCTGGTTTCAAAATAGACGAGCGAAAGAAAAGCGTCTAAAGAAAGACGCCGGGCGAACGCGCTGGTCCCAGTACTTCAGGTCCATGAAGAGCGGCGGAGGATCTCCAAGACACGACCGATTGCTCGACAAAGACGAACTCAAGATTGACCTTGACTCTTTCAGTCATCATGAACTCAGCAACGATAGCTACAGTACGGTGGCTTTAGGCGGCGAGGAAGGCTCCCCGGCGGGCGGGGGCGGCGCGGGCGCTACGGGGGGCGCGCGCTATGCCGCTACGCCGCCCTACCTGCGCACGCATTCGCCACCGCACCCGCATTACCACTACCCTCCAGATCACCTCGTTTATACCAATATCGCCTCATTTTTGCCAGGCCAAGCGATGAGCGGCGCCGGCATCGGCGGAGCGGGAACGGGCGGAGCATCCGACCTTAGCAGTTCCTCATCTCCCGCTGCCGGGGGTTATCCTGACTTCCCACCGTCCCCCGACTCGTGGTTAGGCGAGCCTCACCATTACTCGCCTAGGGGATTCCCCTAG

>lim1-like 3

ATGAAGGTAATTCAACGGTTCCAGACGATAGCAATGACAAGTGATACTAATATGCAGTGCGAGAGGCGAACGCCGCAGCAAGGAAGCGGCGGTAGCCCCGGGCTAGGCTCGCATATGCTGGCCATGGACGTGACGAAGGAGGCGCGCGCGTCGCCGCTACCGGCGTCCGCGCAGCAGCCAGCTTCGGCTTCGGGACAGCAGCCCCCGCAGCCTCAGATCTGCGCCGGCTGCAGCAAGGTGATCACCGAGCGGTACCTCCTGAAGGCATTGGACCAGCTCTGGCACGAGGACTGCCTCAAATGCGGCTGCTGCGACTGCCGGCTCGGGGAGGTCGGGCACACCCTGTACACCCGCGCCAACCTGATCCTGTGCAAGCGGGACTACCTACGGTTGTTCGGCAACACCGGCTACTGCGCGGCCTGCAACAAGGTGATCCCCGCGTTCGAAATGGTGATGCGTGCGCGCAGCAACGTCTACCATCTGGAGTGTTTCGCGTGCCAGCAGTGTAACCATCGATTCTGCGTCGGCGACCGTTTCTACCTGTGCGAGAACAAGATCCTTTGCGAGTACGACTATGAGGAGCGCCTCGTGTTCGCCAACATGGCCTACAACCCGCCTCCGCTATCGCATCTCAAGCGGCAGACCACGCACCTCCCTCCTCCACCGACAAGCAGCGCGATGGGCGGCATGATCAACGGCTCGGGCCGCGCCGGCGACCTCAACAACAACATGTCAGGTGCTTCTCCCGCGCCCTTCGCCGCCCCCCCGCACCTCAAGCCCCTTGGCCTTTCTGCGTCCAGCTGA

>Wnt-like

GCCGCCGCCGCCGCCAATGTCCGAGTCTGGAAGTGGGGCGGCTGCTCAGACAATATCGGCTTCGGCTTCCACTTCAGCAGGGAATTCGTCGACACAGGAGAAAGAGGAAAAACCTTAAGAGAAAAAATGAATCTACACAACAACGAGGCCGGCAGAACGCACGTGCAGTCGGAGATGCGGCAGGAGTGCAAGTGCCACGGGATGTCAGGCTCGTGCACGGTGAAGACGTGCTGGATGCGGCTGCCGAGCTTCCGCTCCGTGGGCGACGCGCTCAAGGACCGCTTCGACGGCGCGTCGCGGGTCATGATGCCTAATGCGGAGCTGGAGGCGCCGGCGCAGCGCAACGACGCCGCCCCGCATCGGGTGCCGCGCCGCGACCGCTACCGCTTCCAGCTGCGGCCGCACAACCCCGACCACAAGTCGCCGGGCCTGAAGGACCTCGTGTACCTGGAGTCCTCGCCGGGGTTCTGCGAGAAGAACCCGCGGCTCGGCATCCCCGGCACGCACGGCCGCGCCTGCAACGACACCAGCATCGGCGTGGACGGCTGCGACCTCATGTGCTGCGGCCGCGGCTACCGGACTGAGACCATGTTCGTGGTGGAACGGTGCAACTGCACCTTCCACTGGTGTTGCGAGGTCAAATGCAAAGTTTGTCGCACGGAAAAAGTGGTTCACACGTGTTTATAG

>foxQ2-like

GCACGCTCCCGCCGCGACAACATGTGCAGCGGCGCCGACGGAGCCCCCTGGCCGCTCGGCAAGGACGCGCCGGCCGTCACCGCCGCCGCGCTCGACCACTACCGCCTGCAGCTCTACAACTATGCCGTGGCCGAGCGCCTGCGCCTCTACCCGCCCGGCGTGGCGCCCTGCTACGGGCCCTACGCGCCGCGCCTGGCGCTCTCCATGTCGCTGCTGCAGCAGCGCGTGCTGCAGCCCGAGGAGCCCAAGCCGCAGCACAGCTACATCGGCCTCATCGCCATGGCCATCCTCAGCTCGCCGGAGCGCAAGCTCGTGCTCTCCGACATCTACCAGCACATCCTCGACAACTACCCGTACTTCCGCACCCGCGGGCCCGGCTGGCGGAACTCCATCCGGCACAACCTGTCGCTGAACGACTGCTTCGTGAAGGCCGGCCGGTCGGCCAACGGCAAAGGCCACTACTGGGCGATCCACCCGGCCAACATCGAAGATTTCCGAAAGGGAGACTTCAGGAGACGCAAAGCGCAGAGAAAAGTGAGGAAGCACATGGGTCTGGCGGTGGACGACGACGGCGAGGACTCACCATCGCCGCCGCCGCAGTCGCCCCCGCCGACGGCTCTGCCGCTGCCGTTCTGGGGCGGCGGGCGCCTGCCGGGCGGCGCTCAGGCGCGGAAGAGGCAGTTCGACGTGGCATCGCTGCTGGCGCCGGACGACGCGCCCGAGAAGCAGCGGCAGCGGCGCGACAGCAGCGGCGAGGAGGAGCCGGAGGAGGACATCGACGTGGTGGCGAGCGACGCGGAGGAGCGGCCGGGCGAGGAGGAGGCGCGCGCGCCGCTGGCGCCGGCGGCGCAGTACCCGCTGCTGGGCGGCTGGTGGCCGGCGCTGGACCCGGCGCTGCTGCACCAGCTGCGCCGCCACTCCGCCGCCGCCGCCTCGCCGCCCAGCCCCGACCGCCAGCGCCCGCCCGACACCTAG

>Dorsal-like ATGGACGTCGGCGGAGGCGTGGCCCCCGATGCGGTCGTAGTGCATAATGCCGGCGACCAGAGCGCCCACCTGAGTGATGTTTTTGAGGTGATCTCGCAGGCGGACCCGTCGTTCGGCGCGGGCGCAGCGGGCGACGCGCACGGCATCATGGCGCAGGCCCCGGCGCCCTACGCGTACATCATCGAGCAACCCGCGCCCAAGTCGCTCAGGTTTCGCTATGAGTGCGAAGGGCGGTCAGCGGGTTCGATTCCCGGCGTGAACAGCACGCAAGAGAACAAGACGTTCCCCACGATCAAGGTCTGCGGCTACAAGGGCTGCATGGTCATCGTGGTGTCATGCGTCACCAAGGAGGAGCCCTACAAGCCGCACCCCCACAACCTGGTGGGGCGCGACTGCCAAATGGGCGTGTGCACGGTGAAGGTGCGCACCGAGGGCGATGCTGACGCCTGCCAGGTGCAGTTCAAGAACCTCGGCATCCAGTGCGTCAAGCGCCGCGACATCGCCGACGCGCTGCGCACGCGCCAGGAGCTCCGCGTCAACCCCTTCAAGACGGGTTACGCGCACCGCAGCCAGCCGCAGAGCATCGACCTGAACGCGGTGCGGCTGTGCTTCCAGGTGTTCCTCACAGACGACGCATACAAGGTGCAGCGTGCGCTGCCGCCCGTCGTCTCTGACGTCATCTACGACAAGAAGGCCATGTCCGACCTGCTTATAGTGCGCTCCAGCCATTGTTCTGGAAGCGTCAAGGGCGGGACCACCGTCATTTTGCTGTGCGAGAAGGTGACGCGTGAGGACACAGAAGTGTGGTTCTTCTTAGAAGAGAACGGCGCCGTGGTGTGGTCGGAGCGTGGACACATTGACTACGTGCACAAGCAGCTTGCTATAGTACTCAAGACGCCGCCTTATCGCGACCAGCGTTTGGACCACCACGTTACCGTCCAGTTCCAGCTGCAGCGGCTGTCGGACGGCGCCAAGAGCAACGCCTACAGCTTCGAGTACATCCCCAACGTGCAAGGTACCCGCAAGCCGCTGCCCGACCTCGCCTCCCTCGCGCTACTGCTCGCAGACACTAACCCCGCCCCAAATCCCGCCCCTAACCCCGCCCCCAACCCTACAGACACAAACAACAACAACCGCGACAAAGACGCGCTCGTCAGCTCCACCAGCGACTCTTATTCTGACCATACCACGCCCCTCGACACAAACGGCGACGCCTCAGAAGCGCTCCTCCCCGATTCCAACGAAAAGAGCCTCGACGATCTCCTCGATCAAGTCGCGGAGCTCGACGAGATTTACACCGAGAATCGCACGCGGTTAGAAAACATCACGTCGATGCAAACAGACGACGACATGGAGGATTTCAACGACGCTGGAACCTACACTAGTCTCCAGCTCGCCTTTAAAAATCCCATCACTATAACTGAGCCGGAATCGCTCCCGTACGAGGATGTCCAAGTCCACAGCTACCGCGGCCCGATCATAGAGTTCGCGCCGCTCAAACGCGACACCGACGATGACAATAAAGCCCCTCCGTTGCCACCGAAACGCGTCCGCAAACCCTCGACGGAACAGTTCAAGACGAGCCAGACCTCAGTCGATAGCATCCTCAAGCCGGGACGCCAGATCCCAGTTACCAGGAACCCTGAGGTCCTGCGGCGTGATCGCACTGAGTTGTCTGTAGCACGCAGTGAGCCGGCTTTGCCTCCGGTCAAGAAGCGGTCGTTCTTCTCGCGCCTATTCAGGCGAAAAGAGAAGTCTCCCGCTCCGAGCGTTAAATCGGAAGGCAGAAAGGAGAAGAAGTCCAAACCGGTCGGTCGCTCCGTCAGTAGCGTGTCAGGGCTGCGACCCTCGAAGTTCAAATCCTCAGTATCGCATACGTCTCTGAAAGACAACGCGTCGGGCGCCGGTCTAAGCTACGCGGACAGTATCACGCATATCTCCCTACACGGTGACGGTGAAGACGACCGCTCGCAGGCGTCTCTCCGCCGTCCTGCCAGCCTGGCGCCTCTACCGCCGACAGATGGCGGCACCATCTTGGTAGCGGAAAGCGTCCTCGCATTAGACGCGTCGGCCTTCAAAAAGCTCCAAGACGATCTCGATTTAACCGACGCTGAACATTACGCCCTGTATATGGCTGTAGCGCCGCACGCGACGGTATCGGAGTTTGACGAGACCAGTTGCTACTACTCGCCGGTAGATGGCAGCAAATTTCAGAATCAAAATTAA

>runt-like

ATGCACCTTCCGCACGCGAGCCCCCCGGCCCCCAGTATGGCTGACGTGTATTCCCACATCCACGAGTACTACAGGCAGAGCCACGGCGAGCTGGTGCAGACTGGGTCGCCGGCGGTGCTGTGCTCTGCGCTGCCTGGACACTGGCGCTCGAACAAGTCCCTGCCAGTGGCTTTCAAAGTGGTGGCTCTGGATGATGTGCAGGACGGCACATTAGTGACGATCAAGGCAGGGAATGACGAGAATGTGATGGCGGAGCTGAGGAACTGCACTGCTGTGATGAAGAACCAAGTGGCCAAGTTCAATGACTTGAGGTTCGTCGGTCGCAGTGGTCGCGGAAAGTCTTTTTCGCTGACGATCACCATCAGCACATTCCCCAGCCAAGTCGCTACGTACATCAAAGCCATCAAGGTTACAGTTGATGGACCCAGAGAACCCAGAACCAAACAAAATTACGGCTACGGACATCCTGGTGCCTTCAGCCCGTTCCTGCTAAACCCTGGATGGCTGGACGCCGCTTACCTGAACTACGCCTGGGCGGATTACTTCAGGCCGCCACAGATGAGGGACTCCACTGCAGCACTAGTTAAAGGAGCGCCACCGCTAGCGACGCCGCCGGTGCTGCCTGGAGCGGAGCTATTCCCATTCCCACCAGCGATGGCGAATATTCCTCCTGGTGGGTTAATACCTCCACCAGGCGCATTCCTGCCTACGAATGGATTATTGCCATTCCCACCGCATCCAGCTGAGTTGGCACTCAAAGGGATACCGCCTGAACTATCACTCAAGAACGGCGTGAACCCGTACGAAGCTTTGAGGCATTTGCAAAGCAACGTATCTTCAATGGACACATCGAGCGCTCATTTGTCGCCGACTAGCAGTAGGCAGAGCAGTCCACGGAGCATGGCGAACGCGAGTCCAGACCGATCGAAAGCCGATTCGAAATCAGAAGTCAATTCGATGCACGAAGCGACGATTTCTGATGAGTCTGATGAGGAGCCCATCGAGGTTGTGAAGTCTGCGTTCCATCCCACGAGACCAGCTAATGTGGAACTACAAGAGATGAAGCGAGTGCAGGCTGCTGATTCGACGGTCTCAGACCGGCCGCGGATAAAGAATGAATTGAAAGCTCCTTCTACGAGAACCACTCGAGTCCTTTCAACTAGTCCAACGTCAACTAAAATTAACAATGGTGCGATAGCTGGGCAAAAAAACTGTGTGGCGGCCATATTGACATTCAAACTTTCTAGTGTTAGTGTGAAATAA

>peptidyl alpha-hydroxylating monooxygenase-like

ATGAATTTCTTGGGTTTACCCATCCTTGTGTGTGCATATTTTAGTTATGTGTACGCCTACGAAGTCGATACATATGATTTTCTCATGCCAAATGTTTGGCCTCACAGAGATGAGCTTTACCTCTGCACCCCAATCAGGATCTCTCCTCGCAACAACTATTACATAGTGGGATTCGAACCTAATGCTACTATGCACACTGCTCACCACATGCTGTTGTATGGATGCTCGGAGCCTGGAACTGACGATCCTGTTTGGAGCTGTGGAGAAATGCAGAGTAACGACATTGACGACCAGTACAACACGGCCAGCCCCTGCCGGGAGGGATCTCAGATCGTGTACGCTTGGGCCCGCGACGCTCCGAGCCTGCAGCTGCCCAAAGACGTCGGCTTCCTGATCGGAAAGGACTCGCAGATCAAATACCTGGTGCTGCAGATACATTACATGCACAAATTCCCAGAGAGCAAAAGGGACAATTCCGGAGTGTTCATCAAATACACCAAAGAACCCATGCATCGCCAAGCGGGCGTGATTCTTCTAGGCACTGGCGGCGTTATACCAGCTAATAGGGTGGAACATATGGAGACGGCTTGCACCATCCGTGAGAACAAGGTCATTCACCCCTTCGCTTTCCGCACGCACACTCATGCGCTTGGCACAGCAGTATCAGGATACGCCGTCCGCAGAGAGGCGACAGGGGACATTTGGCAACTAATCGGCAAGAAGAACCCCCAACTGCCCCAGATGTTTTACCCCGTGGCCGACAAGACCCCCATAAAGCAAGGCGACGTGCTAGCCGCTAGGTGCACCATGAACAATACGCACTCATTCCCTGTTAAGATTGGCGCAACAAACAAAGACGAGATGTGCAACTTCTACCTAATGTACTGGGTGGAGAACACCTACCCGCTCGACCAGAAGTACTGTTTCAGCGCCGGGCCCCCCTACTATTACTGGGTGCGCGCGCCGCAGAACTTCAACCGCGTGCCGGACCTCGAAGCCAGCTCCTTGTAA

>ci-like

TCTCTCTCCCACGCGTCGCTCGCGCAGCAACTCCTCGCGCGAGGGGGTGTGGTGGGGGGCAGCGGGGTGCTAGCGGGAGGGGTGTTGATGGACCCCGCGCATCAGCAAGCGGCGGCGGCGGCGGCGCACCATGCGGCGCACGCGCATCTGGTCGCTGGTATTCATAGATCTCACATCTCATCGCCGACGCAGCTGCTCATAGGCGCGCCAGTTGACGTCAGACCAGGCCTGGGGTTAGACGGCACCCCTCCACAACATATGCAGCAACCACCCCAGCAGCCTGAGGTCACCAGCATCATGGAAGCTGACAGTGCGTCAACGATAACACAACGAAAGTCACCCCAGAGCCTGATGTCTCATCGAGACAATATGCACAACAACAAGTCGTTATCAGCGGCTGCCGAGAGCACTGTCCACGACGGTCTGGACTCCAAGGATGAGCCTGGAGACTTTATTGAGACCAACTGTCATTGGGTGGACTGCAAACTTGAGTTCCCAACTCAGGATGACCTGGTAAAACACATCAACACGGACCACATCCACGCCAGCAAGAAGGCCTTTGTATGCCGATGGGTAGGCTGTTCCAGAGATGAGAAACCCTTCAAGGCTCAATACATGCTGGTGGTACATATGAGGAGGCACACGGGGGAGAAACCTCATAAGTGTACTTTCGAGGGTTGTTGCAAAGCCTACTCCCGATTGGAAAACCTAAAGACCCATCTCCGAAGTCATACGGGCGAGAAACCTTACACCTGCGAATACCCTGGATGCGCCAAGGCCTTCTCCAACGCTAGTGATCGAGCTAAGCACCAGAATCGGACGCATAGTAATGAGAAACCATACGTGTGCAAAGCGCCTGGTTGTACCAAGAGGTACACTGACCCTTCATCCCTTCGGAAACACGTGAAAACCGTCCATGGTGCTGAGTTCTATGCCAGTAAGAAGCACAAAGGTTGCAGCCGTGGAGACGACTCCGCAGAATCAGGGGGCGGGGGGGCTGGCTCCTCCCCGCGGTCGGAAGAGGGGGGCATGCCCCCCGGCGCGAGGGGGCACACGTCATCCGCATCGGTCAAGAGCGAGAGCCCCGCTTCACCGCTGCCTCTGGCGTTGCACACTCCTGCGCATCAGCTTTCAGCTCAATGTGGTGGAGAACTGGACTTTGGCGGTTCTGGTCTCGGTGGATTTAGTGATGAAAATGGCGCCCCTTACTTCAGATTAGATGGAGAGGTGGAACAAGAAGTAGTGGGTGAAGTGGGCCAACTACCGCTGATGTTGCGAGCGATGGTGGCGATAGGGGAGCCGCGGACGCACCACCACACCCCCCGGTTTGGGAACAACAAGATGGGGGTGGGGAGGCTGATGCCGCCTGCGCATGTCGGCGATATTGGCGGGGGTGGAGTGCCAGGCCGGACGGAATTAGGCAACACTAACGTAGCGGTCGAACTTAAAACAGGCCTCCCAAATACAAGGAGAGACTCGGGAATTTCTTCCGGGAGCAGCCTTTACAGTGCCAGGTCGTCTGACATCTCCCGCAAGAGCAGCCAAGCATCGGTAGTGTCGGGCGCGGTGGCGGCGCCGCAACGCGTCGTGGCGCAACACAACGCGGTCTACGACCAGCTCTCGCCTGACAGCAGTCGAAGATCTAGTCAAGTGTCGTGCGTGGGGTATGCGCCGGCGCCGTCGTCCGCGCTCGCCGCCGTCCAGGCTGTCAGGACCTCTCAAGGGAACCAGGCTGTCTTGCTCCGCGGCGTGACCTGCTCCGAAGTGAGGGCCGAGGAGTTGGCTCTAGAATTGGATCCCAACGTGCAGGTGAAGGAAGAAGCCAGGAGGCTCTCGGAGCAGTCCAACCTCAGCGACCCGGCGCAGGCCTACCAGCCCTACCCGTGTGGAGCTGATGATGTGGGCGACGCCGCGTTCCCGTTCAAAACTGAAGACGACCACGACACCGTCACGTACAAGGACTCCCGGTCCAACTCCACCGCCACCGTGGTCATCACCACCGCCCAGGTCCACCATCCCAACCAAGAAGTTAACTTGGAACAGGTTGCAGAAGGTGAAATGGTGGAGAACAAGCTGGTGATCCCAGACGAAATGATGCAATATCTTAACCAATCAATATTGGCAACCGAATCGGCGGCACCAGCTCAGACCGACTCCACCAAAGCGCCTGAAGAAACCAGTCAGGATAGCGATGCCACCAAAGACAATGACGCCCAAAACGCGAACAACACCAGTGACAAAATCAGTGACGTAGCCACCAGTGACGATTCTCTACTCAAAAACCTAGGTGCCATAGGAAGTGATCTCAATATCAGTGATATCCCAGTCGATTTAAGGTCTTTAGACGTTAGCATGTCGGGTAACAGCGGCTCTCTCCTAGCTACAAAGTCCCCGGATGACAAGAACCCCCTTCAAGAGCAAATCATACCTGAAGTACCAAATGAGAAGTGTGAACAGGAATACACCAAACCCGTGACCAGTCACATCAACACGAATAATCCGTTACAGTCACTGCAGACGATGGCGGCGAATCAGACGGAGCAGTCGAACCGTATGAGGATGAACGCTCTACCTCAAAAACAAACTTCAATAAGTCCAAAGACTATGGTGACCACCCCTCAAAATGTGCTGAGTCCACAGAACATAGCTCATAGCATGTTGAGTCCGCAAAGTCTGCCGCATAGTGCGATGAGTCCTCAAAGTGTTATGAGCCCTCATCATATTCCTCACAACGTCATGAGCCCACCGAGCGTGTATAATGTCATGAGTCCTCAAAGCGTCATGAGTGTCATGTCTCCTCAACACAATGCTATGAGCCCCCAAAGTATGCAGAGCATGATGAGCCCTCAAATGTCTAACCAAATGATGATGAGTCCACGCCACAATAACATGGGCAGCCCTATGTCCCAGAACATGGGAGGACAGATGGTCAATATGGCAAGTCCGAGGGCGCAAAACATAGCAAGTCCTATGAGCCATGGCATGCCGAGCCCGATGCATCCAGGACTGCAGAGCCCCATGTCACAAGGAGTACCCAGTCCCATGGTACAAAACATGTCGAATATGACGATGAATTCTCCGGGGCAAAACCAGAATCAAAACGTAATGATGAACATGAACCAACAGCAGCAAATGCAGCAGATACCAGGAAACTACATGAATAATCGTCAAAATTGCAACCCCAAGATGCCCAATAGAACTAATGTCCCCAATCAGTATCAGAACCACAATTACAATCAAGCCCCGCCATACCCGATGCAGAATCAACAAAATATGAACAGGAATCAGAATATGCAACAGTATCCGATGATGCAGCAGTATAATCAAAACCAAATGATGCATCAGATGCCACCGAATCAGCCAAATATGGTTTACAATAACCAGATGATGAATTATCAGCAACCTATGAACTATCCAAACCAAAGCAACCAGATGCATCCCATGCAAATGTCGAGGTCCTCGATGATGAGTGTGGACAACAGCGGGAACATGAGTCGAGGAGCGATGAACAGTTACTGTGAACAGCAGAATCCTCAGTACAACCAAAACATGCAGTACCCTCAACCGCCTCCTTACAATTCTGTCGTAAACGCTGCAAATGTCATGGGCCCTCCACCTCCTAAAAATAATCACCAATACAACCAAGCGATGATGAACAACAACCAATATTACAATCACCAGCGGCCGTATAATCAGTGGGACTATCCTGGTAATCAGTTCAACAAGCACAACGTGCAGAAATCAGTTCAGAACTCTGTGAACATGTCGACTGGAAGTCAAAAGCCTAACGGGGTCAGAGCATCGATGAATTGCAACCAGATGATCAAGAATATGGGGGAGCAGCAGCAGACTGATTGTAGTATGAACAGTTTGAGGAGCCAAAACAATCAGCCCAGCGATGTCCAGGTCTGGGACATCTCCCAGTCGCAGATTGAAGCTAATAACGGTAGGAAGAAGAACCAAAATGGCATGAGACAAGAAACCTACCAACGGACTTTGGAGTACGTGGAAAGTTGTGAGAACTGGAAGAGTTCAGAAATGGTTTCCAGCAGCACCCACCCTTTACAAGGTGGCGACAACATGGTTGTGAACGATCTCCAGACATCGTTGTCTTCGTTTTATGAAGAAAACCAATACCTCCAAATGATTCAATAG

>sim-like

ATGAAGGAGAAGAGCAAGAACGCAGCGCGCTCCAGGAGAGAGAAGGAGAACGCCGAGTTCCTGGAGCTGGCCAAGCTGCTGCCGCTGCCGGCCGCCATCACCTCGCAGCTGGACAAGGCGTCCGTCATCCGGCTTACTACCAGCTATCTCAAAATGCGACAGGTCTTCCCTGACGGTCTAGGCGACGCGTGGGGCGCGGCGCCCCCGCCCCCTCAGCCCCGGGAACTGTCCATCAGGGAGCTGGGTTCCCACCTGCTCCAGACCCTGGACGGGTTCATCTTCGTGGTGGCGCCGGATGGGAAGATCATGTACATCAGTGAGACGGCATCGGTGCATCTGGGGTTGAGTCAGGTGGAGCTGACCGGCAACTCGATCTACGAGTACATCCACCAGGCCGACCACGAGGAGATGAGCGCCGTTCTCAGTCTGCAGCACCCACATTCTTACTTGGGCCATCAGCACGCCGGCCTTGGATACCCAGTGGGGGGCACGTGGGGCCCGACGGTGGACATCGAGTGCGAGAGAGCCTTCTTCATCAGGATGAAGTGTGTCCTCGCCAAGAGAAACGCAGGTCTCACCACCGCAGGGTACAAGGTGATCCACTGCTCGGGGTACCTCCGAGCGCGCCGCTTCGGCGAGGGCTCGGCGCCTTTGGGACTGGTGGCAGTAGGACACTCGCTGCCGCCCTCCGCCGTGACAGAACTGAAGCTGCACTCCAACATGTTCATGTTCAGGGCTTCCCTGGACATGCGGCTTATATTCCTGGACGCTAGAGTGGCGTCTCTCACGGGGTACGAACCGCAGGATCTGATCGAGAAGACCCTGTACCACTACATCCACGGGACCGACGTGCTTCACATGCGATACTCTCACTGCACTTTGCTGACCAAAGGGCAGGTGACGTCGCGCTACTACCGCTTCCTGACCAAGTCTGGCGGCTGGGTGTGGATGCAGAGTTACGCCACCATCGTCCATAATTCACGGTCTTCGCGCCCACACTGTATTGTGTCCGTCAACTATGTGCTCAGCGACATCGAAGAGAAGCACCTAATATTAAACATCGACCAAGGCCCGCCGAAGCTGAACCACGAAGTCCCCGCGCCGTCCCACGTCCCCACCACCACTACGTCCCCACAAATGCCCCACATACGACAGACTGACAAACTCAACCACGTCACGTTGAACGAAAGCGATTTCAGCGGCGATTCTTCTAGTGGTTATACATACCCAGAGTATGCGCTCCCAGTCATCCCAACGTACGAATCGCACGAAGAATTCAACCAACAAAACGGTTCCTACCAAGAACTCTTCTACGAAAATTATCCAGACCCAGAGATATTGCCAAACAACTACGTAAACTATCCTCAAAATCAACGACCGTATTCCGCTAGCTCGTCATCCTGTAGCTCAATCGAGAGCTCAGAGATCGCGAATCAGCAGTACAGCTATACGAATCTAATATCGTTCTACGGGCATAACGGCCAGAATCAAAATGGCCGCCCGATTGACGGCAATTTTGGCGGGAATTTCGGCAAGATGACGCCGAGCCCGACGGTGCAAGAAGGTGCGTACACGAGCGTGATAGTTGACAACACGCAGCAGTTCCACCACCACGGCGGTGGGGTCAACGAGTTTGTGCATTGA

>sox1/2/3-like 1

ATGAACGCGTTCATGGTCTGGTCCCGGCTGCAGAGGAGGAAGATTGCCCAGGACAACCCAAAGATGCACAATTCTGAGATCTCGAAGAGATTAGGTGCAGAATGGAAGCTGCTCACAGAAGATGAGAAGCGGCCGTTCATTGACGAGGCTAAGCGGCTCCGAGCCATGCACATGAAGGAGCACCCCGACTACAAGTACCGGCCTCGGAGGAAGCCCAAGACCTTGAGGAAGGAGGGGTACCCGTACTCGATACCGTACCCCAGTGTACCCATGGACGCGCTCAGAGCTGGCATGGCAGGAGGTGGCATGGGGCAAGCCATGGGCGGCTACTACGGCGCGGCTTACGGGCCGCTTGGAGCCAGCATGGCCGCCGCCGCCGCTGCAGCTGCCCAGCAGAACGCCATCTCTGCTGCGCTGACGCCTAACGCTCAGGTTGGCTCATCAATGGACATGTCCAAATACTCGATCGAGGCGGAGAAGTACAGGAGCTATGGCATGTACGACCCCTCCCGAGGGTACCTGGACTCAGCAGCGCTGTCCAAGGCGTACATGTACATGGACCAGCAGCAGCAGCGCTCCTACCCCATGGACATCAGCAAGATGTACTCCGAAGCCTCCGCCGCAGCCATGGCCGGACTCAGCTCAGCCGTCAACTCCGCACCATCCAGTCTCTCGCCACGATCACCGGCTGAGTCACCAGACATGACTACAAAGACCCAAGACAGGCCTGAAGGCAGCTCCTCTTCAGGATCAAACCCCTCGCCTTCTCTCCCGTACTACCAGTCTTCGCCAAGCATCTTAATGCCTCAGTACCCAGGACAGTATGCGCAAGCCGCGCAAGGAGGATCAGAATTCAGGAGGCCGCTAACGGTCATATTCTGA

>sox1/2/3-like 2

TGGTCACGGGGTCAGCGCCGCAAGATGGCCCAGGACAACCCCAAGATGCACAATTCAGAGATTTCGAAGCGGCTAGGCGCCGAGTGGAAGCTGCTCACCGAGATGGAAAAGCGGCCGTTCATTGACGAGGCAAAGAGGCTGAGAGCACTACACATGAAAGAACACCCCGACTACAAATACCGACCAAGGCGGAAACCCAAGGCCTTGATCAAGAAAGAACCAAAGTTCGGCTTCGGCATCAGCGGCCTCATGGCCCCCGTGCCCCGCTTAGTGACCCCCTCGGTGCCCCACCCAGTGCCCCCCTTGTCGATGCCCCACCACTTGCTGCCGGACAAGCCTGAGCTGGGCCGAGCGCTGTTCCCACCACTACCGTACCCGTTCTACCCATTTGCGAAGATACCAGCTGACGATGGCAAGCTAGCGGCGGAGTTGGCGCATTTACAGATGTTCTCCTACCAGGCCCTGTACGGCGGCGCGCTCTACAGCAGCGCTCTCTACAACAGCGCCTTATCGCCCTGCGGATGTCCTCCCAGAAGGACACCGTCGCCTCCTCCCGCTGACGTCAAGCGACCCGTGGCCTACGTCCTCATGAAGGACGAGGAACCACCCCAGCACGTCATATGA

>cAMP-dependent protein kinase catalytic subunit-like

ATGATGATGGCCAGTGCGAAAATTATGAGGGAGTGCAGCTCGGAACTGAGTTCGGACGTGCGCTCGCTGTCCGTGGCGTCCTCCACGGAAGAGCAGGAGTCCACCACCACGGAGTCGGAGTACACGGAGGAGGAGAAAGAATACGAGCTGGACGATCTGCAAATACTCAAAACTATTGGCACTGGCACATTCGGCAGAGTTTGCCTGTGCCGTGACAAGGCGGCGGACGAGTACCTGGCGATGAAAATCCTGTCCATGGCAGACGTCATCCGTTTGAAGCAAGTTGATCACGTCATGAACGAGAAGAGCATCCTGGCCGAAATCAATCACCCATTCATAGTTAATTTACGATGGTGGACACACGACGACTCGTGCATCTACATGCTGTTCGACTACGTGTGCGGTGGAGAGCTGTTCTCCTACCTCAGGAACGCGGGCCGGTTCAGCAACAGTATTGGTAATTTCTACGCGGCAGAAATAGTATCGGCGCTAGAGTACCTCCACGCCCGGAATATCGTGTACCGAGACCTGAAGCCGGAAAACCTACTCCTAGCCAAGGACGGGCACCTGAAGATCACGGACTTCGGCTTCGCCAAGAAGCTCTCGGACCGAACCTGGACGTTATGCGGGACGCCCGAGTACCTGGCGCCAGAAATCATCCAAAGCAAAGGACACAACAAGGCGGTGGACTGGTGGGCTCTAGGAGTGCTCATCTACGAAATGTTGGTTGGGTATCCGCCATTCTACGACGACAACCCTTTTGGTATATACGAGAAGATCCTCAACGGCAGAGTGGAATGGCCGCGTCACTTGGATCCCGTTGCTAAGGACATCATCAAGAAACTTTTGGTTCAAGACAGGACGAAACGATTAGGAAATATGAAGTGCGGATCCGAGGACGTGAAACGGCACAGATGGTTCAAACATATAGACTGGGCGGATGTCTTCATGAAAAAATTACAGCCGCCGATCATTCCATCTGTATCGTATGAGGGCGATACGTCGAATTTCGACGAGTACCCCGAGACCGACTGGAAGGCGGTGCGCTCGCTGGACCCTGACGAACTCAAGTTGTTCGCCAACTTCTGA

>Fez1-like

ATGGGTCAGACAGAGGAGGTTCGGCCCAGCCCGCGGCGCCGGTCGCCGGACCTGCCGCCGCTGCCGCCGCCTATACAGCCTTGCAGCGGCTTCCTCGGGAATGGCAAATCCGTGATCCGTCCCATAGCGTTCAAGCCGCTCGGCGGGCGGCTGTCGGCGGGCGGCGAGCGGTACGGCTCCACGCCCGTGCTGGCCAGCAGGCCGCCCTCACACATGACTCTCTATGGCAGTTCATCAGACGTGCGGGAGGCGAGGTGGTGCGGCTCGCCGCAGCCCGCGTCGCTGTCCGCGCTGCCGCCCGCTTCCCTCTCCACTCCTTTGCACCAGCGGCATCACTCTGCCATAGTTATGAAGTCGAACTCGGTGGTGAGCGGCGACAAGGAGCCGGCGCCGGCGCCGTCGCCCGCTGACTCCGGCGTCGCTGAGCTTGAGAGAGACCACCACGACTACGGCACCACCGACCGCCTAAGAAGCCACGAGGTGCGCGTGCGAGCCCCGCGCGGGCTCATGCTGCAGAGCTACGCGCCTCGCTCGCGGGACGAGCGGGACAACGAGCTAGCGAGGGTTAGGCGGGACAGGGCGCTGCTGAGGCAGAGGCTAGAGGATACCGAGTGGAGCCTGTGCCAACGAGCTGGCGAGATAGCTCTGTTGAAGACGCAACTGAAAGACGCACAGAACGAACAAACAGCGAAAGGCCACGAATATCTCACTCTCAAAGCGGACTGTCGTCAATTGAGAGAGGCGCTCGACAAAAAAGAAAAAGAAATAATGAGACTCCGAGCGGAATCAGAGGAGAAGGAAAAAACGGCCAACCGGCTGCAAGCAGAAGTTGACCGACTAACCAACGACCTGATGGAAAGCGTCTCAGATCTAACCAAGACTAAAGAATCGAGCAAAAACGAAATCGAGCGGCTAAAAACGGAACTGAAAGAGCTCAGACAAGAACTATCCGACGTTTCCCTCAGCGGCTACGAAGGGATCGAGTGCGGGCGAACGATGAGAGGCATATACGACGTGTGCAAAACTAACGAGTACCACTGGAATTCTAATGACAGCGCATTAGAAGACGCCGAAGTCCAAAGACTGAATGGGGAGATGCCCGACCCTTGCGGGAGCCCGTGCCCCGCCAGCGTGTCCCTCGACCGACTCAAATGCGAGATGGAAGCAAAAGAAGCACAGTTCAACAACGAAAGGAGAAAGTGGTCGGAGGAGAAGGACAAAGTGCTCAGATACCAGAAACAGTTACAACTCAACTACGTCCAAATGTTCAAGAGATCGCGCACTTTAGAAGCAGAAGTGGACGGATTGAGGCTAGAACTGGAATTGTGCAACAAAAACATGAAGAACATCAACAAACACGGCAAAACTATTGAGTTGTAA

>hedgehog precursor-like

ATGAGGCTGTCGCTGGTGCTTGTGTGGCTGGGCGCCGCGGCGGCGTGCGGCCCCGGCCGCGGGTTCAACCCCCGCCAGCAGGCGCGGCGCTCCTACCCCCTCGTGTTCCGCCAGCACGAGCCCAACTACAGCGAGATCAACAAGTCCGCCAGCGGGCCCGCGGAGGGCCGCATCACCAGACATGACGAAAAGTTCAAAGACTTAGTGCCTAATTACAATCCGGACATCGATTTCAAAGACGACGAAGGCACCGGCGCCGATCGCCTCATGACCCAGAGATGCAAGGAGAAGCTGAATACGTTGGCGATCAGCGTCATGAACCAGTGGCCCGGCGTGCACTTGCGGGTCAACGAAGGCTGGGACGAAGAGAACTCTCACGGCGAGAATTCGCTGCACTACGAGGGGCGGGCGGTGGACATCACCACCAGCGACCGAGACACCAGCAAGATCGGCATGCTGGCGCGACTCGCCTACCAAGCTGGCTTCGACTGGGTCTACTACAGAAGCCGGTCGTACATCCACTGCTCGGTCAAGACAGAATCTTCTGTGGGCACTGGCGCTGGGTGCTTCCCGTCGGGCGCGGTGGTGCACACCGAGAGCGGGCCCATAGATATCGCGGCTCTACGCAAGGGTGACCGAGTGCTTGCTGCCGATGATGACGGCAAGATGATCTACTCCGAGGTATTAACCTTCATCGATCGAGATCCGAACGTCACTCGAGAGTACATAGAAATTACGGCCGAGAACGGAGTGGCCATCACGACCACTGCCTCTCACCTTCTGCTGCTGGCTTCCGCAGAGGGCTGGCGAGAGTCTTTCGCCGCCAGCGTGGATGTAGGAGACGTGCTCCTTACTCGGGGCCGAGGCAGCGTCATGAGGCCATCCCGCGTCGTGCACACGCGTGTCCTCACGAAGCAGGGAGTGTACGCTCCCATGACCAAAGCCGGCACCATCATCGTCGACGACGCCCTTGCCTCCTGCTACGCGCTCGTCAAGAGCCATTCCTTAGCCCACGCCGCCATGGCGCCTCTCCGGTGGATGGCCACGTGGAGCGGCTCCAACGATCTCCCCCGAGGCGTCCACTGGTACGCCAACGCTCTATATTCGTTTAGCGAATTCGTGCTACCCACGTCGTACAAATACCCATAA>APQ4-like-1ATGAAAACGGATTACGCTATGGATGAGATGCAGAAGAAGACAGGTTCGATCCTGGGCCTCTCAGACATCACAGACAACAAGCTGCTGTGGCGACAGTTGGTAGCAGAACTGCTGGGCACCTTCGTGCTGGTCTCCATCGGGGTGGCGTCCTGCATCGCCGTCGTCGAGGCCAACGCCCCGAAGGTCACCACCATCGCGCTTGCTTTCGGTCTGCTCGTCGGATCCATTGTGCAGGCGATCGGTCACGTTTCCGGCGGGCACATCAACCCGGCGGTGACGGCGGGGCTCTTCGCGTCCGGCGACATCAAGCTGCTGAAGGCTGTCCTCTACATCATCGTCCAGACCCTGGGAGCTGTTGCCGGTGCTGCCTTCATCAGGTTGGCGATACCTGACAGCCGAGTGGAGAGTGCAGGATTGGGACTGACGCTTCCCGGGCCCGGCGTGTCTGATGCTCAGGCTCTGCTGATCGAGGCTCTTATCACCTTCTTGCTGGTCATGGTGGTGCAAGGTGTCTGCGACGGTCGCCGCACCGACCTCAAGGGCTCGGCGCCTCTTGCCATCGGCCTCAGCATCACTGCTTGCCATGCTGCTTGCGTTCCGTTCTCGGGCGCCAGTATGAACCCCGCGCGTACCTTCGGCCCGGCCCTTGTCATGGGCGAATGGACAGCTCAATGGGTGTACTGGGTAGGCCCGCTAGTGGGAGGCGTGGTCGCCGGCTTCGTCTACAGGTTCATCTTCCGCATCGGCAAGGACGGCGAGAGCGGCTCCTACGACTTCTAG

>APQ4-like-2

ATGGGAGAACTAGGAGCCAAATTGGGCCTGGATGAGCTGGCTGGAGGCGCCGCCGGCATCAGCAGAGCACTCCTAGCAGAGTTCATAGGAAACATCCTACTGAACCTGTTCGGCTGCGGCGCGTGCATCAACGTGGCACAGGGCTCCTCGGCCGCGCCAGACATCGTGCTCATCGCGCTCGCCTTCGGGCTGGCGGTGTATGCCGCGGTGTCTGCGATAGGCCATGTCTCCGGTGGCCACATAAACCCAGCGGTTACCGTGGGTCTCGCCGCAGCGGGCCGCGTGAAGCCTGTGCGCGCCATCTTGTACATCATCGCGCAATGCGCGGGCGCCGCGGCAGGCTCAGGCCTACTGAAGGCCTTCACGCCTGATCGAGTCGCCGGCAGCCTCGGAGTCACCGGGCTTGGTGCTGACGTCACAGCACTCCAGGGCTTCGGCATCGAGTTCTTCCTTGGCTTCGTTCTTGTCTTCGTTGTTTGTGGGGTGTGCGACCAGCACAAGCCTGACAGCAAATCCACAGCACCCCTCGCCATCGGCCTGACCGTCACCCTCGGCCATCTGCTGGCTGTTGACTACACCGGCTCTGCCATGAACCCCGCCCGCTCCTTCGGATCTGCACTCGTCGCCAACGTCTGGGCCAACCACTGGGTGTACTGGGCGGGCCCAGTGGCCGGCGGAGTGGCGGCGGCTCTCCTCTACGTGCACGGCTTCTCGGCGCCGCCGCAGGAGACTCCGCGCTACAGGCCCGTGGCTGCCGACGAGAAAGAGCTGAAGCGACTTGACGGAGGCAAAGCGGACGATCTTGCCTGA

>transient receptor potential protein-like

GCAGGACGGAACCCGCGACGGTCTGCATGCCACCACCGGTGGTACCCCGACCATGACTTCAGGCTGACCTGTACTTCGATCAGGATGAACCGTGGCTCGAGGGAGGAGCTCCTGGGCAGCTCCCAGGAAGCCCTCAGGAACAGCCAGCATGACCTGAGGACCTTCAACGAGCGCGCCCTCACCAGAGAAGAGAAGACGTACCTCCTCCATGCTGATAGAGGAGACTACGCCACTGTTAAAAGGCTAATAGAACAGTACTCCAACAACACAGATGTCCTGGACATCAACTGTGTGGACCCTCTCAACCGGTCGGCGCTCATCGCCGCCATTGAAAATGAGAACATTGAGCTCATCAAACTGCTGCTGGACTCTGGGATTATGGTCAAGGATGCCCTGCTCCATGCAATTAAGGAAGAATACGTAGAGGCAGTAGAGCTACTTCTGCAGTGGGAAGAAGACCACCATGTACCCGGTGATCCTTACAGCTGGGAGTCAGTAGACCCTTCAGCGGCCACCTTCACGCCAGACATCACGCCTCTCATCCTGGCTGCCCACAGGAACCACTACGAGATCCTCAAGATACTCCTGGACAGGGGAGCGACTCTGCCAATCCCCCACGACGTCAAATGCGGCTGCGACGAGTGCGTCAAGTCCTCCCAAGAGGACTCGCTGCGGCACTCCCAGGCTCGCATCAACGCGTACCGCGCGCTTACCTCCCCCTCGCTCATCGCGCTGTCCTCAGCAGACCCGCTGCTGACTGCCTTCCAACTTTCCTGGGAGCTGAACCGGCTGAGCAGGATGGAGACGGAGTTCCGGGTCGAGTACAAGGCACTGCGGCAGCAATGCCAGGAGTTCGCCACCTCCCTCCTGGACCACACGAGGACGTCCAACGAGCTGGAGATCATGCTCAACTACAACCCCTGGGATACGGACAGCTGGGAGCCAGGAGAAAGGCAGACCCTGGGCAGGCTCAAACTGGCCATCAAGTACAAGCAGAAAATGTTCGTAGCTCATCCGAATGTACAACAGTTGCTGGGCGCGATTTGGTATGAAGGCTTACCAGGATTCAAGAGAAAAAACATCGTCGGGCAATGTGTTCAGGTAGCGAAACTTGGCGTGATGTTTCCCGTGTACTGCACGATCTACATGCTAGCGCCGAACTCTGAGTACGGCCGCTTCATGAAGAAGCCCTTCGTCAAGTTCATCTGCCACAGCTCCTCCTACATGTTGTTTCTAATGCTGCTCTCCCTCGCGTCACAACGGGCAGAGTACCTGGTCCTGGAATGGTCCGGGATCCGCTGGCTCCAAGAACTGGTGGAGCATTGGAAGGAGCACGAGCGAGGGTCTCTGCCGGGGCTCATCGAGTTCACCGTCATCATCTACATAGCAAGTCTCATCTGGGCCGAGATTCGCTCGCTCTGGACAGGAGGGCTGATGGAGTACATCAGTGACCTTTGGAATATCGTGGACTTCATTACCAACATGTTCTACATCGCCTGGATTAGCTTGCGGGTTGCTTCGTGGTATATCGTGCAGCGTGACTACAAAAGCGGCATGGACCCATGGTACCCCCGTGAACGCTGGGACTCCTACGACCCCATGCTGCTCTCCGAAGGTGCCTTCGCTGCTGGCATGATATTCTCCTTCCTCAAACTGGTGCACATCTTCTCCATCAACCCATACCTTGGGCCGCTGCAGGTGTCCCTTGGAAGAATGATCCTCGACATCATCAAGTTCTTCTTCGTTTATATGCTGGTGCTGTTCGCGTTTGGGTGTGGTGAGTCTTATAAGAATTATTGTTCTAGTTGGACTCATTATTCCATTTCTGGTTTCCTTACAAAATGCCAACCCTAA

>tyrosine aminotransferase-like 1

ATGTCGTCAGGTCGATCGCAGGACAAGCGGGCTTGGGAGGTGCGCGCGTCAGCTCTAGCTCGGAATACGCACAATCTGATAAGGAGCATCGTCGAAAATCTGCAAGTAGAGCCGAACCCCGAAAAACAACTGATAGCGCTTTCAGTAGGTGATCCAACGACTTTTGGGAACCTAAATCCGCCGGAGCAAGTGATCCAAGCTGTGCGGGAGAGTATAGAATGGCATACAAGTCGAGGGTATGGGTCCATGGTGGGCCACCCGGAGGCGCGCCAGGCGGTGGCGGAGTACAGCGCGCACCAGGGCCCCGTGGCCGCTGAGGACGTGATCCTCTGCAGCGGCTGCTCGCACGCCATCGAACTCGCCATCACGGTTCTCGCCGATTCCGGACAGAACGTTCTCGTCCCCCGACCCGGGTTCATGATATACAAAACTGTTGCCGAAGGATTGGGGATAGAAATCAAGTATTACAACTTACTGCCTAATCAACAATGGAAAGTCGATCTGGAGGATCTAGAAAATCAAATAGATGATGACACTGCCGCGATAGTCGTGATCAATCCGTCAAACCCATGTGGCTCGGTGTACAACAAGGAGCATTTGACTGAGATCATAGATATAGCTTCAAGGAACTGTGTGCCTTTAATTGCAGACGAGATATACGAACACTTTGTGTTTTCTGGCCATGAATTCACAGCTATTTCAGCACTATCGAAAGACGTACCTGTTCTGACGTGCGGTGGCCTTACCAAAAGATTTTTGGTGCCTGGCTGGAGGATGGGATGGCTGATCGTGCACGATCGGCAAAATATACTAGGCAAAGAAATTCGAAAGGGAATAAGCAATCTAGCAAGTAAAATACTCGGACCTAATACGCTAATACAGAGAGCCTTGCCGACTATACTCAAGACCACACCGCAGAGTTTCTTCGATGAGGTTGTCCTTTTTATCGAGAACCAAGCTAAACTGGCCTATGAGGAGCTACGACGAGCTCCAGGGCTCCGACCCATTATGCCACAAGGGGCGATGTACATGATGATTGAAATAAAAATGGCGCAGTTCCCTCAGTTCAGCAGCGAACTGCAGTTTGTGGAGCGAATGGTCTCAGAACAGTCCGTGTTCTGTCTGCCAGGAGAGTGCTTCTGCTATCCAAACTACATGCGCATCGTGTTAACTGTGCCAGAAGACATCATGAGGGAAGCGTGTCAGAGGATAGTCAAGTTTTGCCAAGATCACATCGTGACCAGAGAAAAGCTGAAAGAGATTGACACCAACGTGGTGTCCATGCCTAATGCAACTGAAGTCATTTGCGATGACGCACTGCCACGCGTAAATTAG

>tyrosine aminotransferase-like 2

ATGGCTGGCCAGGGAACAGAGAGACCGTGGCAGGTACGAGCAACATTTCTGGCTCGCAACACCAACAATTTAATCAGGAACGTCATTGAAAACCTGAAAGTTCAACTCAATCCGGAAAAGAAACTTATCCCGCTTTCTCTCGGTGACCCTACAGTTTTCGGGAATATCACCCCGCCTGAAGAACTGTTGCAAGCCGTTCGGGATAGCATAGATAATAACAAAAGCCGTACATATGGGCCCACTAATGGTCAGCTTGAGGCCCGTCAAGCTGTCGCAGACTACAGCATCCACCAGGGACGCGTGACGGCTGATGACGTCATACTCACCAGCGGATGCGCGCACGCACTGGAGCTCGCCATCACAGTGCTGGCTGAAGATGGACAAAATATCCTCGTGCCGAGACCAGGGTACATGATCTACATGACGCTAGCTGAGGGTCTGGGAATACAAATCAAGTACTACGACTTGTTGCCAGAACAAAAGTGGAAAGTAGACTTGGGGAGCTTACAGAAACAAATTGATGACAAAACTGCAGCCATTGTAGTAATTAACCCTTCAAATCCTTGTGGATCAGTTTACTCCGAGGAACACCTACTCGAAATACTGGACATCGCATCTAAGAACCGTGTGCCCATTATTGCCGATGAAATTTATGAGCATTTTGTGTTCTCAGGGCATAAGTACACGGCTATGTCTGCCCTTTCCAAGGATGTTCCTATTCTTACTTGTAGCGGACTCACAAAGAGGTTCTTAGTACCAGGCTGGCGAATGGGGTGGCTGATTGTACACGATCGGAACAACATTTTTGGGAAAGAAATTCGCCAAGGTTTGAGGAATTTATGCGGTAGACTACTTGGGCCTACAACGTTGATACAGCATGCCTTGCCTAGTATATTGACGTCCATGCCGCAGCATTTCTTCGATGGAGTAATGTTCTTCATAGAGAGCCAAGCGAAGTTAGCCTGCGAAGCCTTACAGCGGGCACCTGGCTTACGCCCGATAATGCCCCAAGGTTCAATGTACATGATGATAGAGATCAAAGTGTCTATGTTTTCCGAATTTCAGAACGATCTCCAGTTTATTGAACGCTTATACTCCGAGCAGTCCGTGCTCTGTATACCTGGACAGTGTTTTTACTATCCGAACTTCATGCGCATTGTACTGACGGTCCCTGAGGAAATTCTGCGTGAGGCGTGTCAAAGGATCATCGCATTTTGCAAGGACCACGCAACATCGAAAGAAAAACACATGGAAATGATTACGAACAAGTTATCGGTTGATAAAGAATGGAGGGTTATTTATTAA

>phenoloxidase subunit A3-like

TTCATGCAGAAGGGCGACGACAAGACTGTCTTCCAGCTGCCCGACCATTACTACCCGGACAAATACAAGGCGTTGAGCACGACGCTGTCGGACCGCTTCGGGACCGACGACGCCAGGATCGTTCAGGTTTCCAACATCGGGCTGCCCGACCTGAGCCTGCCGAAGCAGCTGCCTTACAACGAGCAGTTCTCGCTTTTCATCCAGAAGCACAGGGAGATGGCTGGCAGCCTCATCGACACCCTTGTCGGCATGCGCAACATAGAGGATCTCACTTCGGTTTGTTCATATTGCCAGCTGCATATCAACCCTTACATGTTCAACTACTGCCTCGCAGTTGCACTTCTGCACAGAGACGACACCAAGGGTTTCAACGTACCCACTGTGGTCCAAACCTTCCCAGACAAGTTCATGGACCCCAAGGTGTTCCGCAGGGCTAGGGAGGTGTCTAACGTCGTGACGACAGGCCCCAGGATGCCGGTCGTGATCGAGCAGAATTACACAGCCTCAGACGCGGAGCCCGAGCAGCGCGTGGCTTACTTCCGCGAGGACATTGGCATCAACCTGCACCACTGGCACTGGCATTTGGTGTATCCCTTCGAAGCCGACTTCGCCATCGTCAACAAGGACAGGAGGGGAGAACTCTTCTATTATATGCACCAGCAGATCATTGCCAGATACAATGTGGAGCGCTTCTGCAACGGGCTGGGCCGCGTGGAGCGATACACCAACTTCCGAGCCCCCATTGCGGAGGCTTATTTCCCCAAACTGGACTCCCAAGTCGCCAGCCGTGCTTGGCCTCCAAGGTTCGCGGGTTCGATCCTCCGCGATCTGGACCGTCCTGTTGACCGGATCAGGATTGAAGTCTCCGAGCTGGAGAGATGGAGGGATCGCTTCCTCCAGGCTATTGAAGAAAACGCTGTACTAGTGCCTGGAAACCGCAAGGTGCCTCTTACTGAGGAGACAGGAATTGATGTGCTCGGCAACTTGATGGAGTCGTCTATCCTGAGCCGTAACCGCGGCTACTATGGGGACCTGCACAACATGGGCCATGTCTTCTGCTCGTACGCTCACGACCCTGACCATCGCCACTTGGAGCAATACGGCGTGATGGGAGACTCGGCCACCGCCATGCGAGACCCGTTCTTCTACCGCTGGCACGCGTACGTTGATGACATCTTCAACATGCACAAGGTTAAGCTGCCGAAGTATGGCGCTGACAGGCTGGACTTCCCTGGTATCCGCGTGTCTTCTATCAGCGTGGAGGGTCCCGCCGGTAGGAACACCTTCGGCACTCAGTGGGAGCAGAGCACGGTGGACCTGGCCAGGGGACTCGACTTCACTCCGAGGGGCAGCGTGCTGGCACAGTTCACCCACCTCACGCACGAGGAGTTCACTTATGTCATCGAGGTGAACAACTCCACCGGGCGATCCACCACGGGCATGTTCCGGATCTTCATCGCGCCGGTGAACGACGACCGCGGGCAGCCTCTCAGCTTCGCAGACCAGAGGCGGCTCTTCATCGAGCTCGACAAGTTCTCGCAGCCACTGAATACAGGCAATAACACAGTCCGTCGCAGCAGCGTCGACTCCTCCGTGACCATCCCCTACGAGCGGACTTTCATGGACCAGACGAAGCGCCCTGGAGACCCGGGCTCCGCCACCGCGGCGGAGTTCGACTTCTGCGGCTGCGGTTGGCCGCACCACATGCTGCTCCCCAAGGGAACCGCGCGAGGGTACCCCATGGTGCTATTCGTGATGGTGTCCAACTGGAACGACGATCGCATCCTGCAAGACACGTCAGGCTCTTGCAACGACGCAGCCTCGTACTGCGGGATCCGCGACCGAAAGTATCCGGACAAGCGCGCCATGGGCTTCCCGTTCGACCGCCCTCCCCAAGAATCCACCCTGAGTGACTTCTTACGCCCCAACATGTCCATCAGACAGTGCACTGTGAAGTTCACGGACGCTACAAGGATCAGGCAGCAGCGCCGGTAG

S4. PPI network of DEG in male brain corresponds to Cytoscape data

| **Number** | **Name** | **Average Shortest Path Length** | **Clustering Coefficient** | **Closeness Centrality** | **Degree** | **Betweenness Centrality** | **Neighborhood Cnnectivity** |
| --- | --- | --- | --- | --- | --- | --- | --- |
| 1 | ALiX | 1 | 0 | 1 | 3 | 1 | 1 |
| 2 | Act5C | 1.631579 | 0.266667 | 0.612903 | 10 | 0.403509 | 5.4 |
| 3 | Mlc1 | 1.473684 | 0.509091 | 0.678571 | 11 | 0.278752 | 6.727273 |
| 4 | l(2)efl | 2.105263 | 0.3 | 0.475 | 5 | 0.204678 | 6.2 |
| 5 | Myo61F | 1.894737 | 0.5 | 0.527778 | 4 | 0.19883 | 8 |
| 6 | Cad88C | 2.736842 | 0 | 0.365385 | 2 | 0.105263 | 2.5 |
| 7 | Mhc | 1.684211 | 0.722222 | 0.59375 | 9 | 0.076998 | 7.888889 |
| 8 | Mlc2 | 1.684211 | 0.722222 | 0.59375 | 9 | 0.076998 | 7.888889 |
| 9 | Prm | 1.684211 | 0.722222 | 0.59375 | 9 | 0.062378 | 7.777778 |
| 10 | wupA | 1.842105 | 0.857143 | 0.542857 | 8 | 0.018519 | 8.375 |
| 11 | Mf | 2.105263 | 0.952381 | 0.475 | 7 | 9.75E-04 | 8.285714 |
| 12 | pUf68 | 1.666667 | 0 | 0.6 | 1 | 0 | 3 |
| 13 | Stam | 1.666667 | 0 | 0.6 | 1 | 0 | 3 |
| 14 | CG6180 | 1.666667 | 0 | 0.6 | 1 | 0 | 3 |
| 15 | Abl | 2.578947 | 0 | 0.387755 | 1 | 0 | 10 |
| 16 | CG5525 | 2.578947 | 0 | 0.387755 | 1 | 0 | 10 |
| 17 | FASN1 | 2.578947 | 0 | 0.387755 | 1 | 0 | 10 |
| 18 | kst | 2.578947 | 0 | 0.387755 | 1 | 0 | 10 |
| 19 | CG13830 | 1 | 0 | 1 | 1 | 0 | 1 |
| 20 | Socs16D | 1 | 0 | 1 | 1 | 0 | 1 |
| 21 | dysc | 3.684211 | 0 | 0.271429 | 1 | 0 | 2 |
| 22 | CtsB1 | 1 | 0 | 1 | 1 | 0 | 1 |
| 23 | KLHL18 | 1 | 0 | 1 | 1 | 0 | 1 |
| 24 | Hsp68 | 3.052632 | 0 | 0.327586 | 1 | 0 | 5 |
| 25 | fln | 2.157895 | 1 | 0.463415 | 6 | 0 | 8.833333 |
| 26 | TpnC25D | 2.157895 | 1 | 0.463415 | 6 | 0 | 8.833333 |
| 27 | PebIII | 2.421053 | 0 | 0.413043 | 1 | 0 | 11 |
| 28 | tj | 3.052632 | 0 | 0.327586 | 1 | 0 | 5 |

S5. PPI network of DEG in female brain corresponds to Cytoscape data

| **Number** | **Name** | **Average Shortest Path Length** | **Clustering Coefficient** | **Closeness Centrality** | **Degree** | **Betweenness Centrality** | **Neighborhood Cnnectivity** |
| --- | --- | --- | --- | --- | --- | --- | --- |
| 1 | PPO2 | 2 | 0.1 | 0.5 | 5 | 0.64583333 | 2.8 |
| 2 | GNBP3 | 2.4375 | 0.16666667 | 0.41025641 | 4 | 0.43333333 | 2.75 |
| 3 | Ppn | 2.6875 | 0.16666667 | 0.37209302 | 4 | 0.28333333 | 2.25 |
| 4 | Spn77Ba | 2.3125 | 0.16666667 | 0.43243243 | 4 | 0.25416667 | 2.75 |
| 5 | Hml | 2.375 | 0 | 0.42105263 | 2 | 0.24583333 | 4.5 |
| 6 | Ddc | 2.8125 | 0 | 0.35555556 | 2 | 0.125 | 3 |
| 7 | PGRP-SC2 | 3.25 | 0 | 0.30769231 | 2 | 0.125 | 2.5 |
| 8 | sls | 3.5 | 0 | 0.28571429 | 2 | 0.125 | 2.5 |
| 9 | vkg | 2.6875 | 0.33333333 | 0.37209302 | 3 | 0.12083333 | 3.33333333 |
| 10 | Atg8a | 1 | 0 | 1 | 1 | 0 | 1 |
| 11 | CtsB1 | 1 | 0 | 1 | 1 | 0 | 1 |
| 12 | CG1213 | 1 | 0 | 1 | 1 | 0 | 1 |
| 13 | Tret1-2 | 1 | 0 | 1 | 1 | 0 | 1 |
| 14 | CG13868 | 1 | 0 | 1 | 1 | 0 | 1 |
| 15 | CG6428 | 1 | 0 | 1 | 1 | 0 | 1 |
| 16 | CG5873 | 1 | 0 | 1 | 1 | 0 | 1 |
| 17 | Cpr97Eb | 1 | 0 | 1 | 1 | 0 | 1 |
| 18 | CG8745 | 1 | 0 | 1 | 1 | 0 | 1 |
| 19 | dom | 1 | 0 | 1 | 1 | 0 | 1 |
| 20 | Cg25C | 3.375 | 1 | 0.2962963 | 2 | 0 | 3.5 |
| 21 | Pu | 3.75 | 0 | 0.26666667 | 1 | 0 | 2 |
| 22 | PGRP-LB | 3.3125 | 1 | 0.30188679 | 2 | 0 | 3 |
| 23 | PGRP-SD | 3.3125 | 1 | 0.30188679 | 2 | 0 | 3 |
| 24 | LysP | 4.1875 | 0 | 0.23880597 | 1 | 0 | 2 |
| 25 | MP1 | 3.25 | 0 | 0.30769231 | 1 | 0 | 4 |
| 26 | yellow-d2 | 2.6875 | 1 | 0.37209302 | 2 | 0 | 4.5 |
| 27 | ninaC | 4.4375 | 0 | 0.22535211 | 1 | 0 | 2 |

S6. Cytoscape data of PPI network of 24 brain development candidate genes

| **Number** | **Name** | **Average Shortest Path Length** | **Clustering Coefficient** | **Closeness Centrality** | **Degree** | **Betweenness Centrality** | **Neighborhood Cnnectivity** |
| --- | --- | --- | --- | --- | --- | --- | --- |
| 1 | wg | 1.5 | 0.26666667 | 0.66666667 | 6 | 0.56666667 | 2.83333333 |
| 2 | dac | 1.6 | 0.3 | 0.625 | 5 | 0.51111111 | 3.2 |
| 3 | Lim1 | 2.3 | 0 | 0.43478261 | 2 | 0.2 | 3 |
| 4 | Rbp9 | 2.2 | 0 | 0.45454545 | 2 | 0.2 | 3.5 |
| 5 | hh | 1.8 | 0.66666667 | 0.55555556 | 4 | 0.05555556 | 4 |
| 6 | fd102C | 3.2 | 0 | 0.3125 | 1 | 0 | 2 |
| 7 | sim | 2.4 | 0 | 0.41666667 | 1 | 0 | 6 |
| 8 | fne | 3.1 | 0 | 0.32258065 | 1 | 0 | 2 |
| 9 | Bx | 2.5 | 0 | 0.4 | 1 | 0 | 5 |
| 10 | Sox21a | 1 | 0 | 1 | 1 | 0 | 1 |
| 11 | Sox21b | 1 | 0 | 1 | 1 | 0 | 1 |
| 12 | run | 2.3 | 1 | 0.43478261 | 2 | 0 | 5 |
| 13 | ci | 1.9 | 1 | 0.52631579 | 3 | 0 | 5 |
